# Supplementary material for: Significant variation in the performance of DNA methylation predictors across data preprocessing and normalization strategies
Source: Genome Biol. 2022 Oct 24;23:225. doi: 10.1186/s13059-022-02793-w (PMC9590227; doi:10.1186/s13059-022-02793-w)
Supplement: Supplementary file 1 — Additional file 1: Supplementary figures 1-6. [file 13059_2022_2793_MOESM1_ESM.docx]

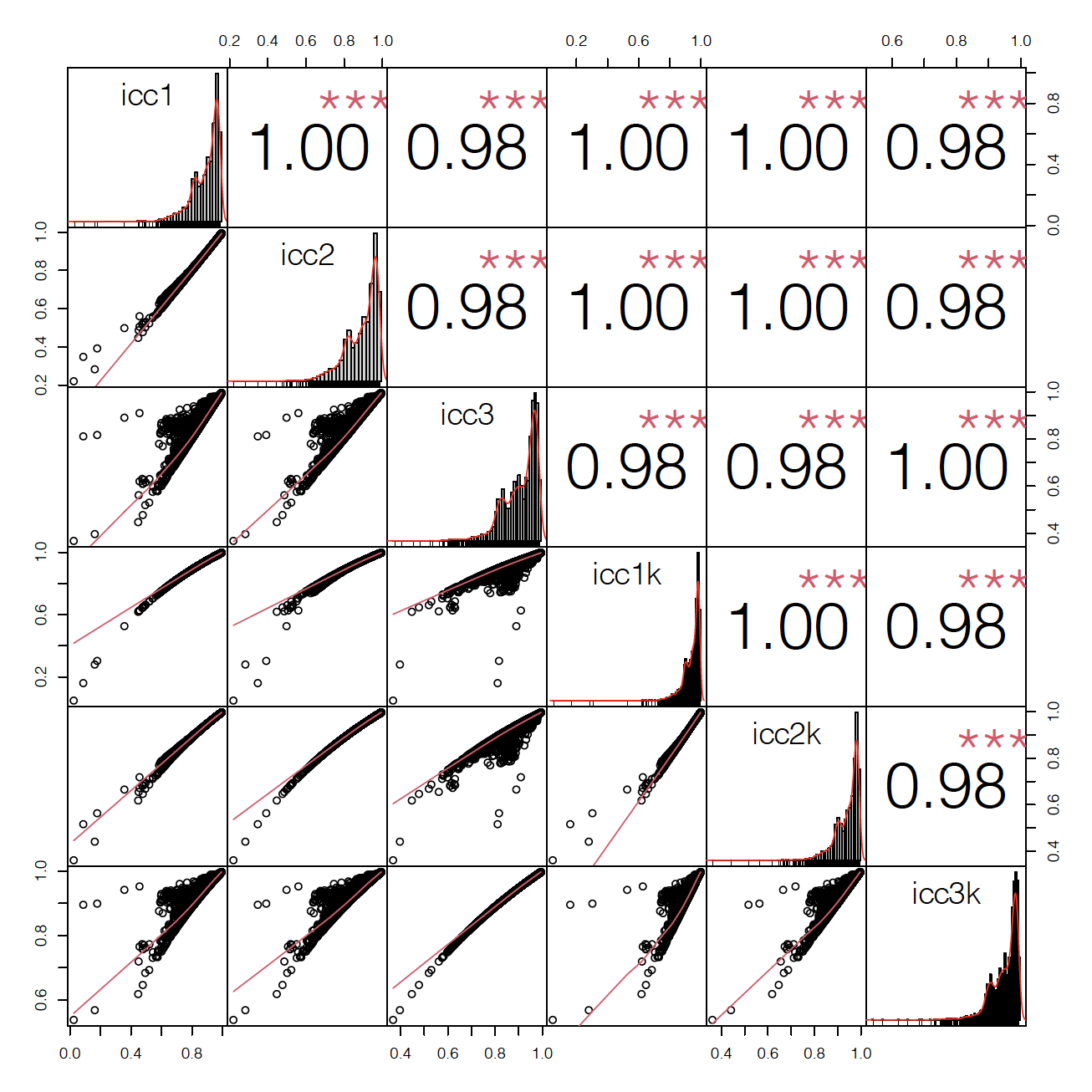


**Figure S1. Comparative analysis of ICC types across DNAm-based predictors and data processing pipelines.** Shown are the bivariate scatterplots (left bottom) and the Spearman correlation (right top) between ICC types across all pipelines and predictors (N= 101x41 = 4141). The distribution of each ICC type is shown on the diagonal. ***P-values < 2.2e-16. This figure was made using the chart.Correlation() function of the PerformanceAnalytics R package (v2.0.4).

**
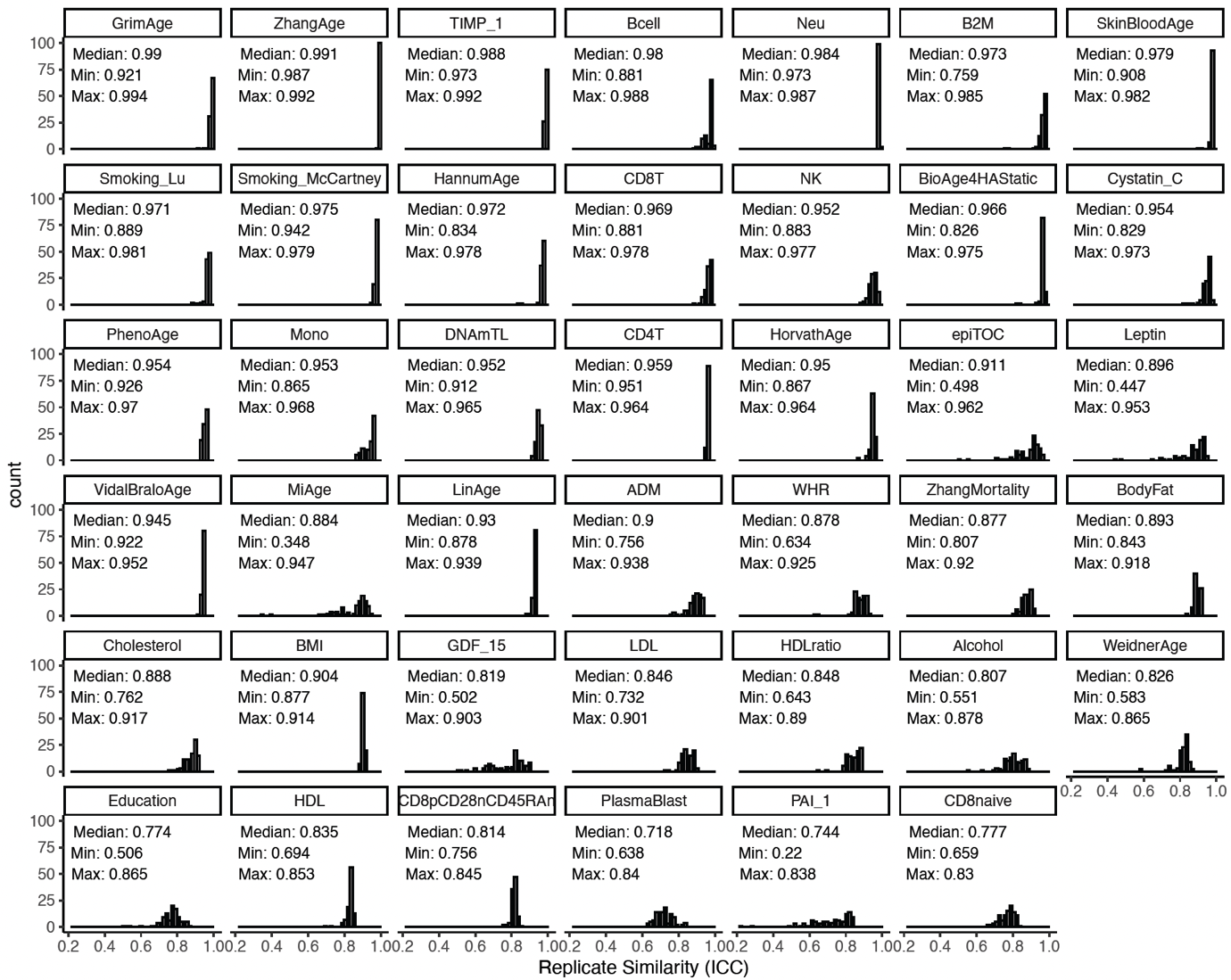
**

**Figure S2. The distribution of intraclass correlations across pipelines for each DNAm algorithm.** For each predictor, a histogram of ICC values across 101 pipelines is shown. The ICC quantifies the degree of absolute agreement between estimator values of a pair of technical replicates. The predictors are ranked based on their max ICC value. The name of the predictor is printed on top. In each panel, the median, lowest, and highest ICC value of a corresponding data processing pipeline for that predictor is shown as well.


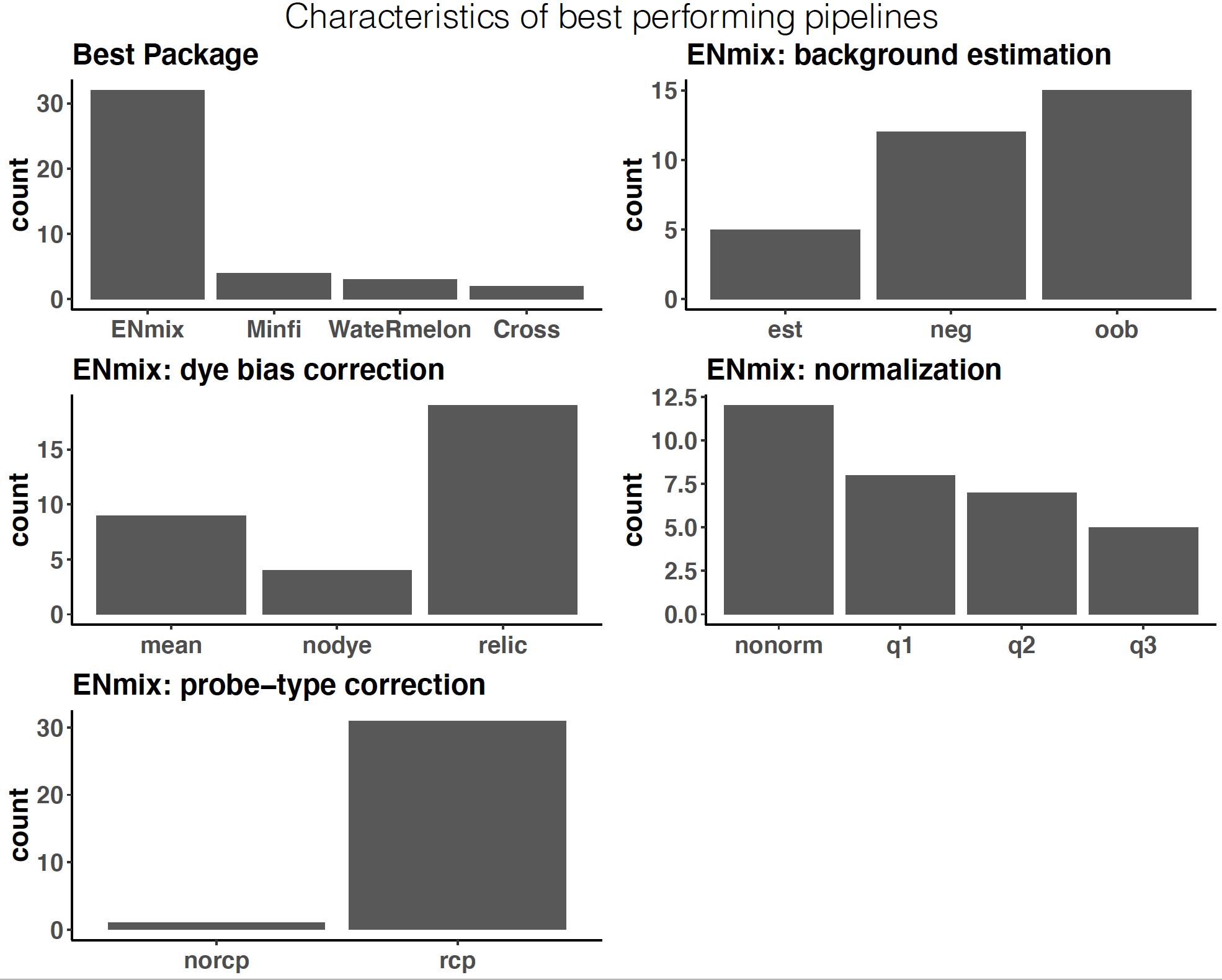


**Figure S3. Characteristics of best performing pipelines of predictors.** These graphs are based on the 42 best performing data processing pipelines (i.e., pipeline with the highest consistency of each predictors). Top left shows the corresponding package. 32 out of 41 pipelines are part of the Enmix package. The top right shows which background estimations ranked among the 32 Enmix pipelines. Middle left shows the ENmix dye bias correction method. Middle right shows the Enmix normalization method. The bottom graph shows if a pipeline used probe-type bias correction (i.e. “RCP method”).

**
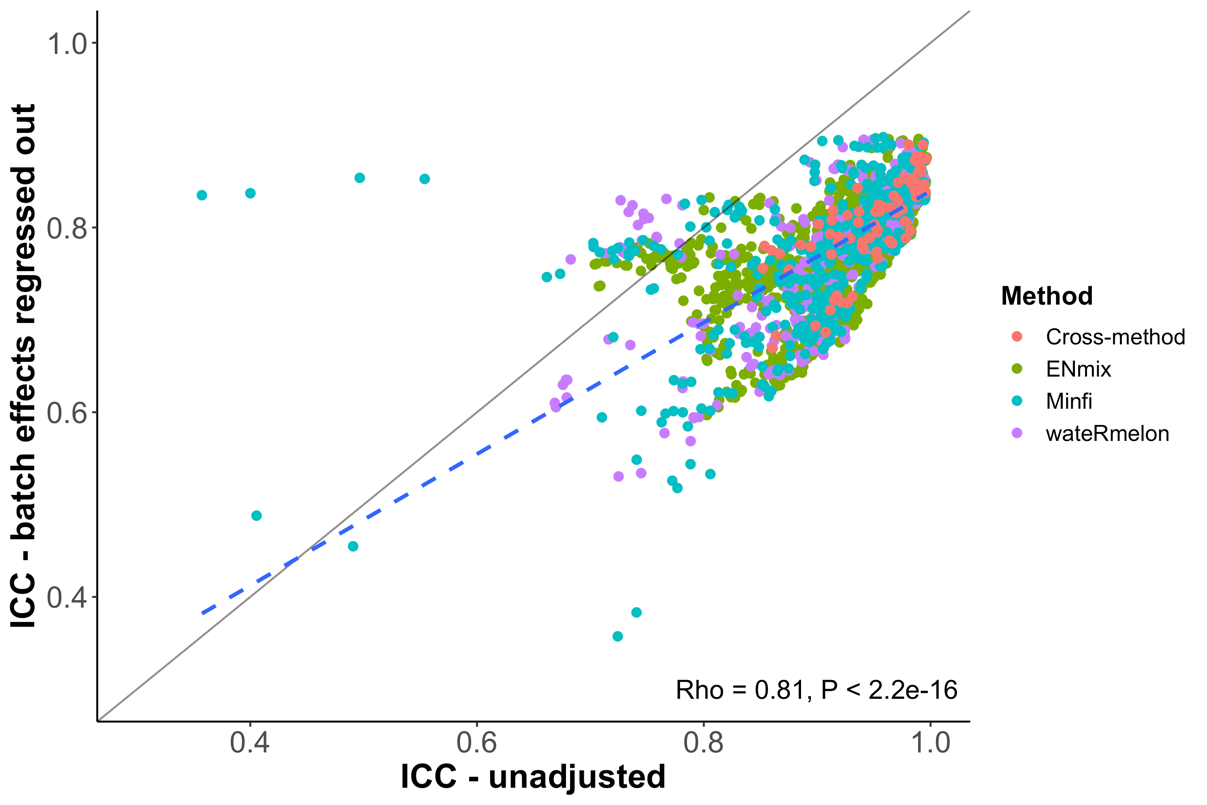
**

**Figure S4. Consistency between replicates is strongly correlated between predictor estimates adjusted and unadjusted for batch effects.** Shown is scatter plots of the relationships between consistency calculated with (y-axis) and without (x-axis) correction for batch effects across predictors and analytical pipelines (N=4141). The pipelines are color-coded by the corresponding R package. The solid line represents the diagonal while the dashed line represents a regression line obtained by geom_smooth(method=”lm”). The Spearman correlation statistics are shown in the right bottom of the plot.


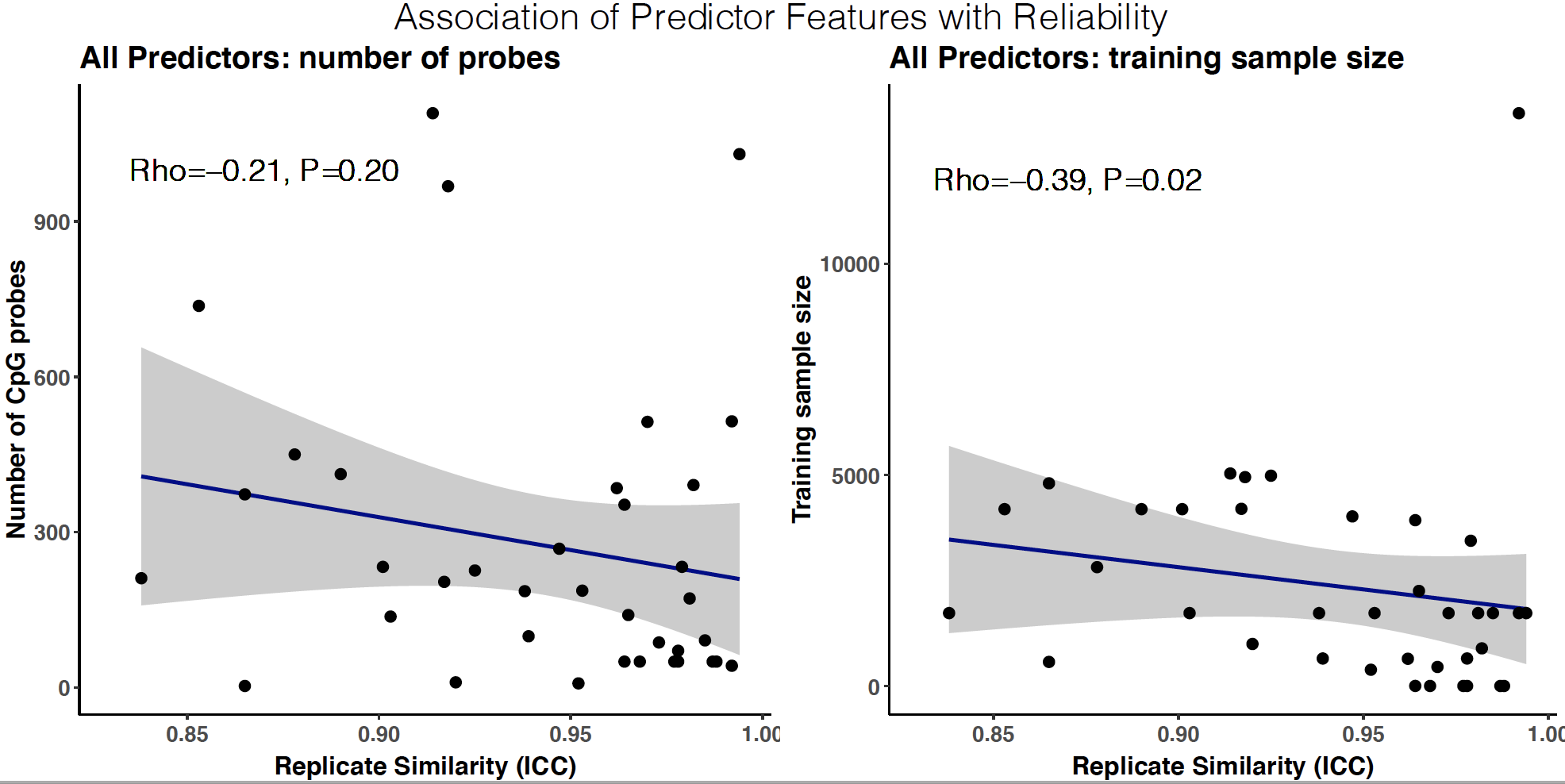


**Figure S5. Association of predictor features with predictor consistency.** Shown are scatter plots of the relationships between predictor features (i.e., training sample size and number of CpG probes) and their consistency (i.e., ICC) of the best performing pipeline for each predictor. Shown are the statistics of the correlation test (method=”spearman”) and a corresponding regression line.


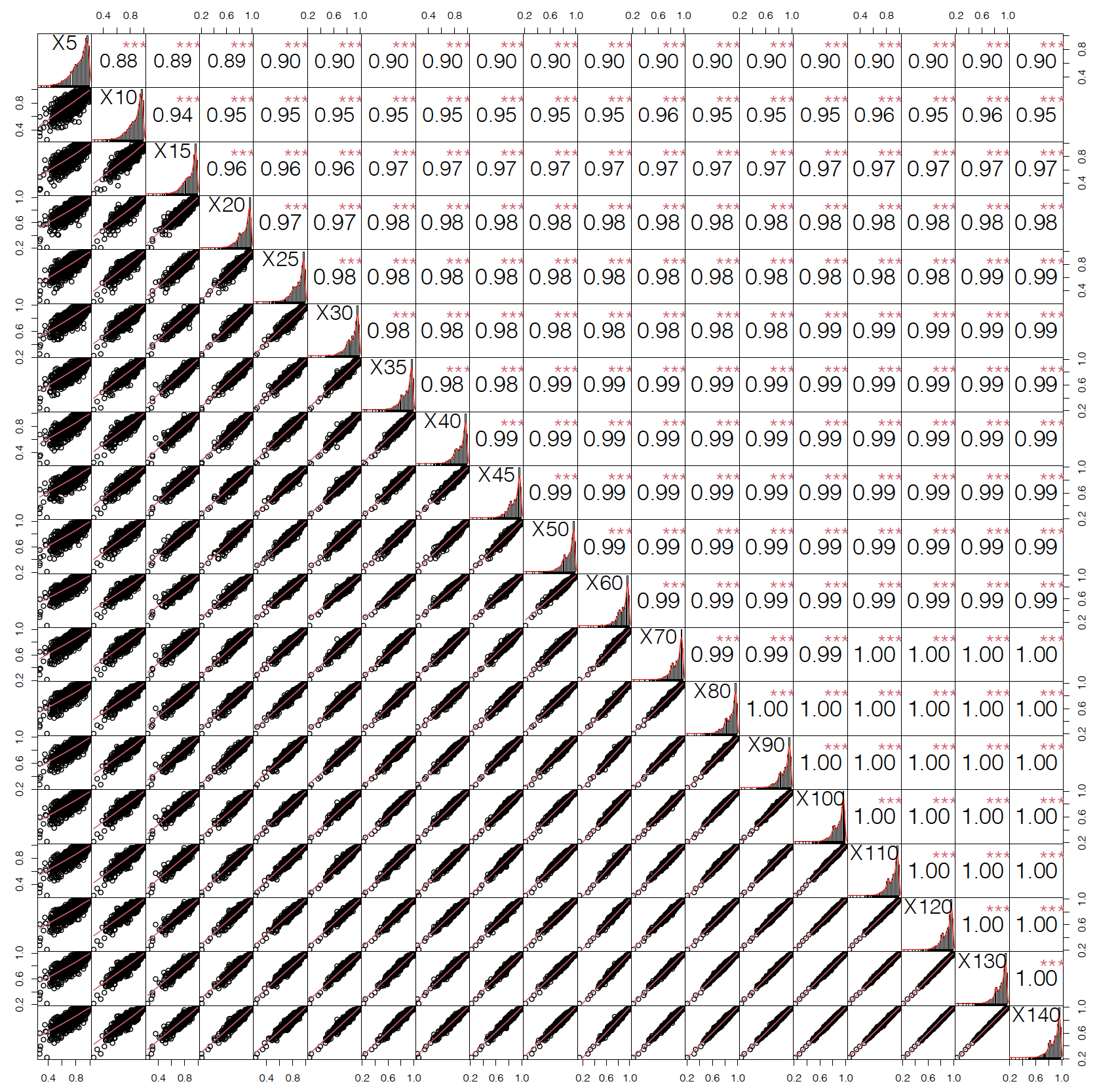


**Figure S6. Consistency measures across different sample sizes of replicate pairs.** Shown are the bivariate scatterplots (left bottom) and the Spearman correlation (right top) between the interclass correlations (all pipelines and predictors (N= 101x41 = 4141)) obtained across different sample sizes of replicate pairs. The sample size of the set of replicate pairs is shown on the diagonal across. For each sample size, we performed a bootstrap analysis in which we randomly selected the specified number of pairs from the total of 146 replicate pairs and computed the intraclass correlation across ten independent samplings. We then computed the mean intraclass correlation across these ten samplings and correlated this obtained mean ICC across different sets of replicate pairs. ***P-values < 2.2e-16. This figure was made using the chart.Correlation() function of the PerformanceAnalytics R package (v2.0.4).
