# Supplementary material for: Significant variation in the performance of DNA methylation predictors across data preprocessing and normalization strategies
Source: Genome Biol. 2022 Oct 24;23:225. doi: 10.1186/s13059-022-02793-w (PMC9590227; doi:10.1186/s13059-022-02793-w)
Supplement: Supplementary file 5 — Additional file 5. Figures that visualize the relationship between predictor estimates and replicate similarity (ICC) for each of the 41 DNAm predictors. [file 13059_2022_2793_MOESM5_ESM.pdf]

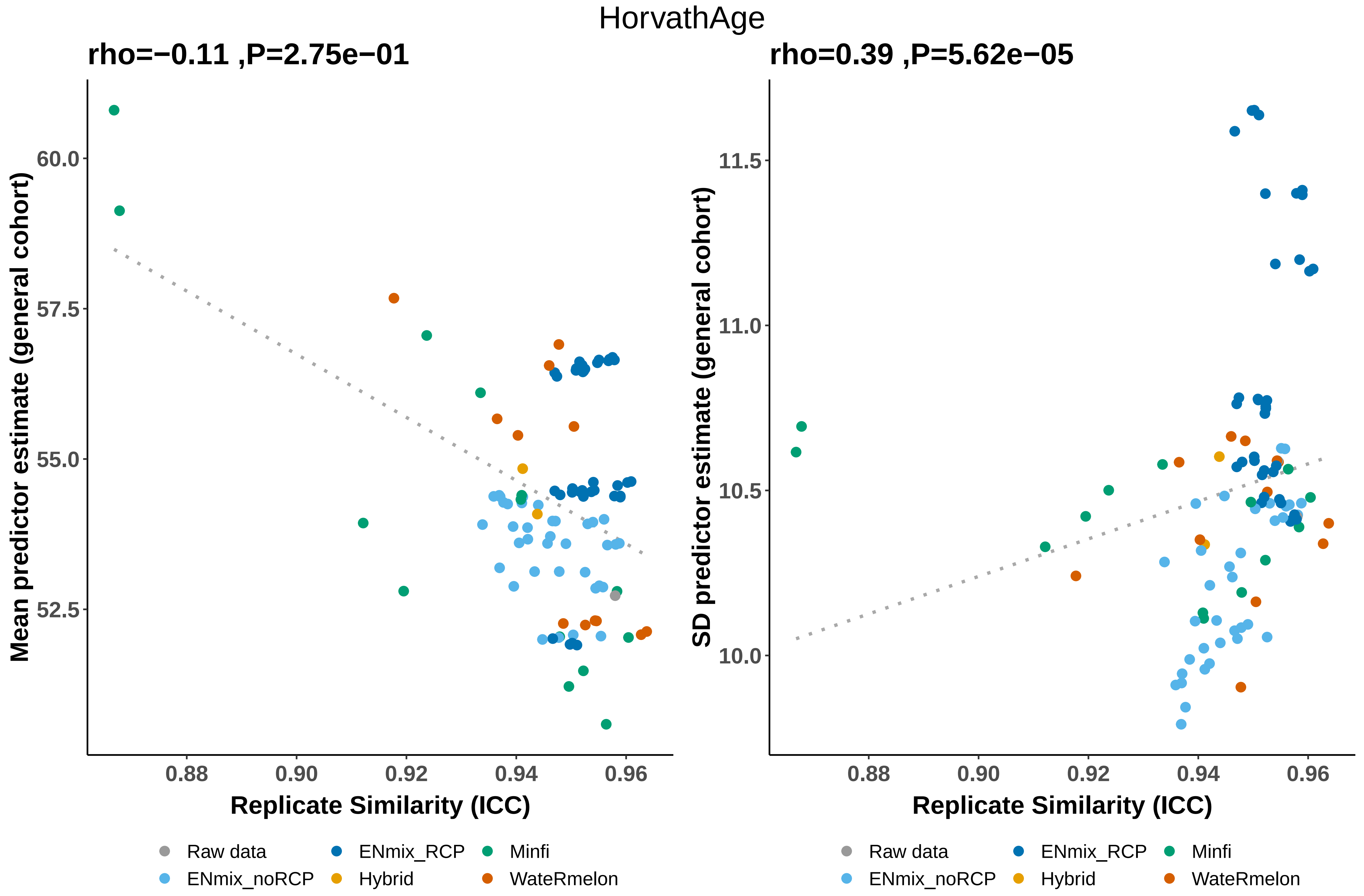

HorvathAge
rho=−0.11 ,P=2.75e−01
rho=0.39 ,P=5.62e−05
Mean predictor estimate (general cohort)
SD predictor estimate (general cohort)
60.0
57.5
55.0
52.5
11.5
11.0
10.5
10.0
0.88
0.90
0.92
0.94
0.96
Replicate Similarity (ICC)
Raw data
ENmix_RCP
Minfi
ENmix_noRCP
Hybrid
WateRmelon

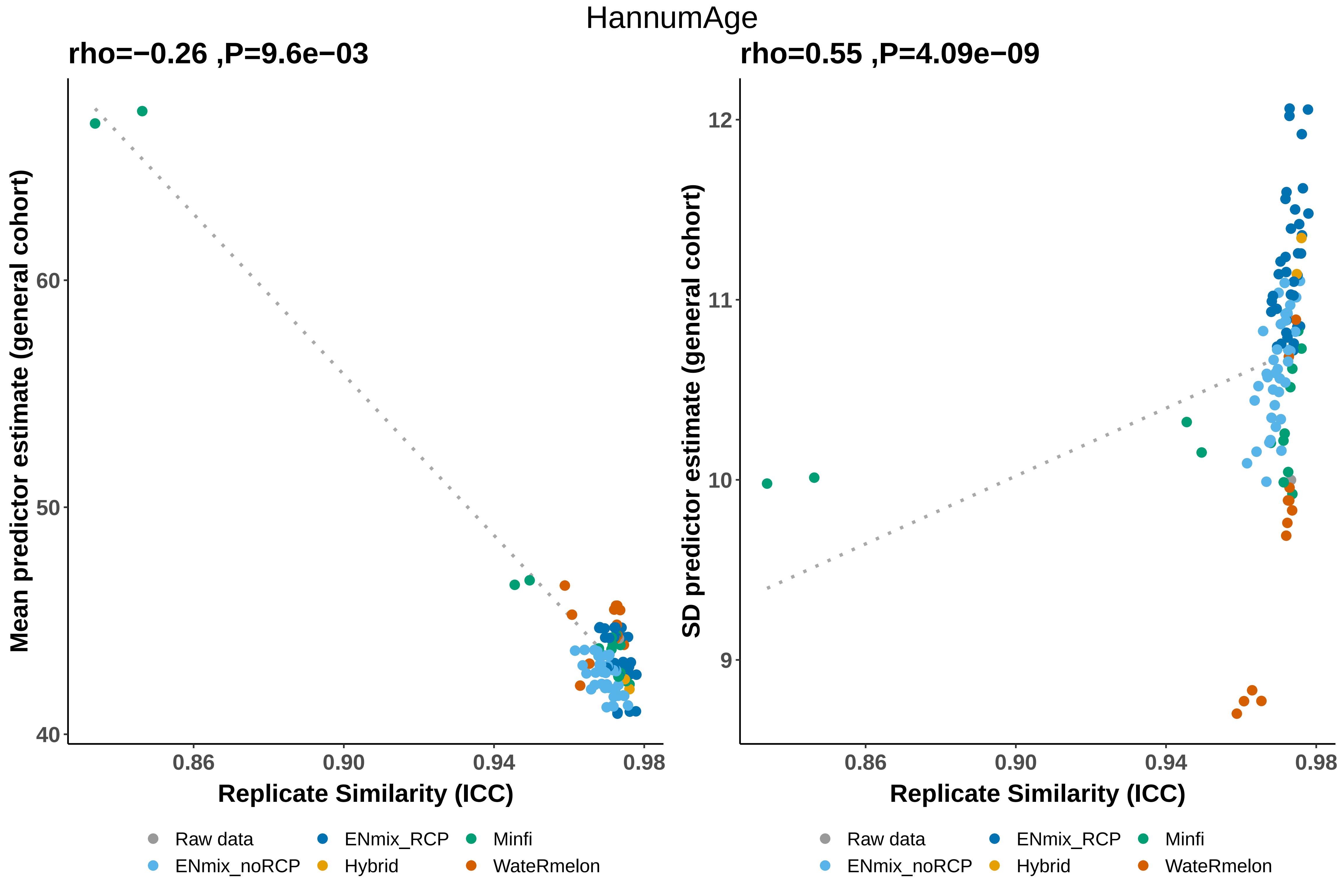

HannumAge
rho=−0.26 ,P=9.6e−03
rho=0.55 ,P=4.09e−09
Mean predictor estimate (general cohort)
SD predictor estimate (general cohort)
Replicate Similarity (ICC)
Replicate Similarity (ICC)
40
50
60
9
10
11
12
0.86
0.90
0.94
0.98
Raw data
ENmix_noRCP
ENmix_RCP
Hybrid
Minfi
WateRmelon

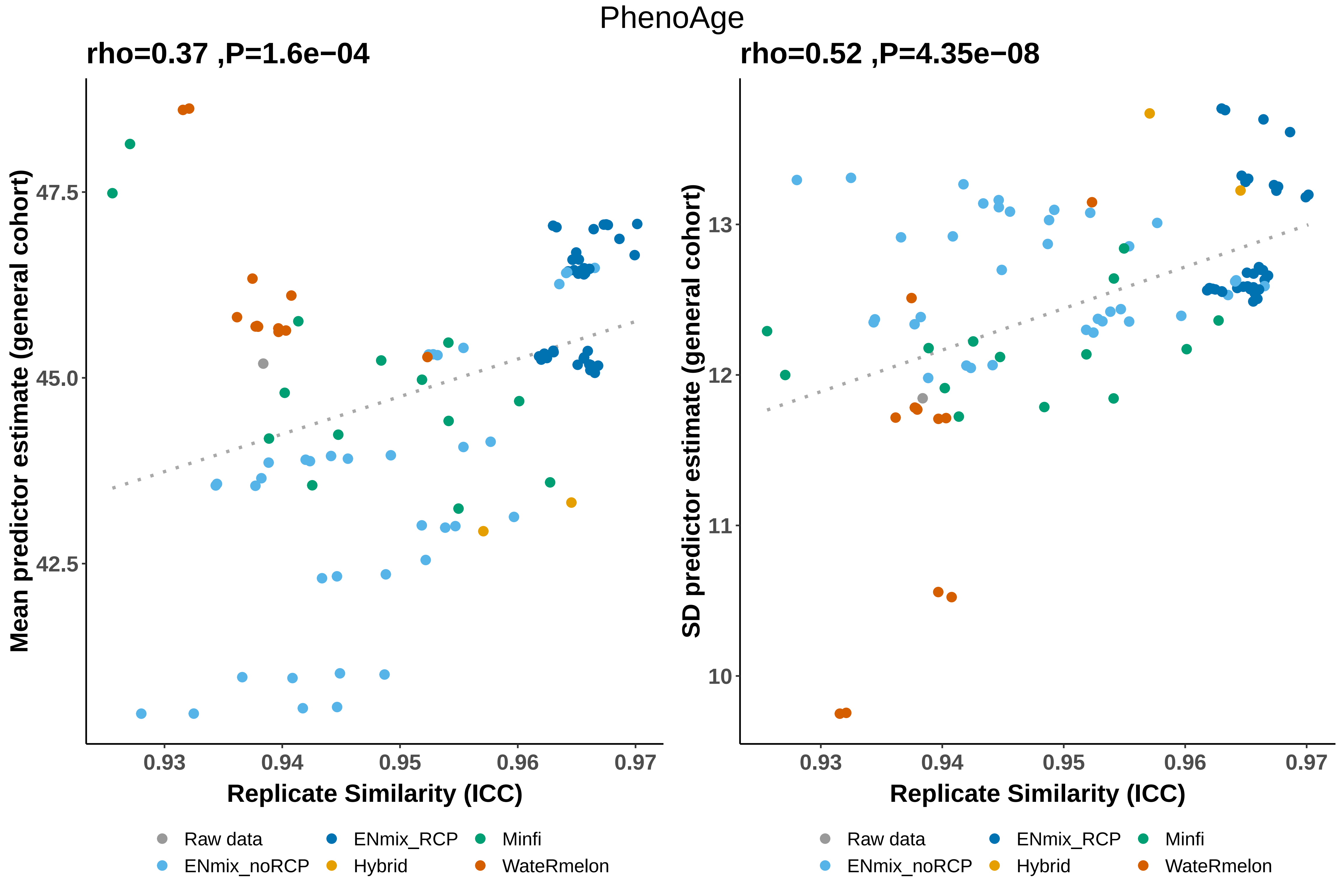
PhenoAge
rho=0.37 ,P=1.6e−04
rho=0.52 ,P=4.35e−08
Mean predictor estimate (general cohort)
SD predictor estimate (general cohort)
47.5
45.0
42.5
13
12
11
10
0.93
0.94
0.95
0.96
0.97
Replicate Similarity (ICC)
Raw data
ENmix_noRCP
ENmix_RCP
Hybrid
Minfi
WateRmelon

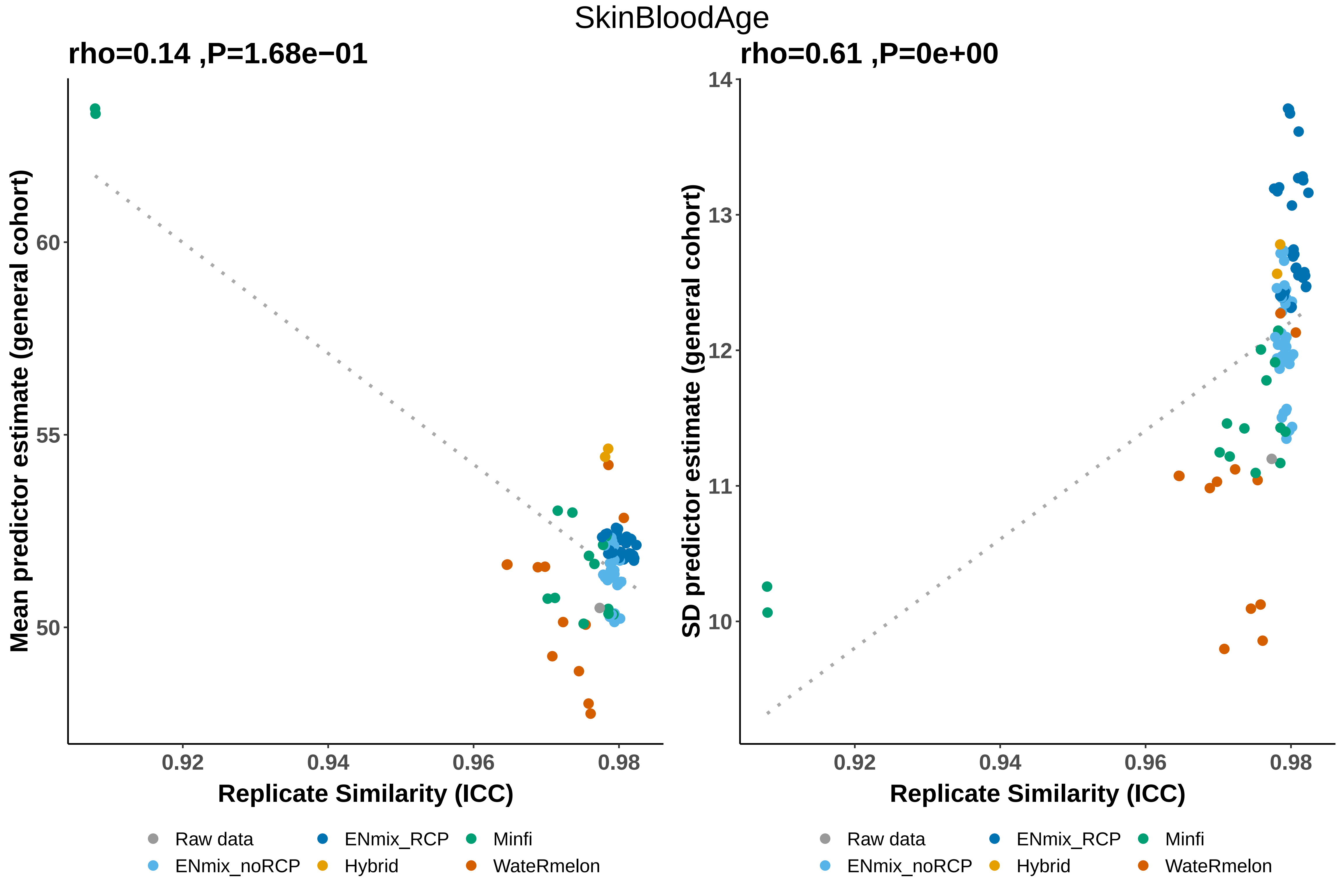

SkinBloodAge
rho=0.14 ,P=1.68e−01
rho=0.61 ,P=0e+00
Mean predictor estimate (general cohort)
SD predictor estimate (general cohort)
Replicate Similarity (ICC)
Replicate Similarity (ICC)
50
55
60
10
11
12
13
14
0.92
0.94
0.96
0.98
Raw data
ENmix_noRCP
ENmix_RCP
Hybrid
Minfi
WateRmelon

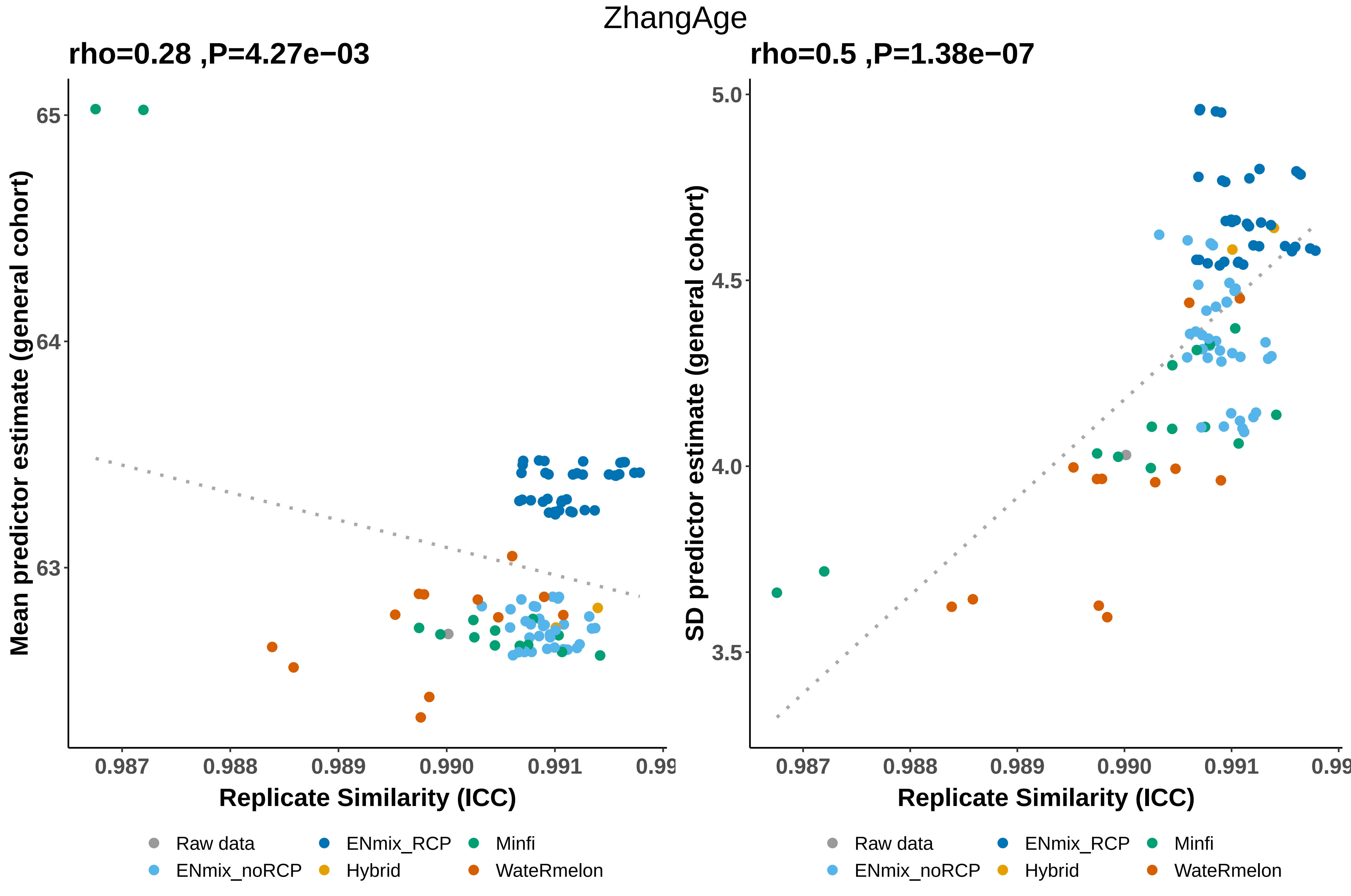

ZhangAge
rho=0.28 ,P=4.27e−03
rho=0.5 ,P=1.38e−07
Mean predictor estimate (general cohort)
SD predictor estimate (general cohort)
65
64
63
5.0
4.5
4.0
3.5
0.987
0.988
0.989
0.990
0.991
Replicate Similarity (ICC)
Replicate Similarity (ICC)
Raw data
ENmix_RCP
Minfi
ENmix_noRCP
Hybrid
WateRmelon

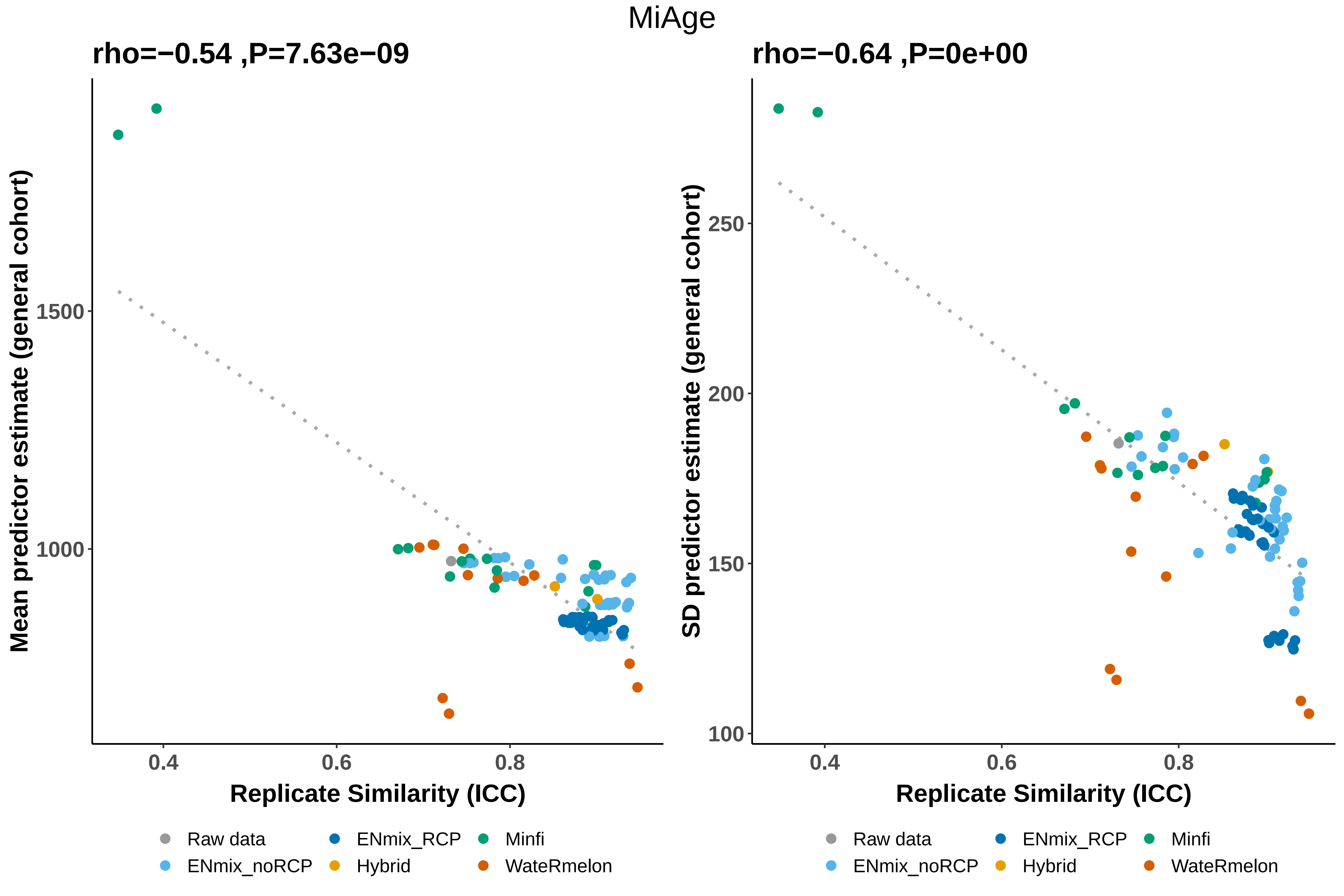

MiAge
rho=−0.54 ,P=7.63e−09
Mean predictor estimate (general cohort)
1500
1000
0.4
0.6
0.8
Replicate Similarity (ICC)
rho=−0.64 ,P=0e+00
SD predictor estimate (general cohort)
250
200
150
100
0.4
0.6
0.8
Replicate Similarity (ICC)
Raw data
ENmix_noRCP
ENmix_RCP
Hybrid
Minfi
WateRmelon

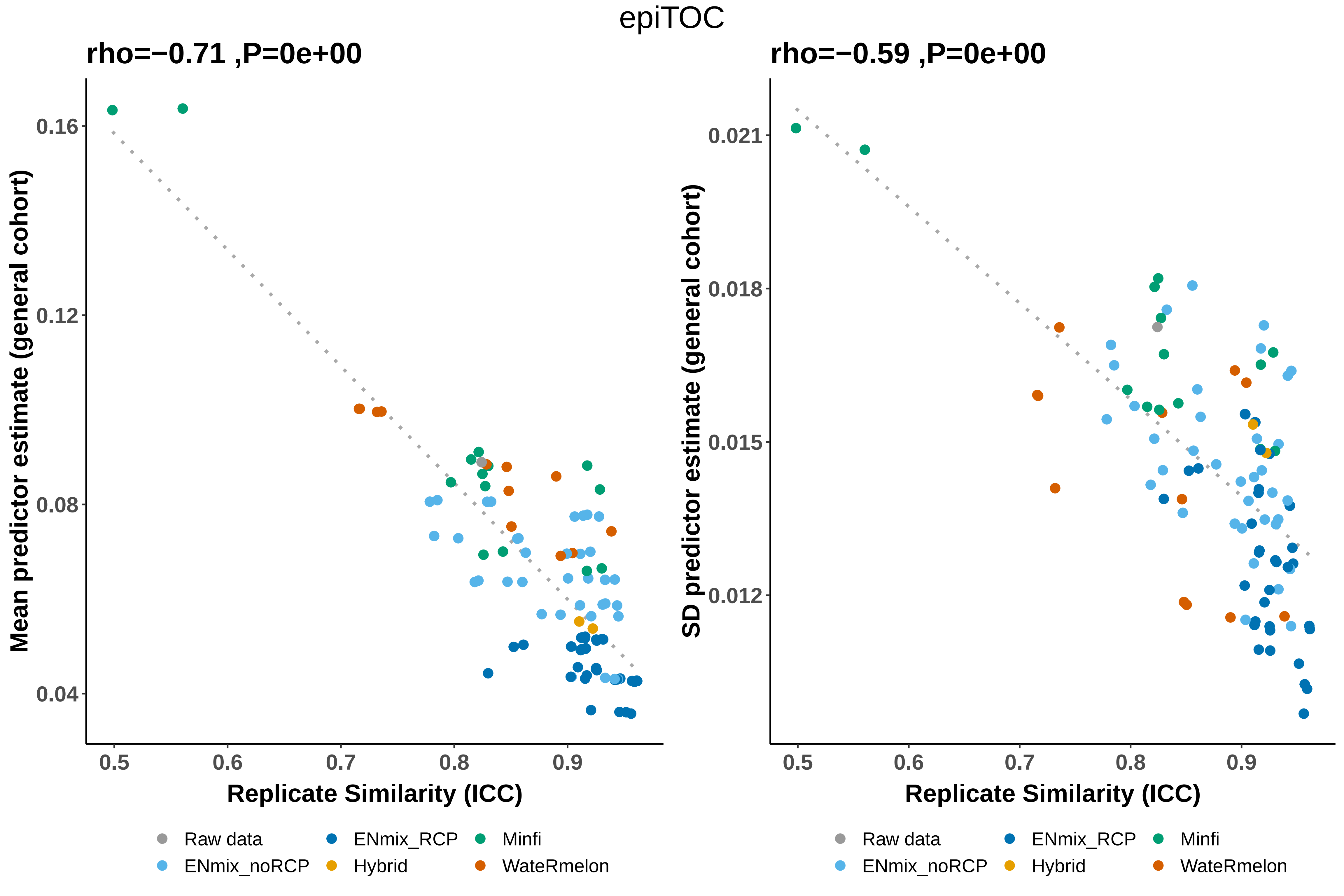

epiTOC
rho=−0.71 ,P=0e+00
rho=−0.59 ,P=0e+00
Mean predictor estimate (general cohort)
SD predictor estimate (general cohort)
0.16
0.12
0.08
0.04
0.021
0.018
0.015
0.012
0.5
0.6
0.7
0.8
0.9
Replicate Similarity (ICC)
Raw data
ENmix_noRCP
ENmix_RCP
Hybrid
Minfi
WateRmelon

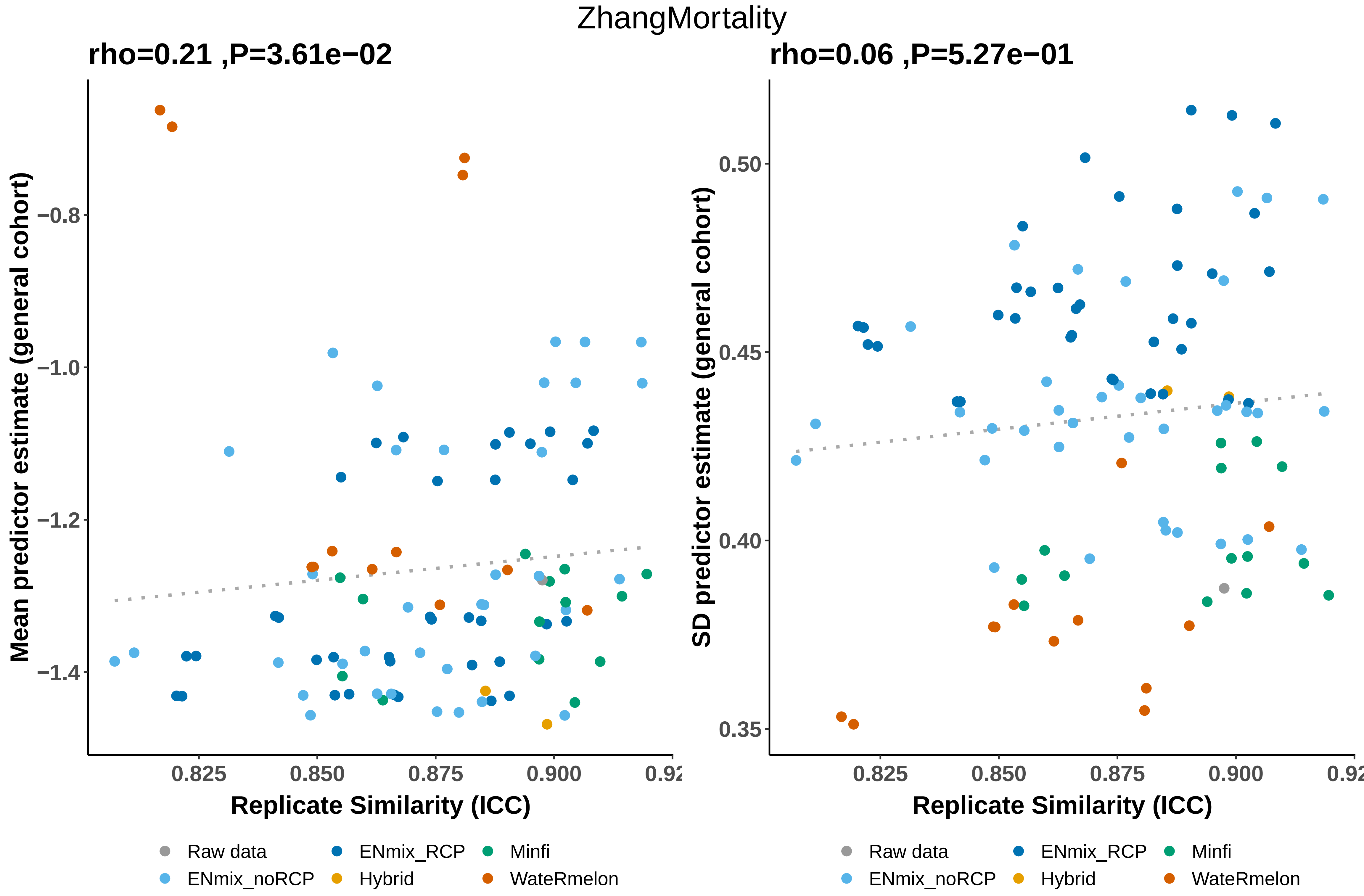
ZhangMortality
rho=0.21 ,P=3.61e−02
rho=0.06 ,P=5.27e−01
Mean predictor estimate (general cohort)
SD predictor estimate (general cohort)
−0.8
−1.0
−1.2
−1.4
0.50
0.45
0.40
0.35
0.825
0.850
0.875
0.900
Replicate Similarity (ICC)
Raw data
ENmix_RCP
Minfi
ENmix_noRCP
Hybrid
WateRmelon

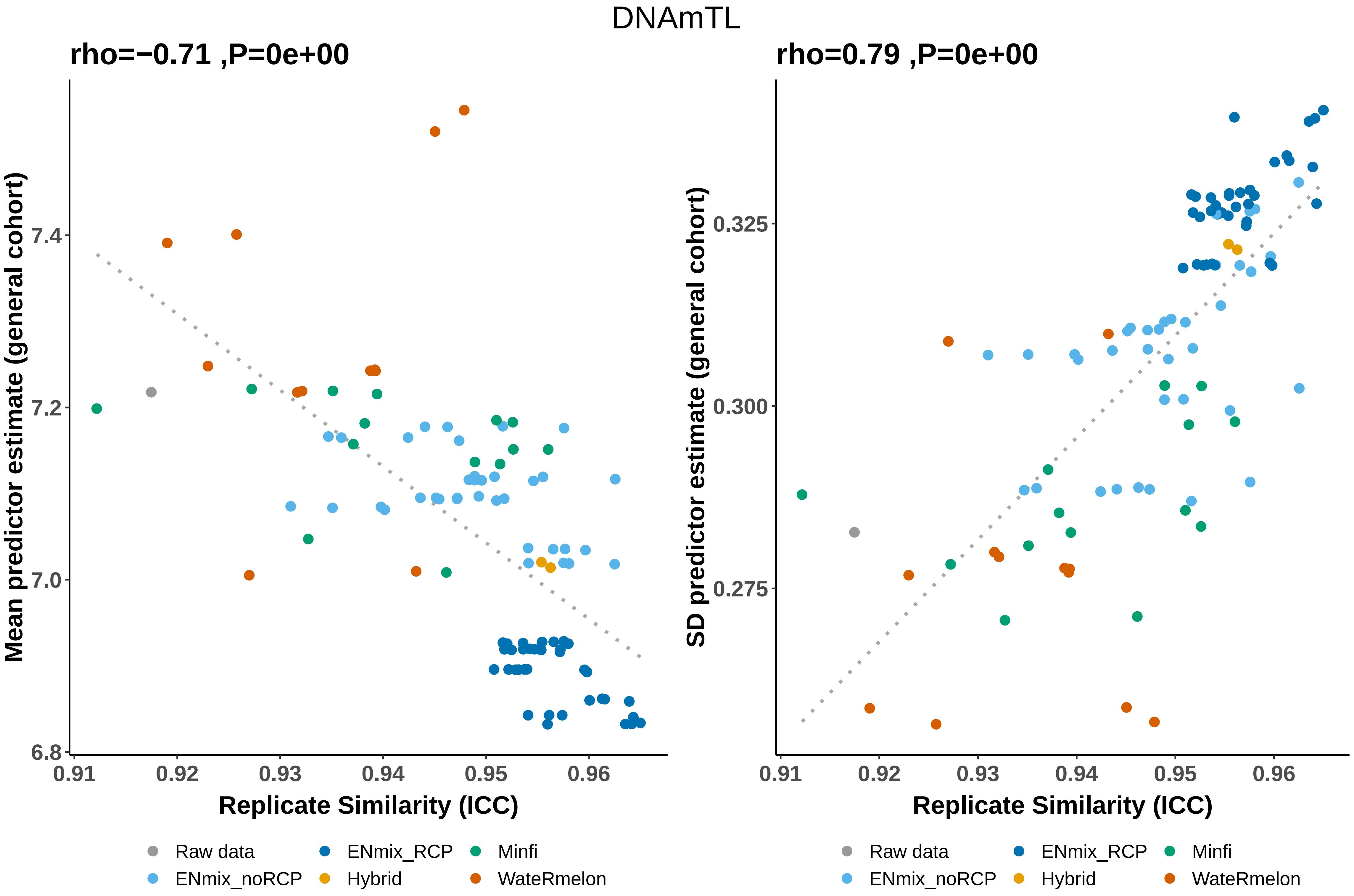

DNAmTL
rho=−0.71 ,P=0e+00
Mean predictor estimate (general cohort)
7.4
7.2
7.0
6.8
0.91
0.92
0.93
0.94
0.95
0.96
Replicate Similarity (ICC)
rho=0.79 ,P=0e+00
SD predictor estimate (general cohort)
0.325
0.300
0.275
Raw data
ENmix_RCP
Minfi
ENmix_noRCP
Hybrid
WateRmelon

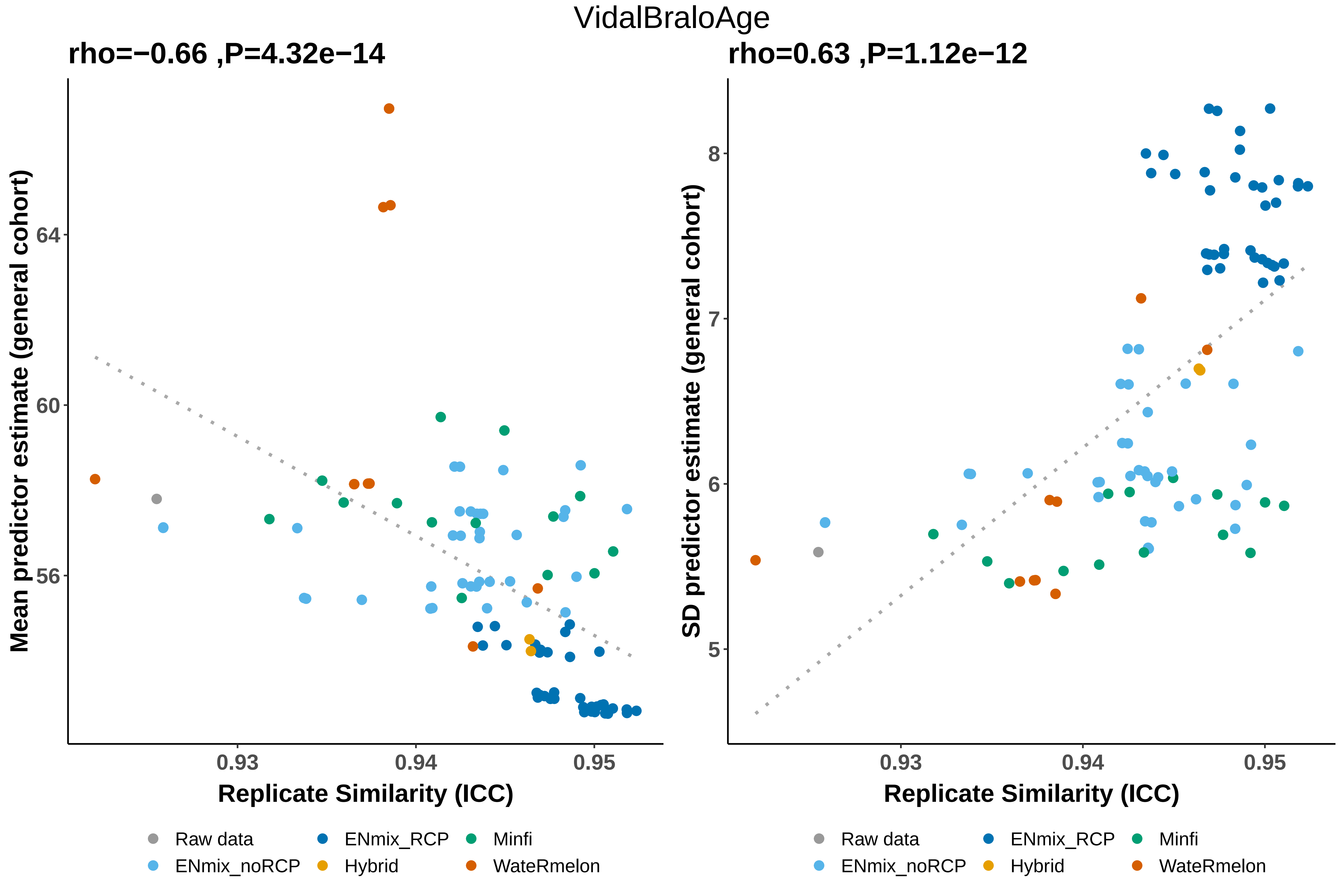
VidalBraloAge
rho=−0.66 ,P=4.32e−14
rho=0.63 ,P=1.12e−12
Mean predictor estimate (general cohort)
SD predictor estimate (general cohort)
64
60
56
8
7
6
5
0.93
0.94
0.95
Replicate Similarity (ICC)
Raw data
ENmix_noRCP
ENmix_RCP
Hybrid
Minfi
WateRmelon

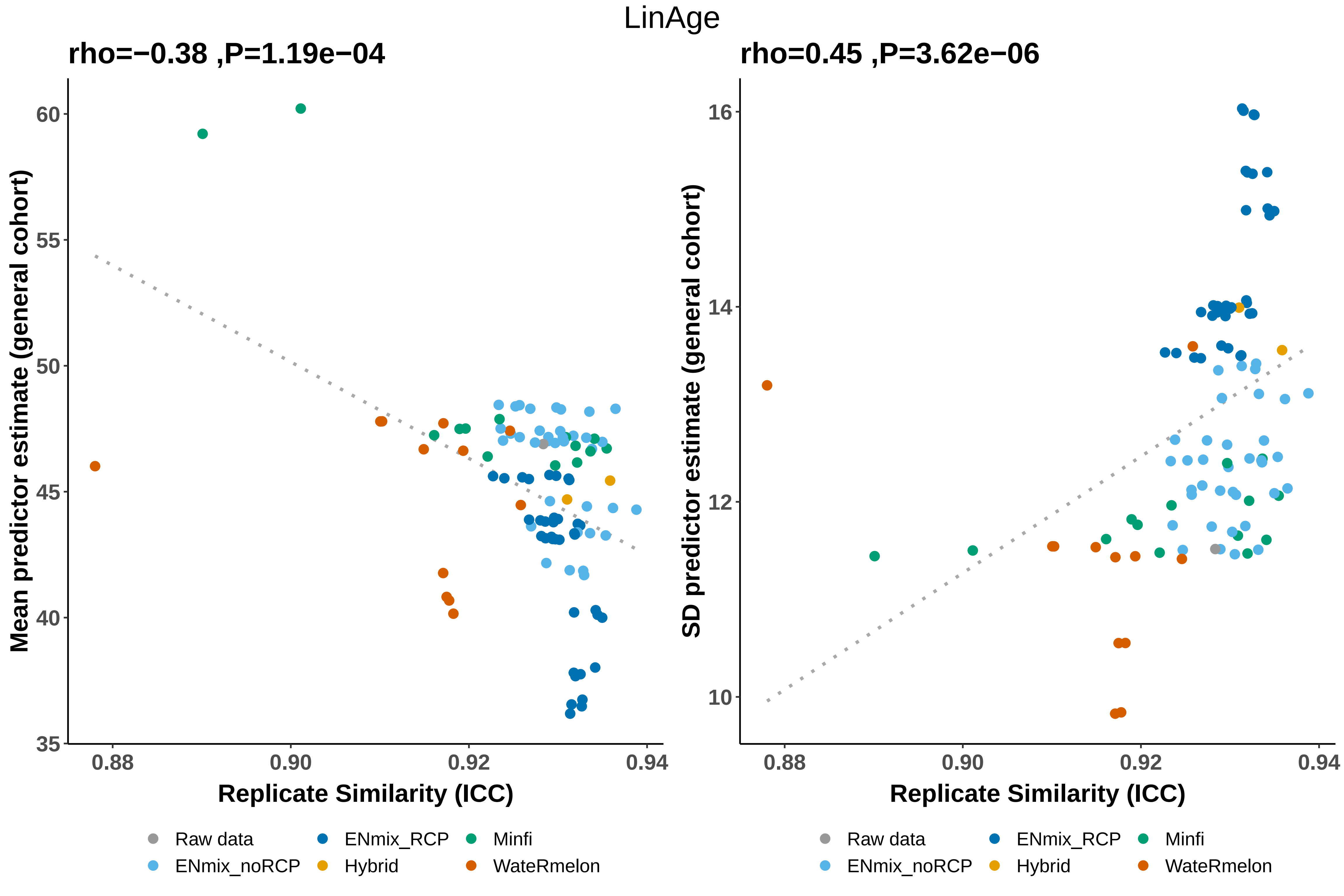
LinAge
rho=−0.38 ,P=1.19e−04
rho=0.45 ,P=3.62e−06
Mean predictor estimate (general cohort)
SD predictor estimate (general cohort)
Replicate Similarity (ICC)
Replicate Similarity (ICC)
0.88
0.90
0.92
0.94
35
40
45
50
55
60
10
12
14
16
Raw data
ENmix_RCP
Minfi
ENmix_noRCP
Hybrid
WateRmelon

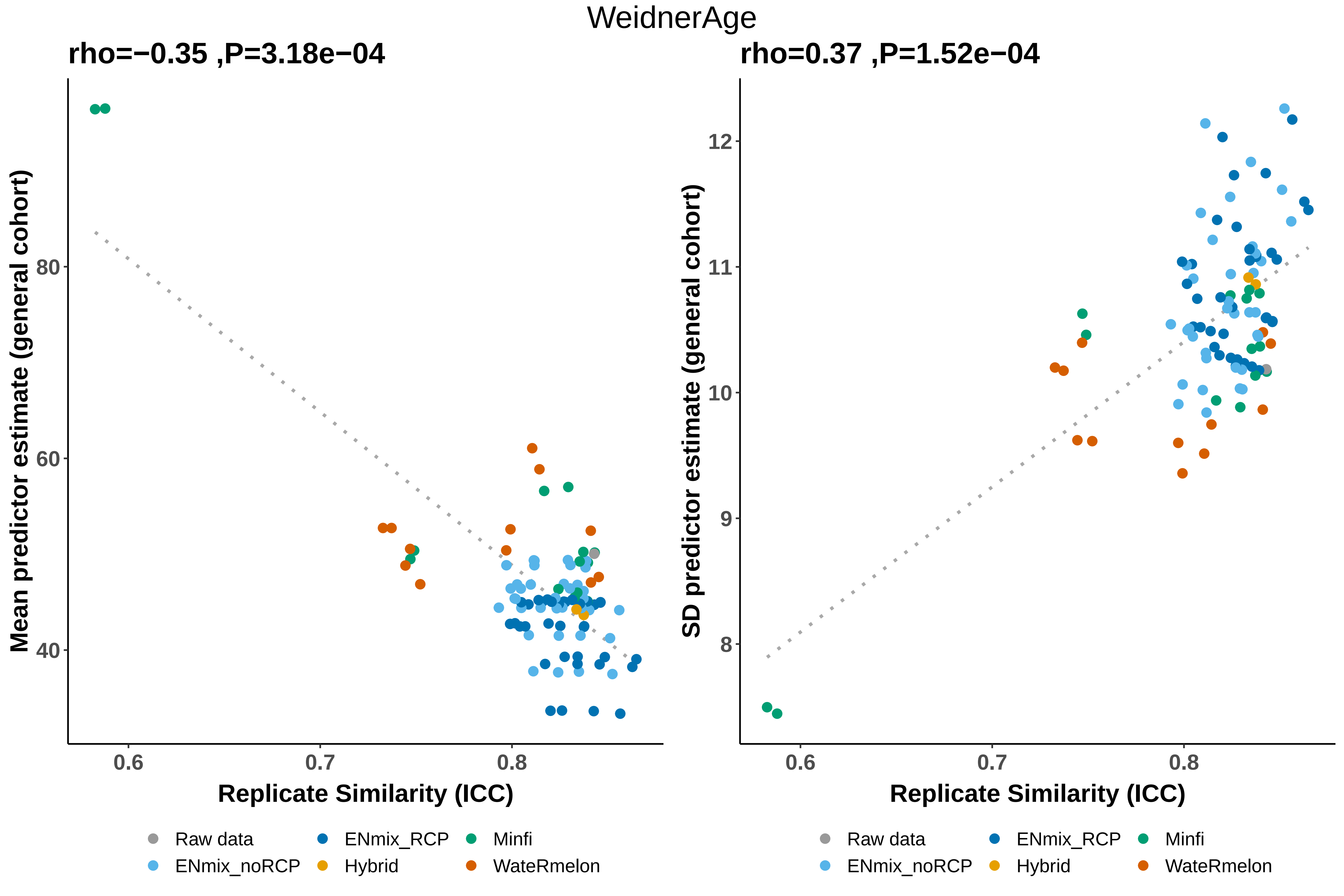

WeidnerAge
rho=−0.35 ,P=3.18e−04
rho=0.37 ,P=1.52e−04
Mean predictor estimate (general cohort)
SD predictor estimate (general cohort)
80
60
40
12
11
10
9
8
0.6
0.7
0.8
Replicate Similarity (ICC)
Raw data
ENmix_RCP
Minfi
ENmix_noRCP
Hybrid
WateRmelon

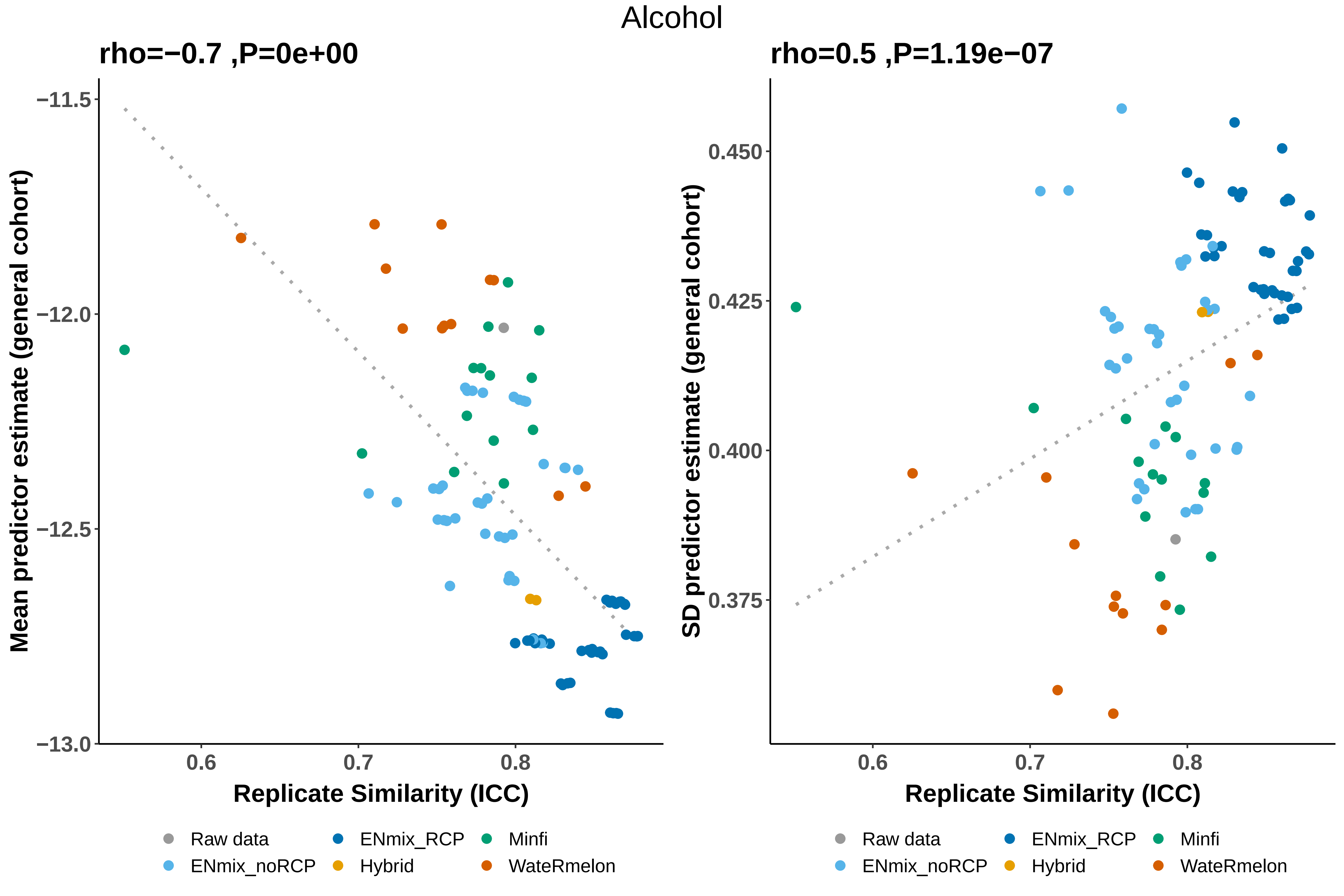

Alcohol
rho=−0.7 ,P=0e+00
rho=0.5 ,P=1.19e−07
Mean predictor estimate (general cohort)
SD predictor estimate (general cohort)
−11.5
−12.0
−12.5
−13.0
0.450
0.425
0.400
0.375
0.6
0.7
0.8
Replicate Similarity (ICC)
Raw data
ENmix_RCP
Minfi
ENmix_noRCP
Hybrid
WateRmelon

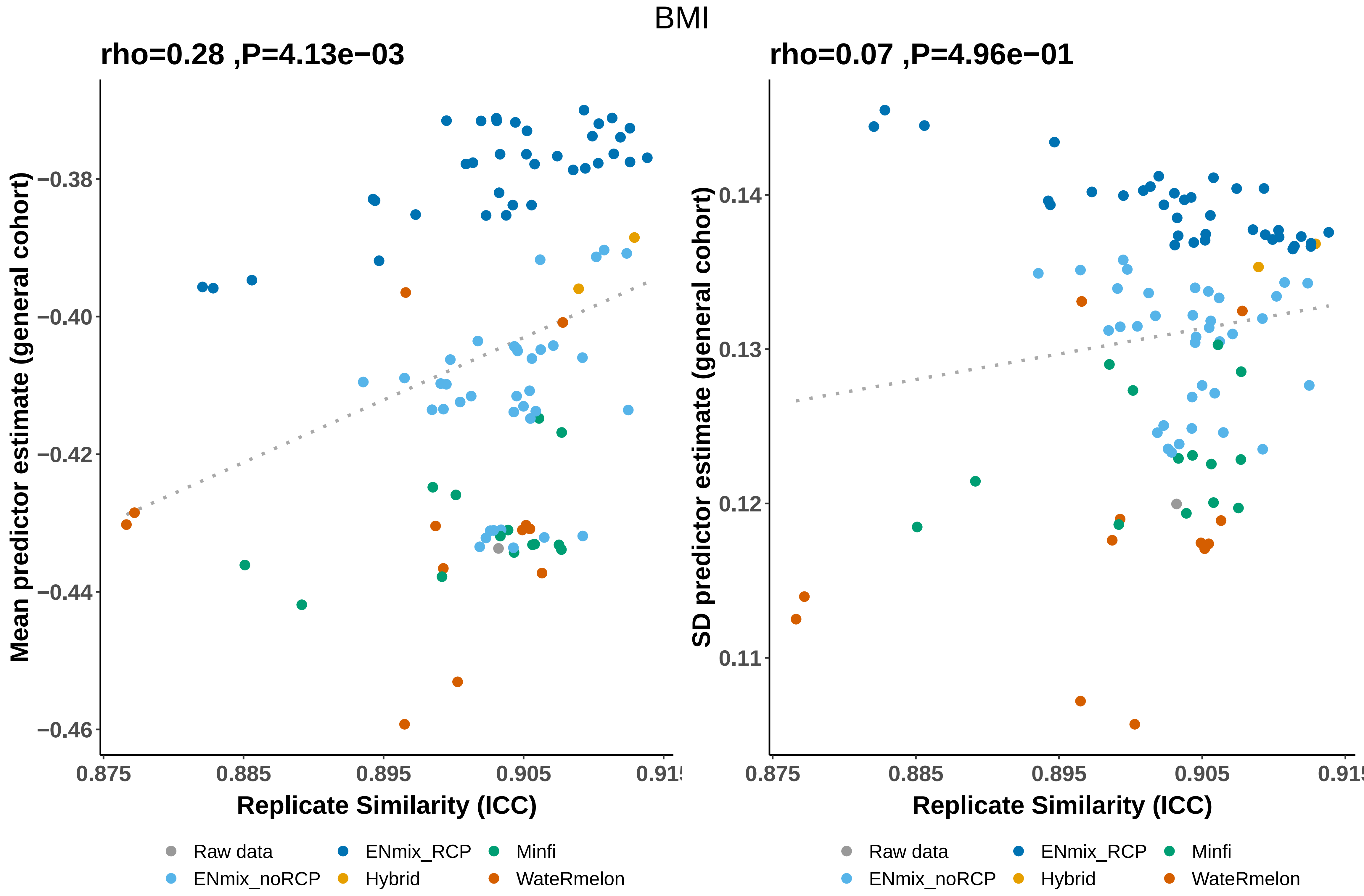

BMI
rho=0.28 ,P=4.13e−03
rho=0.07 ,P=4.96e−01
Mean predictor estimate (general cohort)
SD predictor estimate (general cohort)
−0.38
−0.40
−0.42
−0.44
−0.46
0.14
0.13
0.12
0.11
0.875
0.885
0.895
0.905
0.915
Replicate Similarity (ICC)
Raw data
ENmix_RCP
Minfi
ENmix_noRCP
Hybrid
WateRmelon

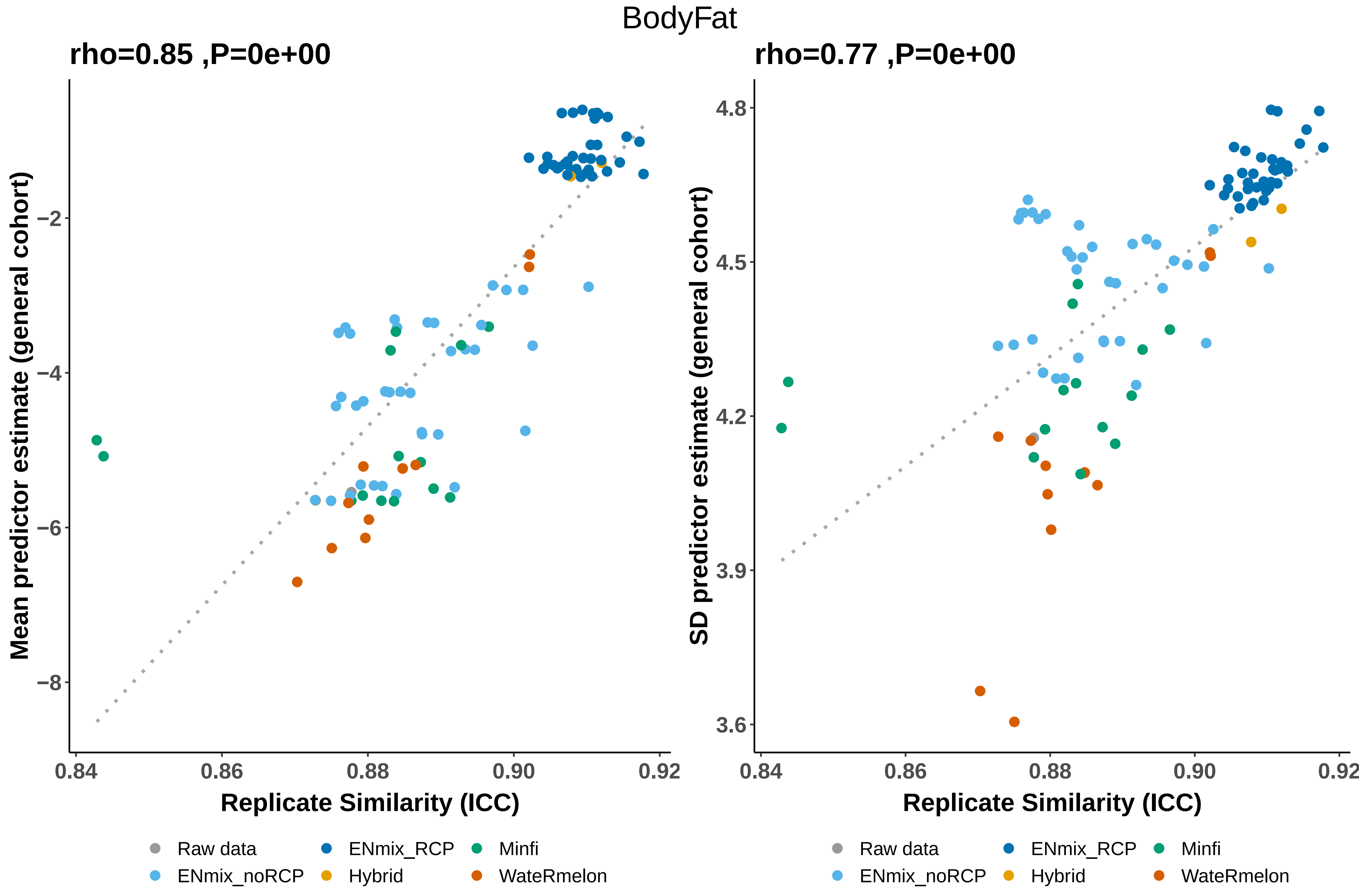

BodyFat
rho=0.85 ,P=0e+00
rho=0.77 ,P=0e+00
Mean predictor estimate (general cohort)
SD predictor estimate (general cohort)
−2
−4
−6
−8
4.8
4.5
4.2
3.9
3.6
0.84
0.86
0.88
0.90
0.92
Replicate Similarity (ICC)
Replicate Similarity (ICC)
Raw data
ENmix_noRCP
ENmix_RCP
Hybrid
Minfi
WateRmelon

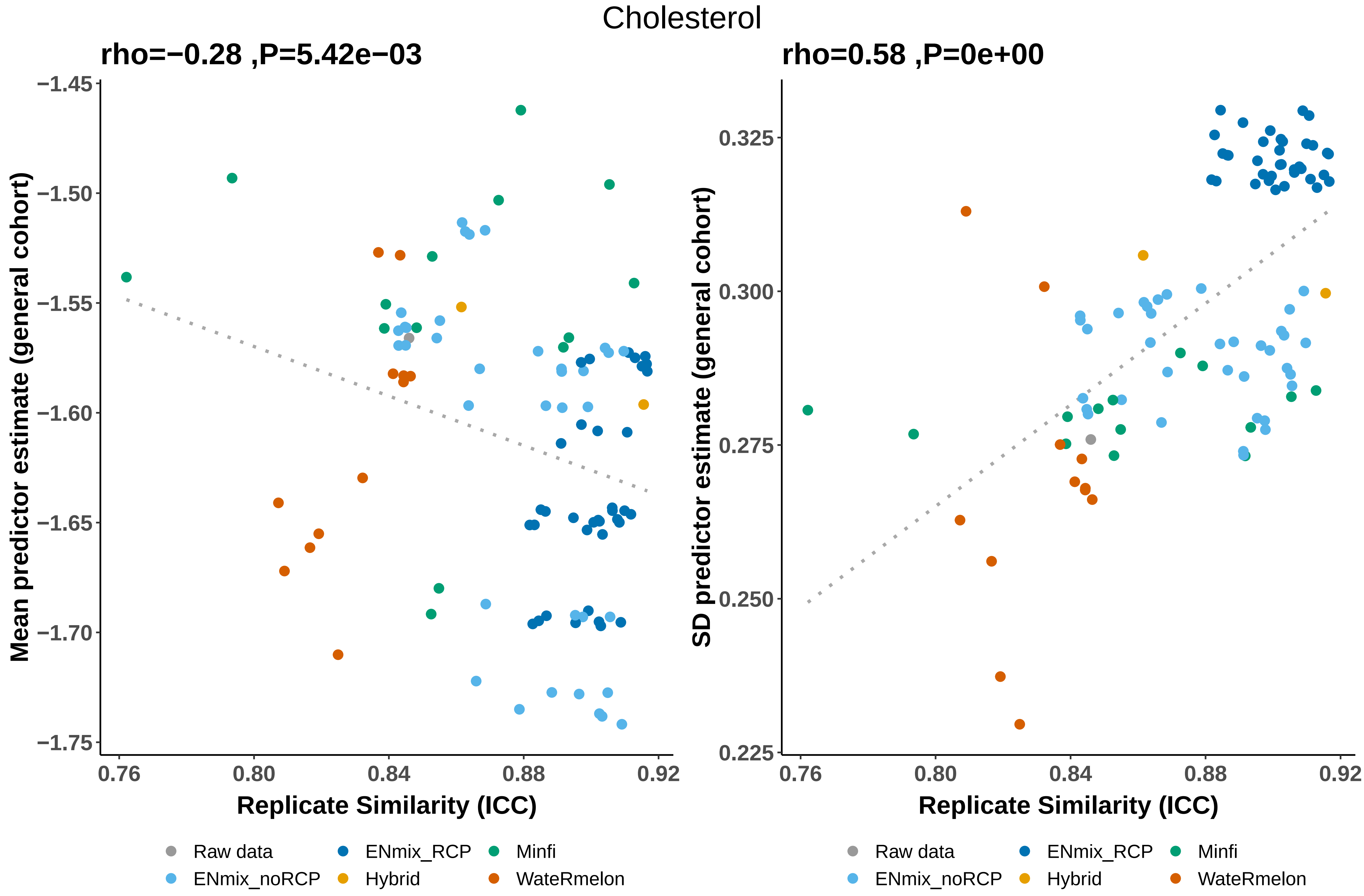

Cholesterol
rho=−0.28 ,P=5.42e−03
rho=0.58 ,P=0e+00
Mean predictor estimate (general cohort)
SD predictor estimate (general cohort)
Replicate Similarity (ICC)
Replicate Similarity (ICC)
−1.45
−1.50
−1.55
−1.60
−1.65
−1.70
−1.75
0.325
0.300
0.275
0.250
0.225
0.76
0.80
0.84
0.88
0.92
Raw data
ENmix_noRCP
ENmix_RCP
Hybrid
Minfi
WateRmelon

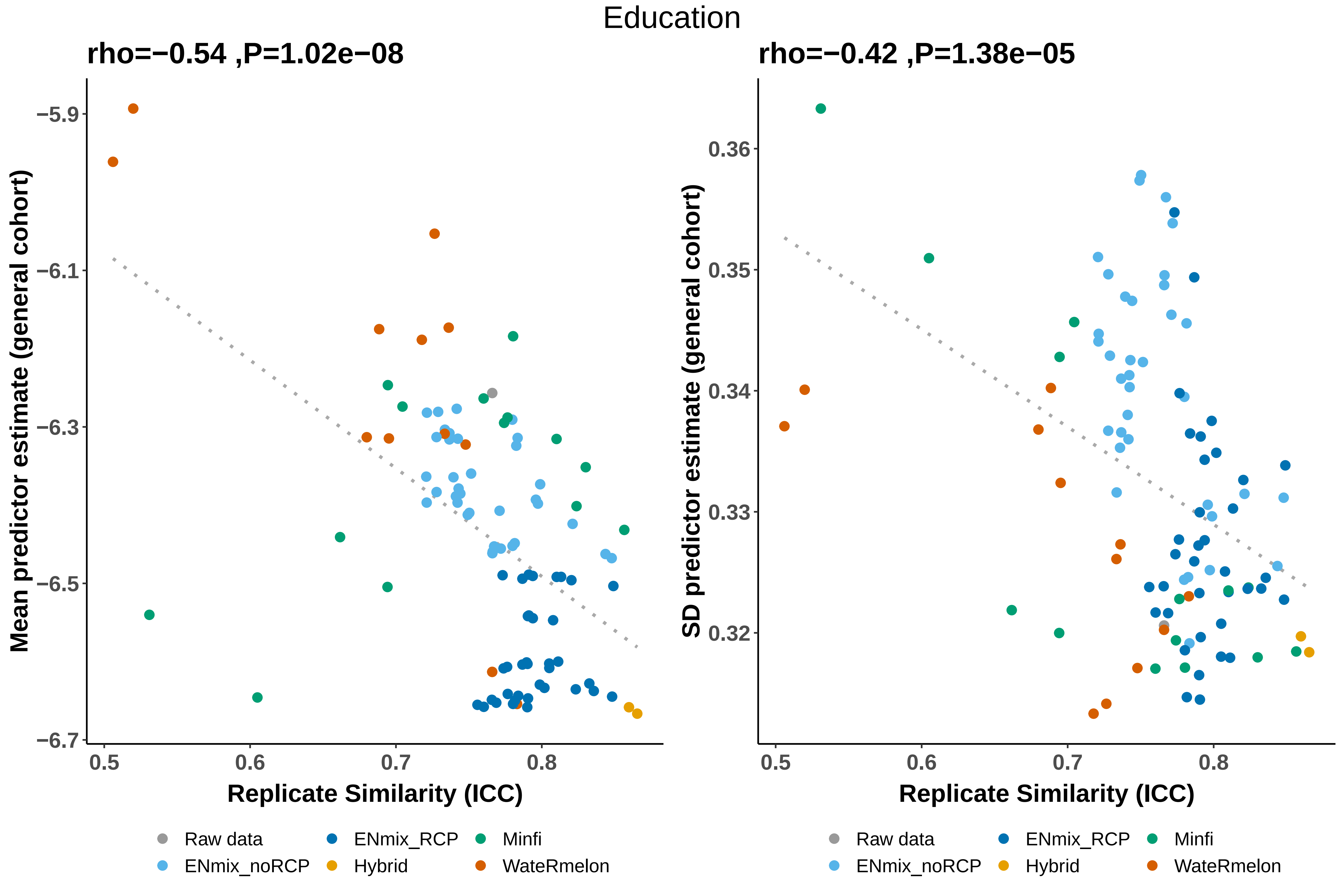
Education
rho=−0.54 ,P=1.02e−08
rho=−0.42 ,P=1.38e−05
Mean predictor estimate (general cohort)
SD predictor estimate (general cohort)
−5.9
−6.1
−6.3
−6.5
−6.7
0.36
0.35
0.34
0.33
0.32
0.5
0.6
0.7
0.8
Replicate Similarity (ICC)
Raw data
ENmix_RCP
Minfi
ENmix_noRCP
Hybrid
WateRmelon

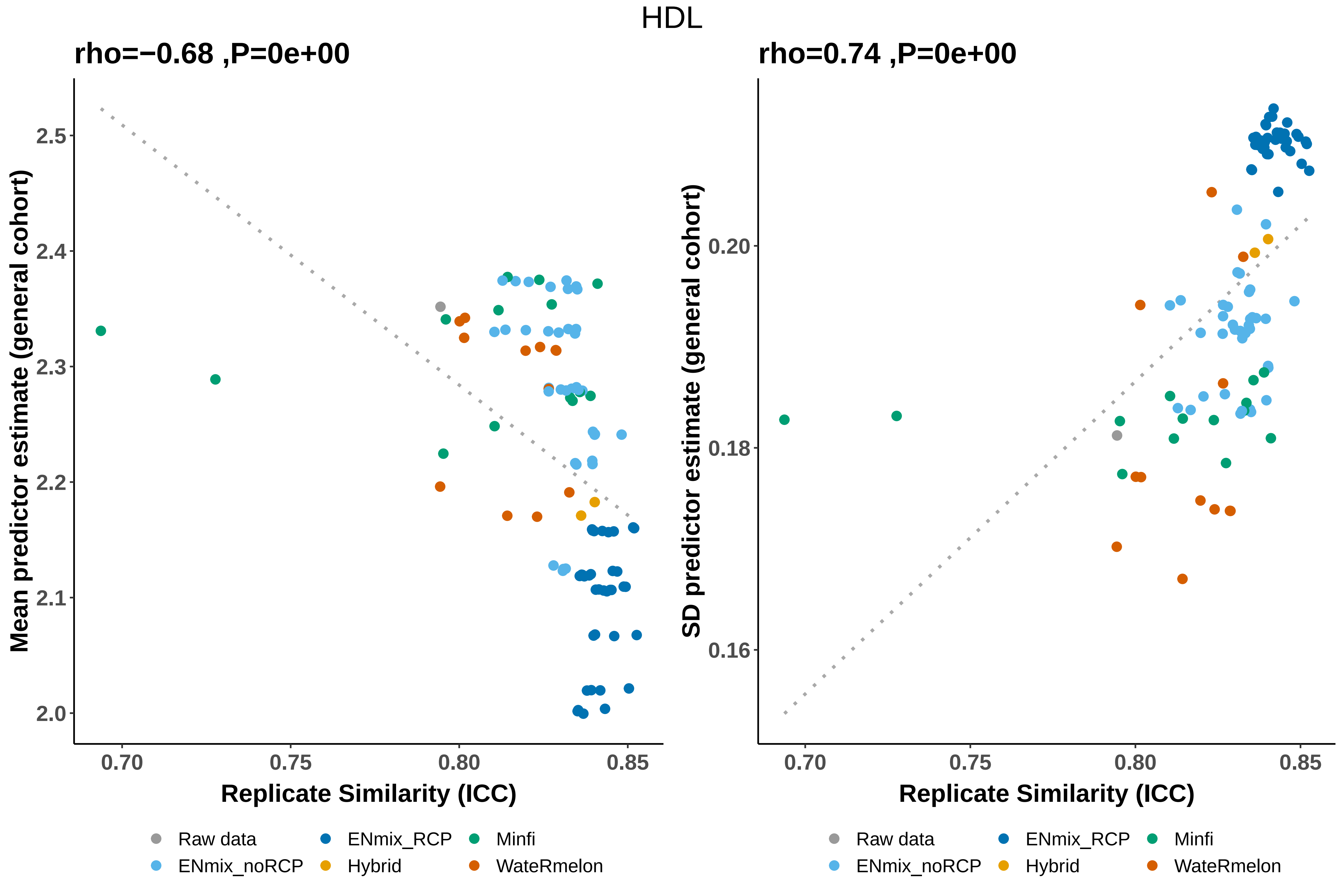

HDL
rho=−0.68 ,P=0e+00
rho=0.74 ,P=0e+00
Mean predictor estimate (general cohort)
SD predictor estimate (general cohort)
2.5
2.4
2.3
2.2
2.1
2.0
0.20
0.18
0.16
0.70
0.75
0.80
0.85
Replicate Similarity (ICC)
Raw data
ENmix_noRCP
ENmix_RCP
Hybrid
Minfi
WateRmelon

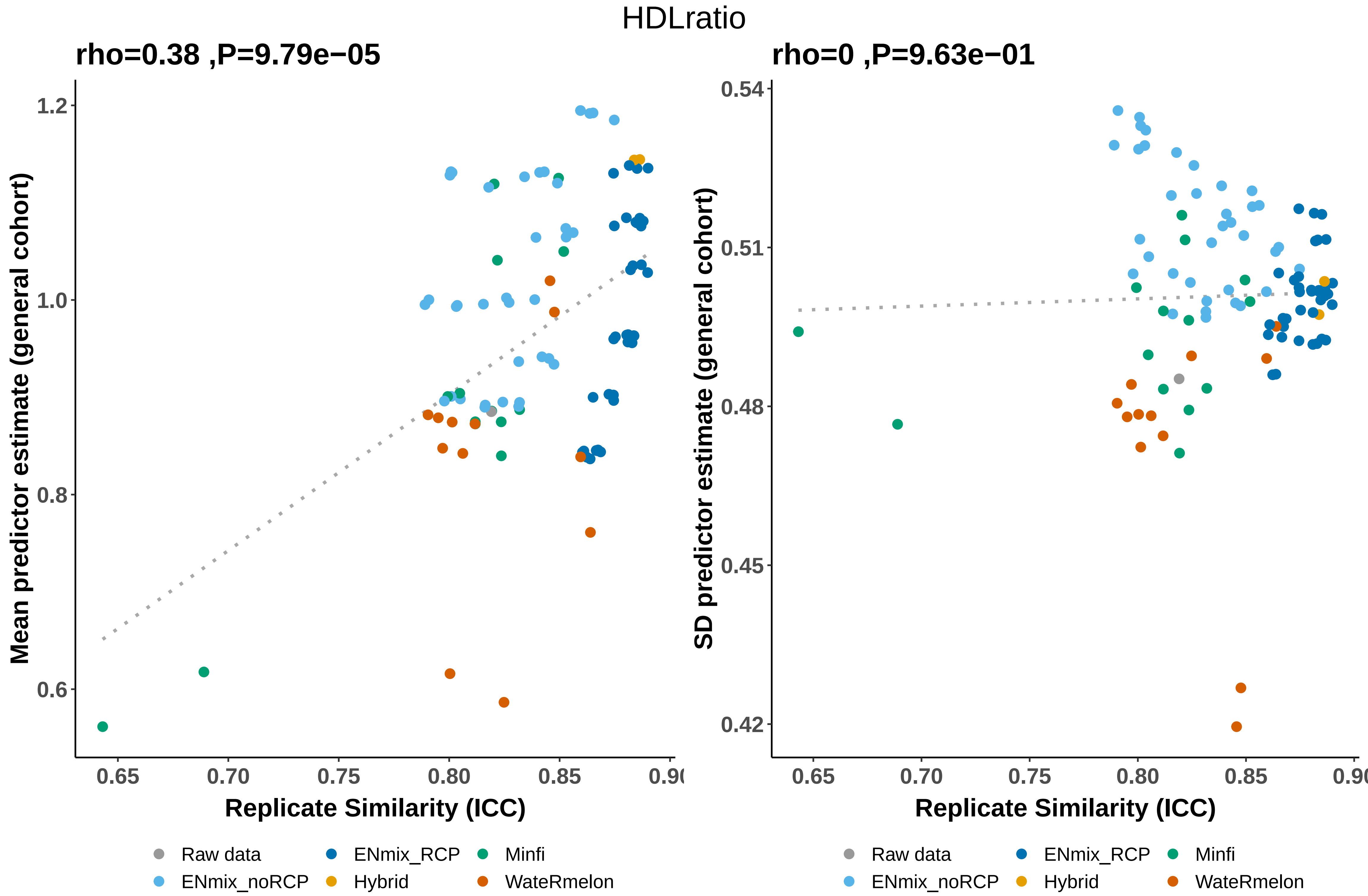
HDLratio
rho=0.38 ,P=9.79e−05
Mean predictor estimate (general cohort)
Replicate Similarity (ICC)
rho=0 ,P=9.63e−01
SD predictor estimate (general cohort)
Replicate Similarity (ICC)
Raw data
ENmix_noRCP
ENmix_RCP
Hybrid
Minfi
WateRmelon

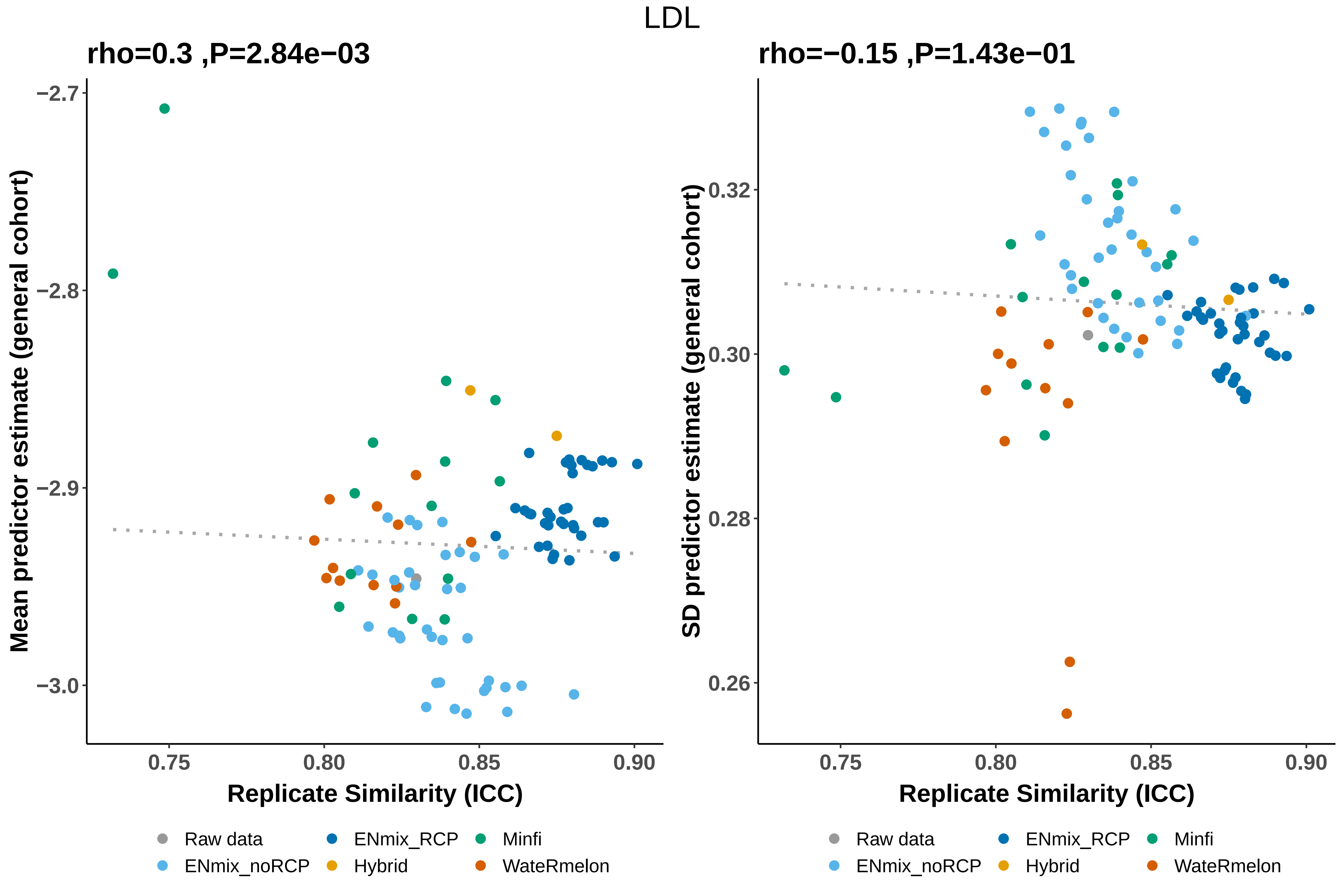

LDL
rho=0.3 ,P=2.84e−03
rho=−0.15 ,P=1.43e−01
Mean predictor estimate (general cohort)
SD predictor estimate (general cohort)
−2.7
−2.8
−2.9
−3.0
0.32
0.30
0.28
0.26
0.75
0.80
0.85
0.90
Replicate Similarity (ICC)
Replicate Similarity (ICC)
Raw data
ENmix_RCP
Minfi
ENmix_noRCP
Hybrid
WateRmelon

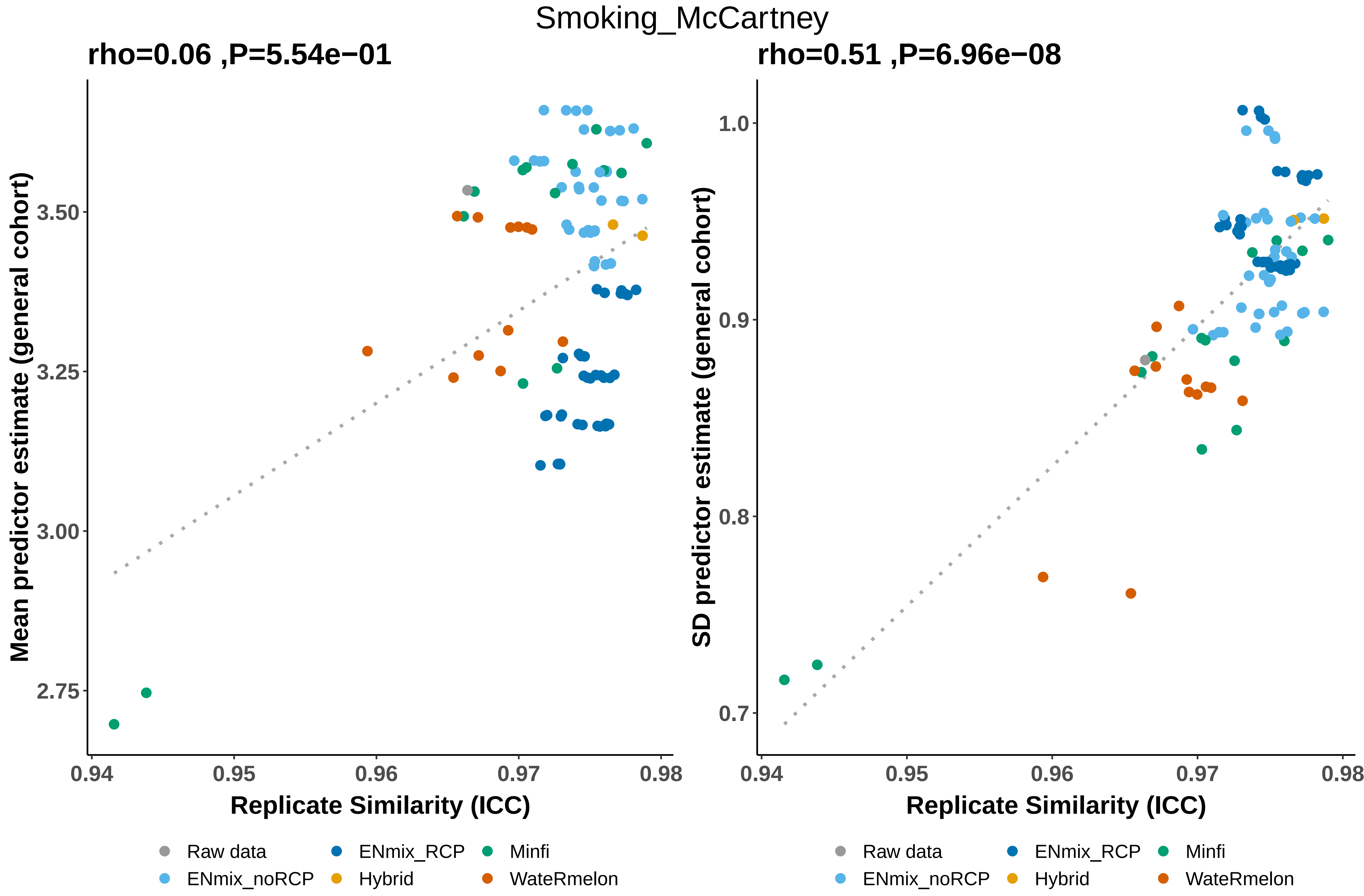

Smoking_McCartney
rho=0.06 ,P=5.54e−01
rho=0.51 ,P=6.96e−08
Mean predictor estimate (general cohort)
SD predictor estimate (general cohort)
3.50
3.25
3.00
2.75
1.0
0.9
0.8
0.7
0.94
0.95
0.96
0.97
0.98
Replicate Similarity (ICC)
Raw data
ENmix_RCP
Minfi
ENmix_noRCP
Hybrid
WateRmelon

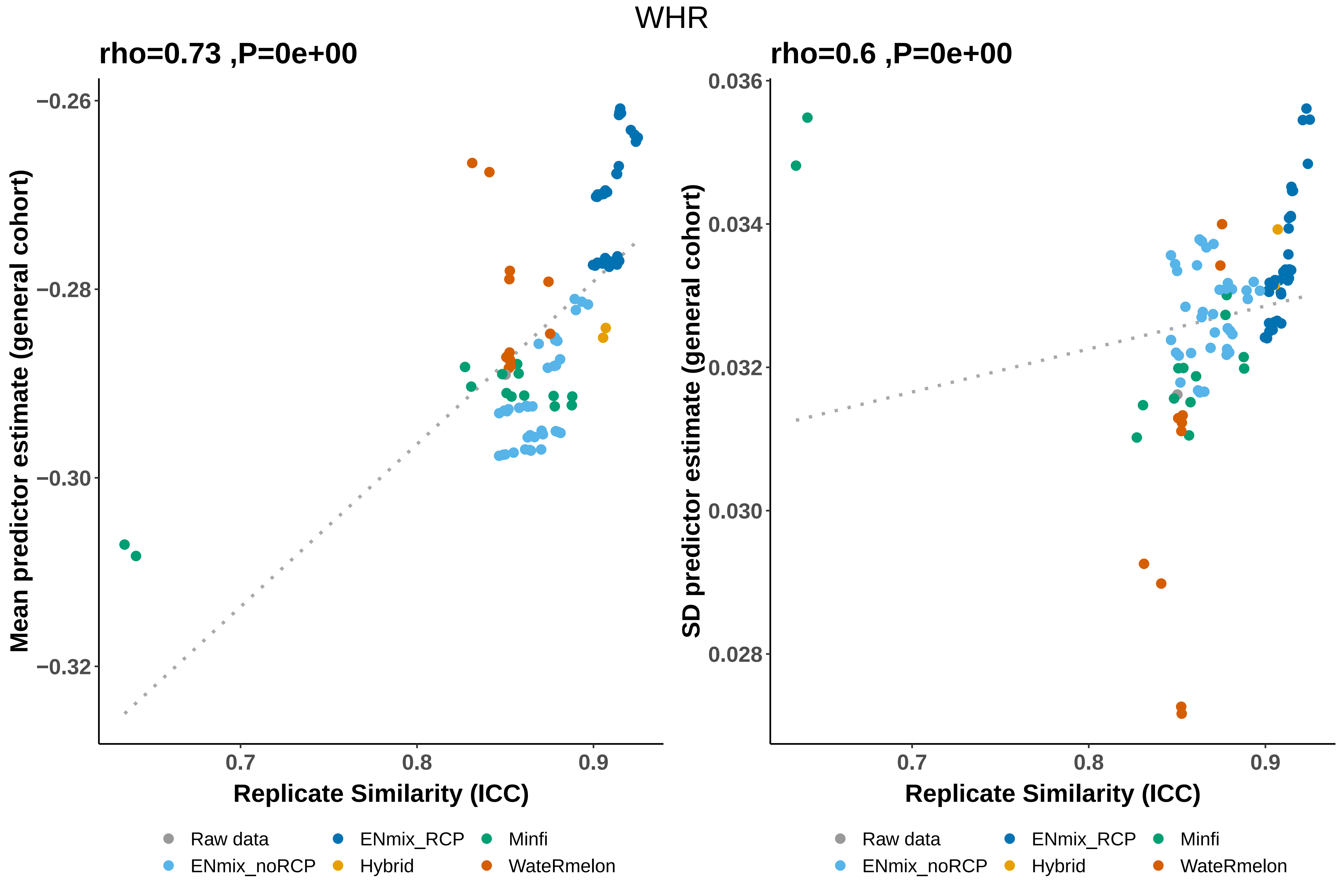
WHR
rho=0.73 ,P=0e+00
rho=0.6 ,P=0e+00
Mean predictor estimate (general cohort)
SD predictor estimate (general cohort)
−0.26
−0.28
−0.30
−0.32
0.036
0.034
0.032
0.030
0.028
0.7
0.8
0.9
Replicate Similarity (ICC)
Raw data
ENmix_noRCP
ENmix_RCP
Hybrid
Minfi
WateRmelon

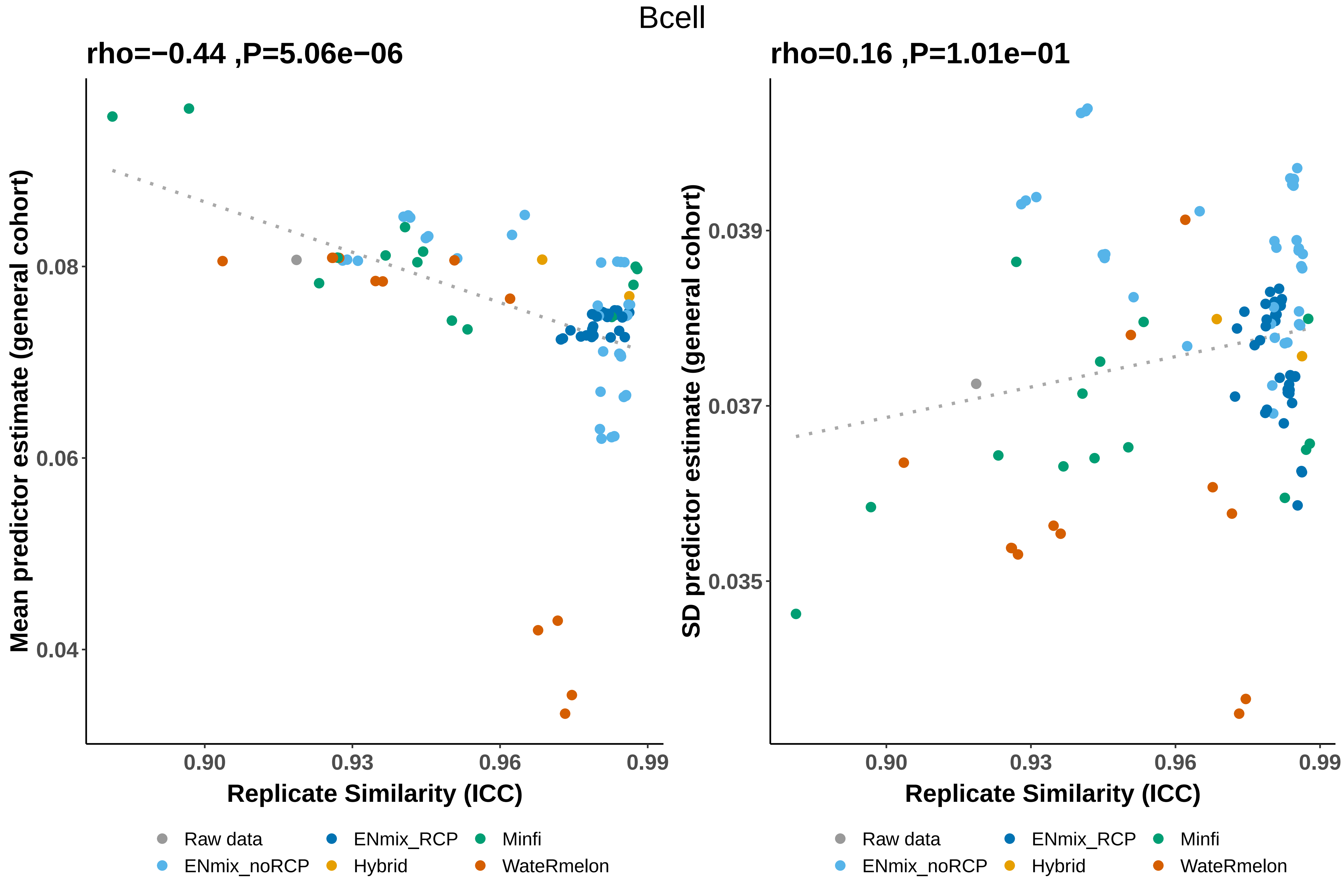

Bcell
rho=−0.44 ,P=5.06e−06
rho=0.16 ,P=1.01e−01
Mean predictor estimate (general cohort)
SD predictor estimate (general cohort)
0.08
0.06
0.04
0.039
0.037
0.035
0.90
0.93
0.96
0.99
Replicate Similarity (ICC)
Raw data
ENmix_noRCP
ENmix_RCP
Hybrid
Minfi
WateRmelon

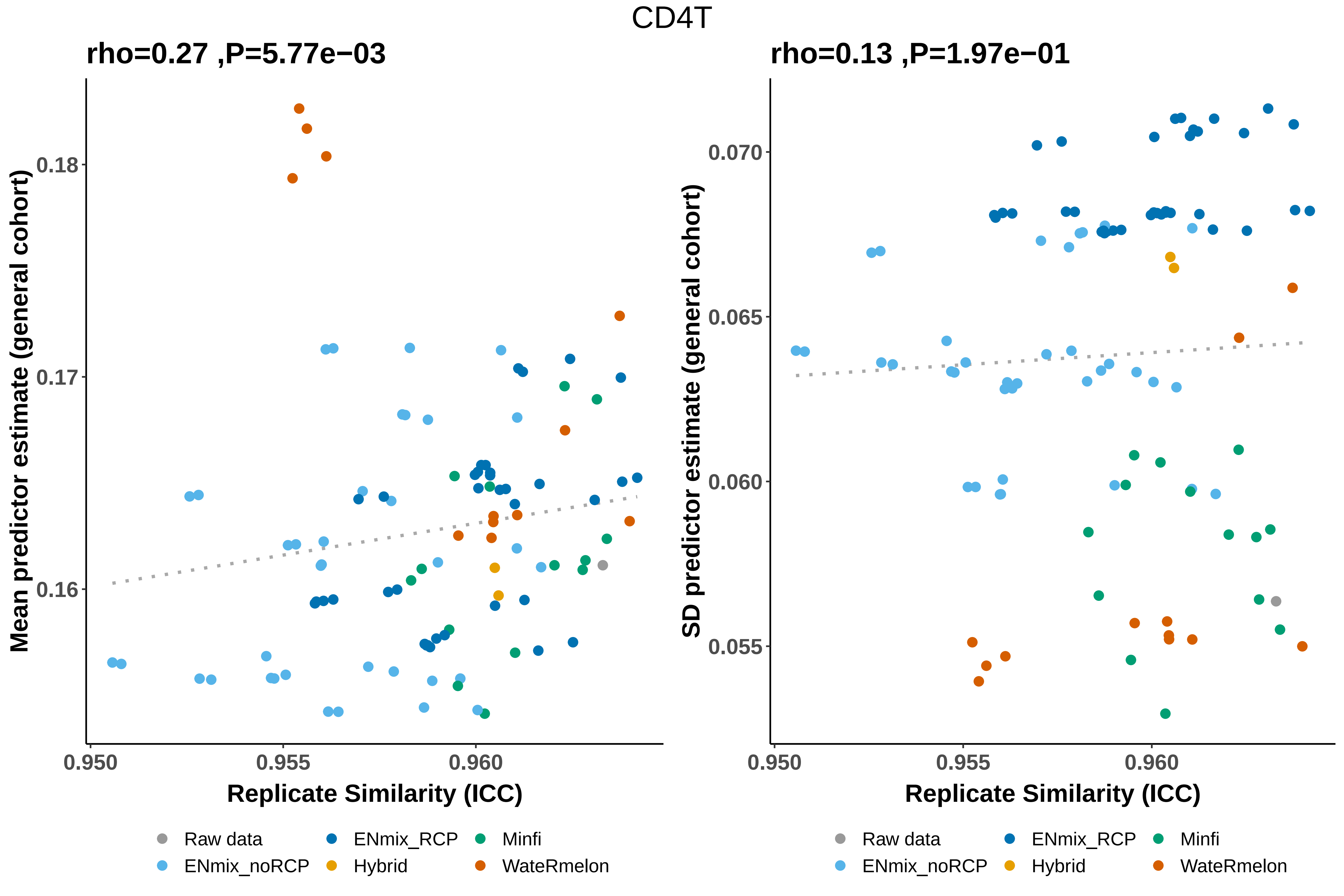
CD4T
rho=0.27 ,P=5.77e−03
rho=0.13 ,P=1.97e−01
Mean predictor estimate (general cohort)
SD predictor estimate (general cohort)
0.18
0.17
0.16
0.070
0.065
0.060
0.055
0.950
0.955
0.960
Replicate Similarity (ICC)
Raw data
ENmix_noRCP
ENmix_RCP
Hybrid
Minfi
WateRmelon

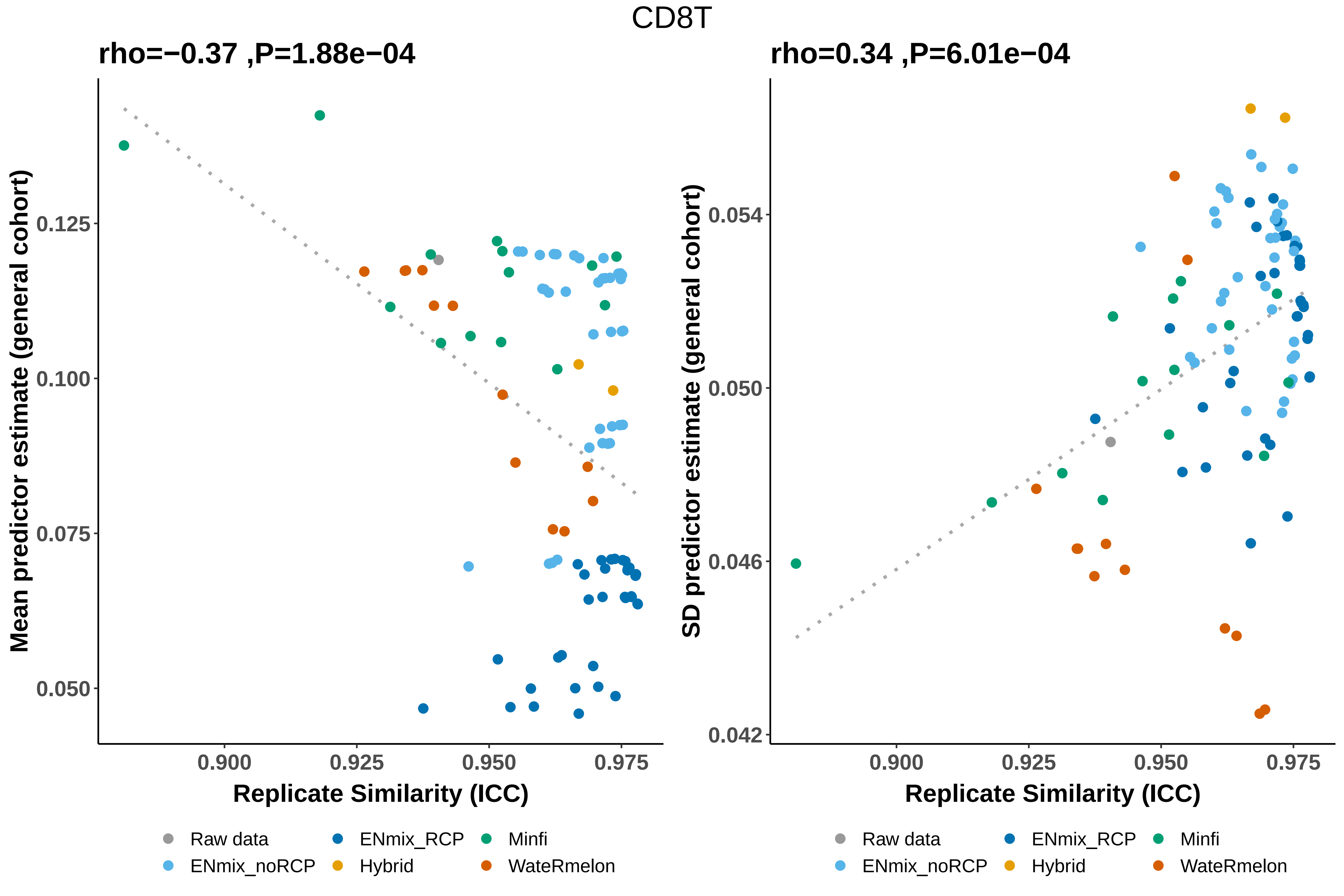

CD8T
rho=−0.37 ,P=1.88e−04
Mean predictor estimate (general cohort)
0.125
0.100
0.075
0.050
0.900
0.925
0.950
0.975
Replicate Similarity (ICC)
rho=0.34 ,P=6.01e−04
SD predictor estimate (general cohort)
0.054
0.050
0.046
0.042
0.900
0.925
0.950
0.975
Replicate Similarity (ICC)
Raw data
ENmix_noRCP
ENmix_RCP
Hybrid
Minfi
WateRmelon

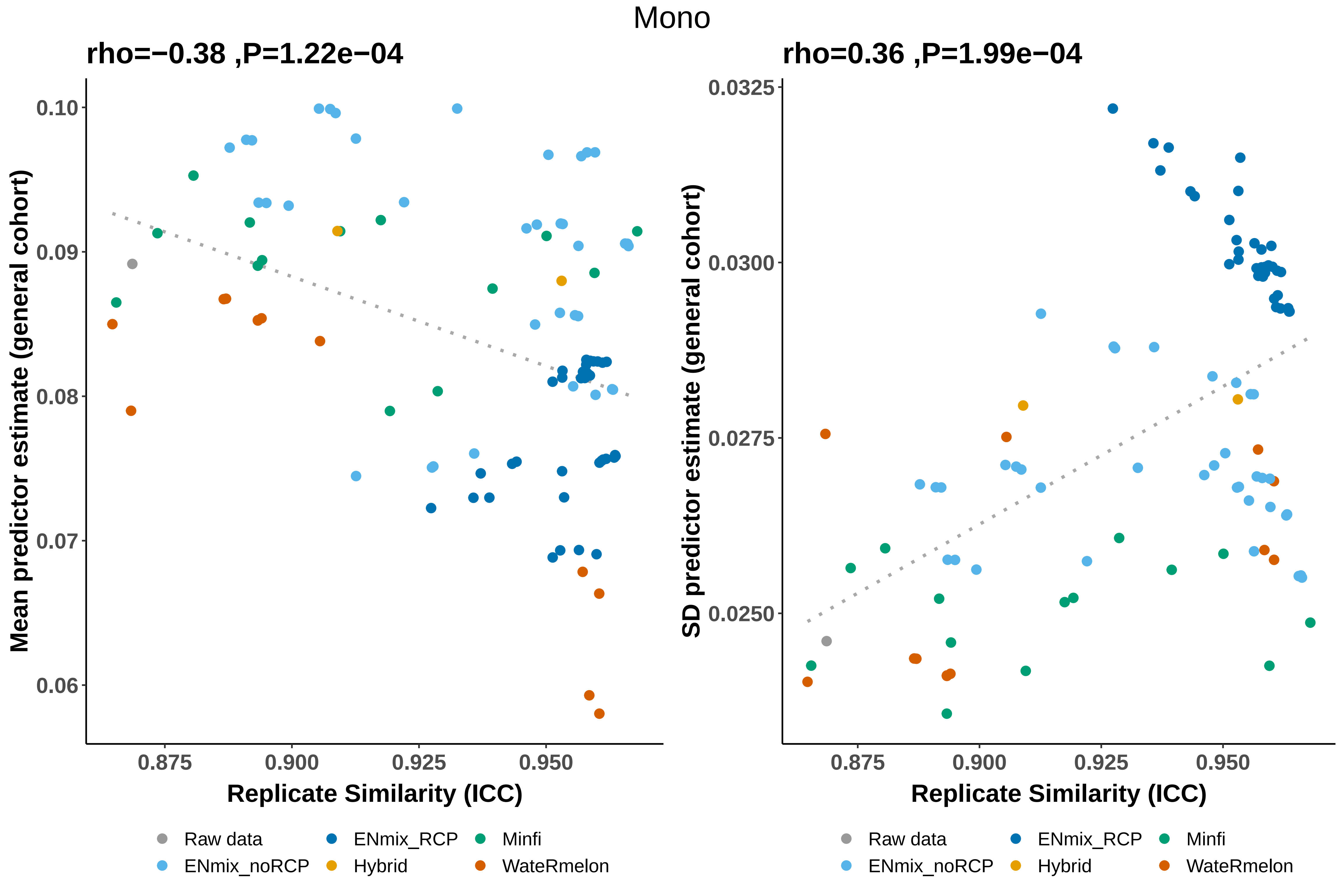

Mono
rho=−0.38 ,P=1.22e−04
rho=0.36 ,P=1.99e−04
Mean predictor estimate (general cohort)
SD predictor estimate (general cohort)
0.10
0.09
0.08
0.07
0.06
0.0325
0.0300
0.0275
0.0250
0.875
0.900
0.925
0.950
Replicate Similarity (ICC)
Raw data
ENmix_noRCP
ENmix_RCP
Hybrid
Minfi
WateRmelon

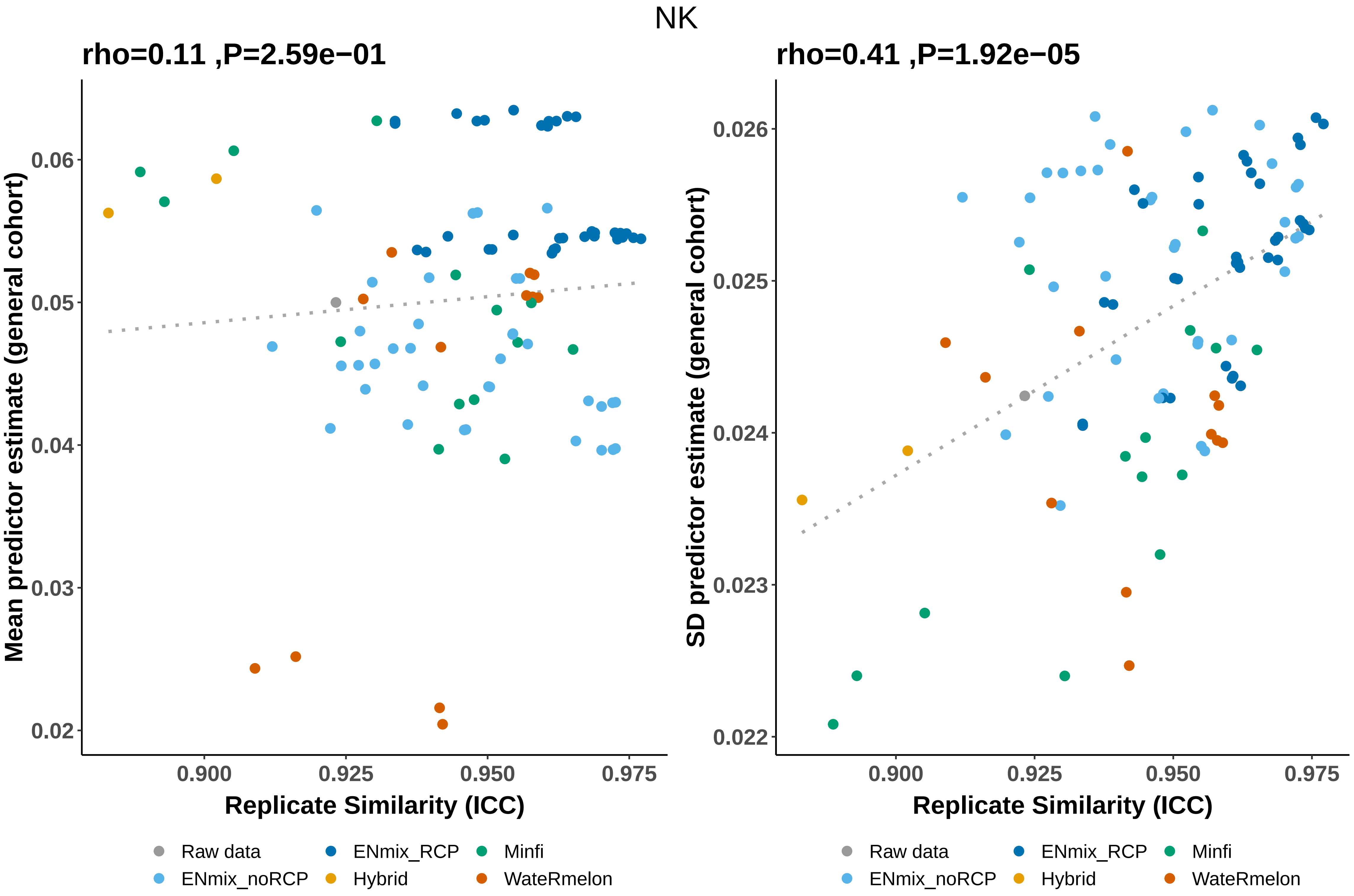

NK
rho=0.11 ,P=2.59e−01
rho=0.41 ,P=1.92e−05
Mean predictor estimate (general cohort)
SD predictor estimate (general cohort)
0.06
0.05
0.04
0.03
0.02
0.026
0.025
0.024
0.023
0.022
0.900
0.925
0.950
0.975
Replicate Similarity (ICC)
Raw data
ENmix_noRCP
ENmix_RCP
Hybrid
Minfi
WateRmelon

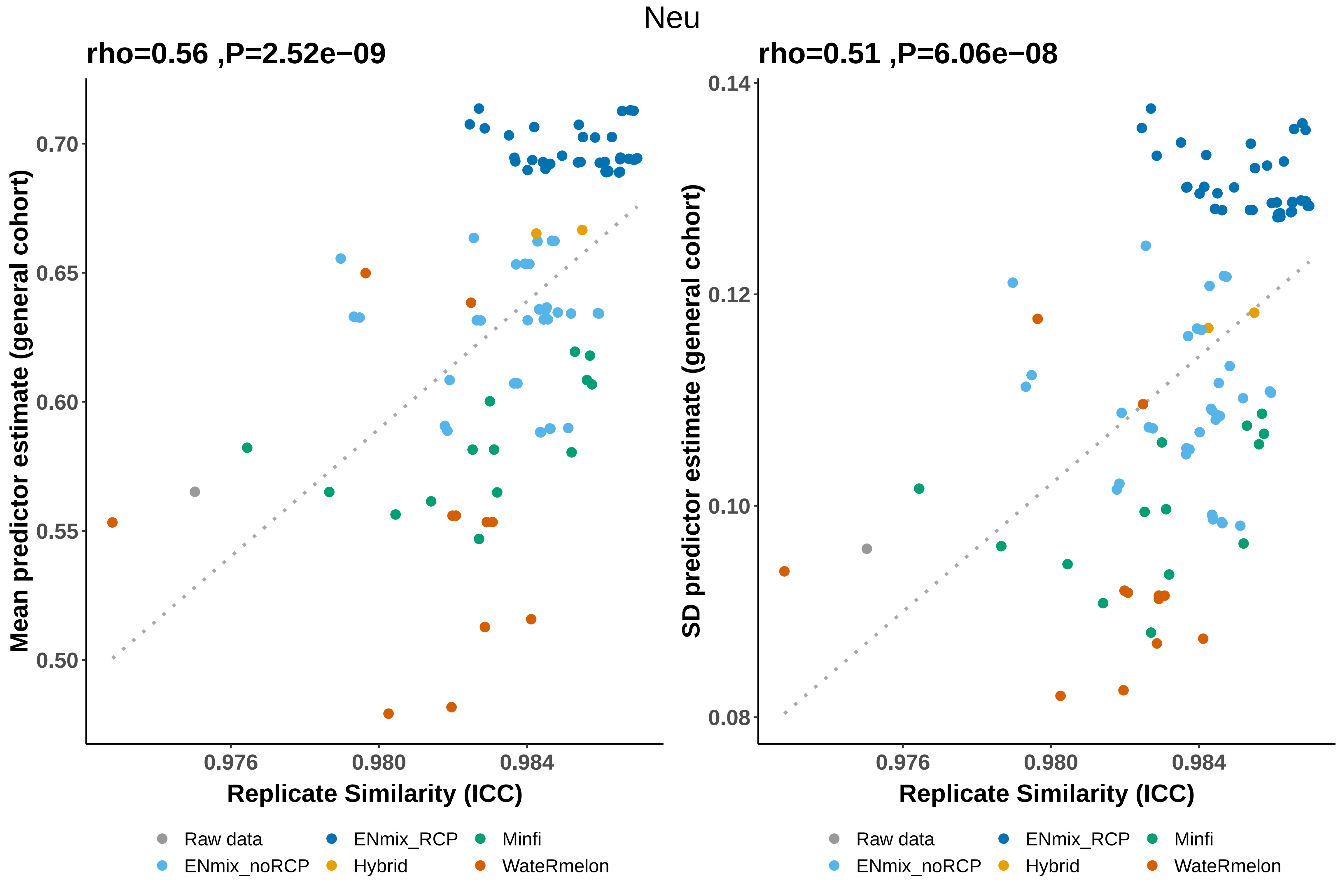

Neu
rho=0.56 ,P=2.52e−09
rho=0.51 ,P=6.06e−08
Mean predictor estimate (general cohort)
SD predictor estimate (general cohort)
0.70
0.65
0.60
0.55
0.50
0.14
0.12
0.10
0.08
0.976
0.980
0.984
Replicate Similarity (ICC)
Raw data
ENmix_noRCP
ENmix_RCP
Hybrid
Minfi
WateRmelon

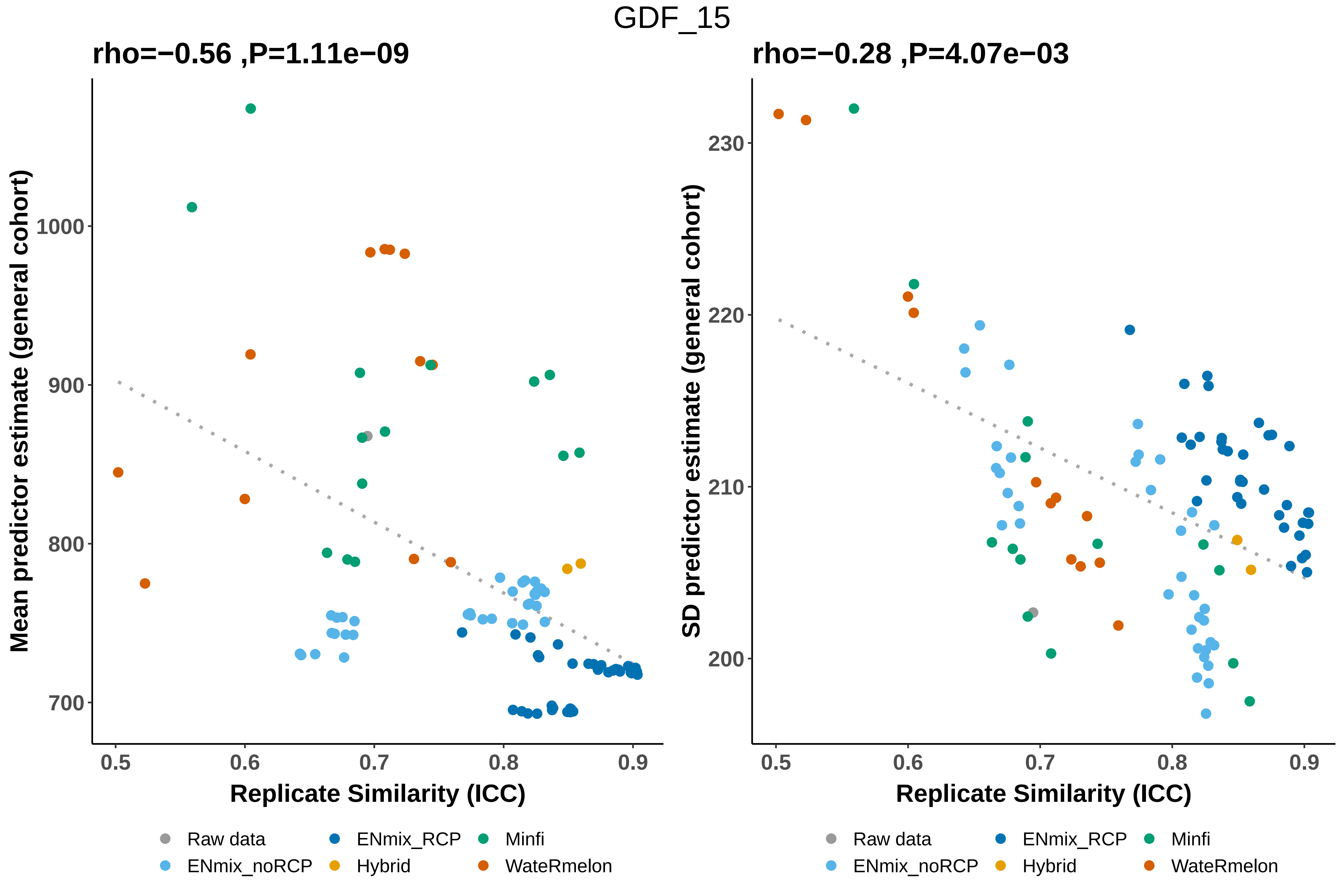
GDF_15
rho=−0.56 ,P=1.11e−09
rho=−0.28 ,P=4.07e−03
Mean predictor estimate (general cohort)
SD predictor estimate (general cohort)
Replicate Similarity (ICC)
Replicate Similarity (ICC)
1000
900
800
700
230
220
210
200
0.5
0.6
0.7
0.8
0.9
Raw data
ENmix_noRCP
ENmix_RCP
Hybrid
Minfi
WateRmelon

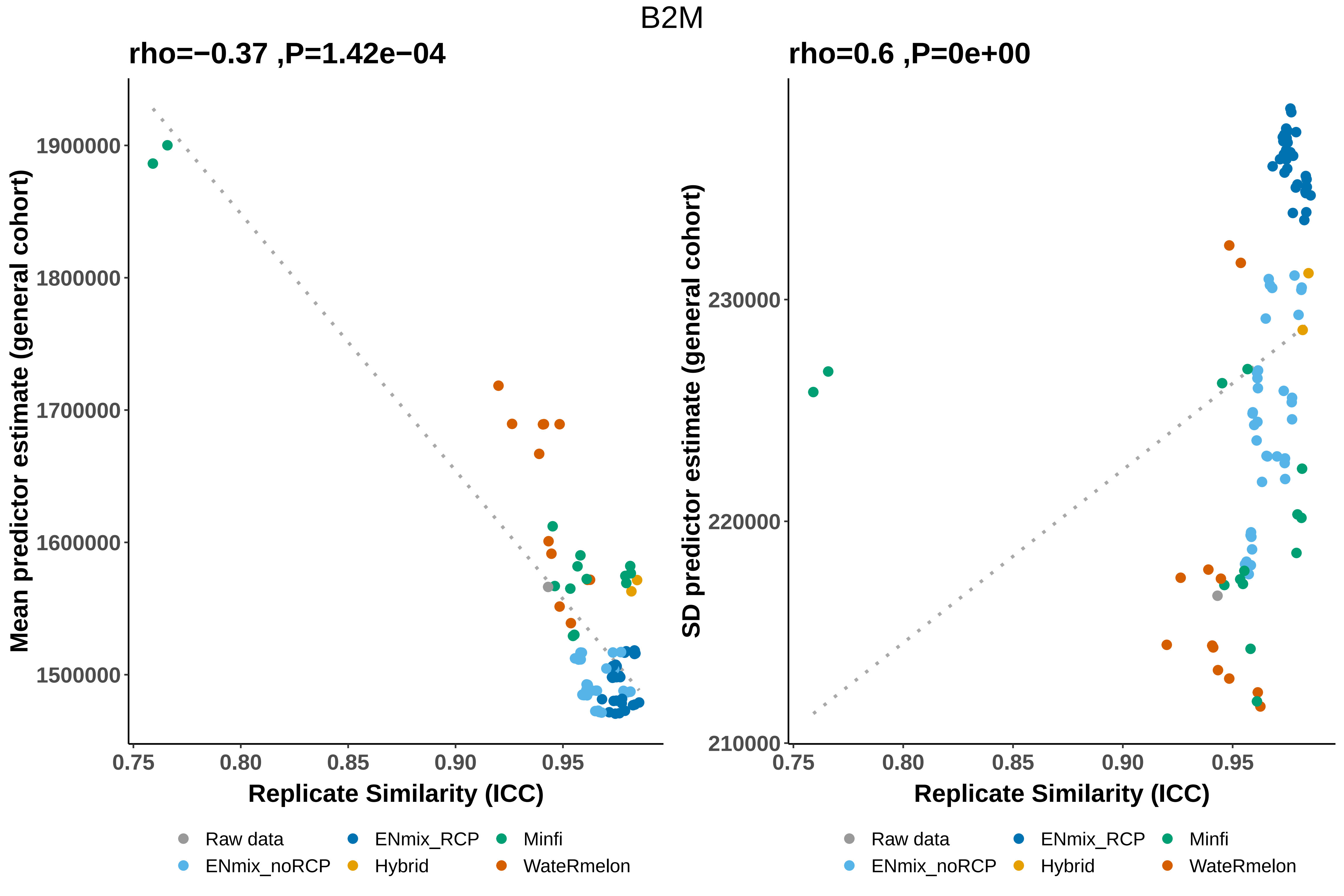
B2M
rho=−0.37 ,P=1.42e−04
rho=0.6 ,P=0e+00
Mean predictor estimate (general cohort)
SD predictor estimate (general cohort)
1900000
1800000
1700000
1600000
1500000
230000
220000
210000
0.75
0.80
0.85
0.90
0.95
Replicate Similarity (ICC)
Replicate Similarity (ICC)
Raw data
ENmix_noRCP
ENmix_RCP
Hybrid
Minfi
WateRmelon

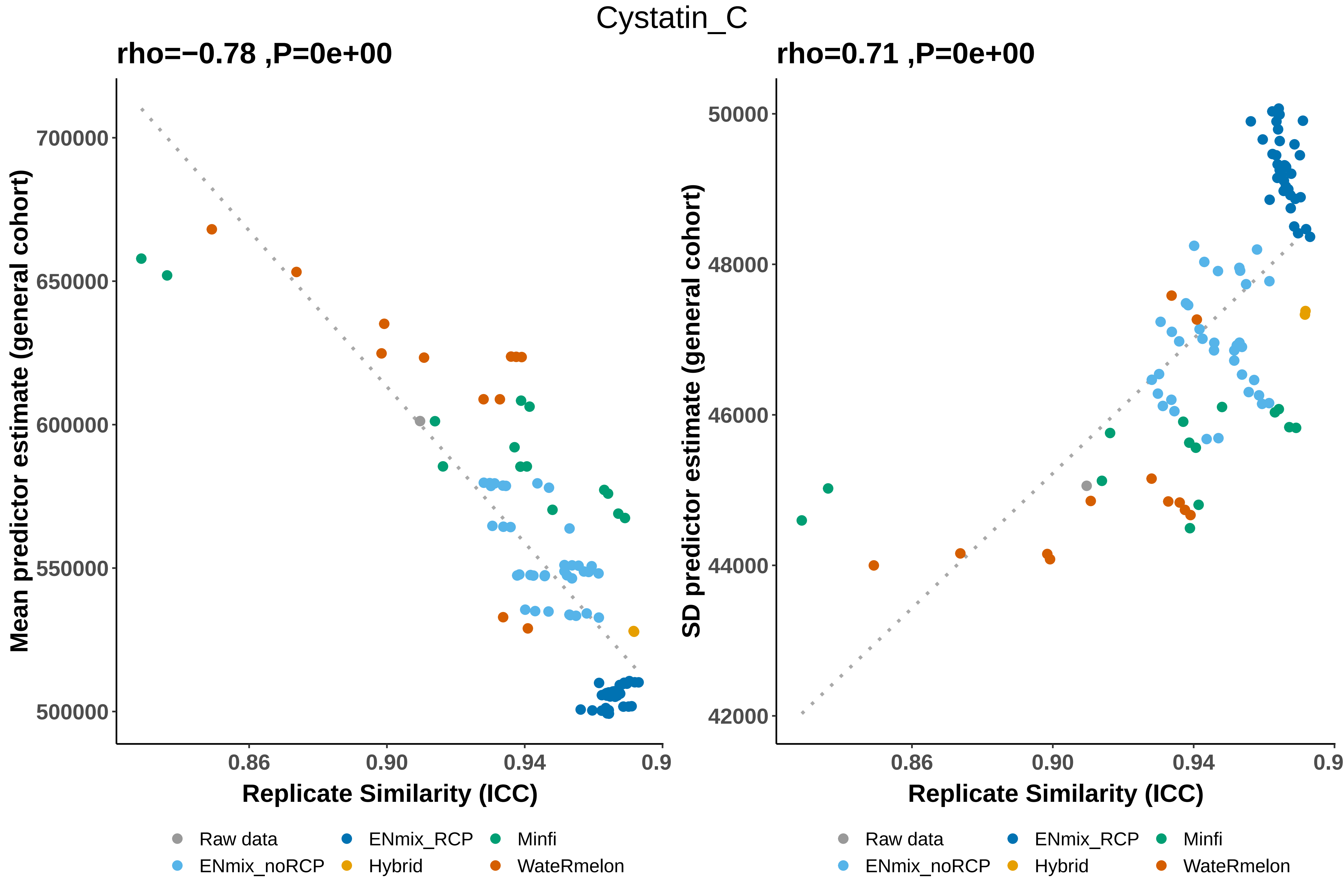
Cystatin_C
rho=−0.78 ,P=0e+00
rho=0.71 ,P=0e+00
Mean predictor estimate (general cohort)
SD predictor estimate (general cohort)
700000
650000
600000
550000
500000
50000
48000
46000
44000
42000
0.86
0.90
0.94
0.9
Replicate Similarity (ICC)
Raw data
ENmix_RCP
Minfi
ENmix_noRCP
Hybrid
WateRmelon

TIMP_1

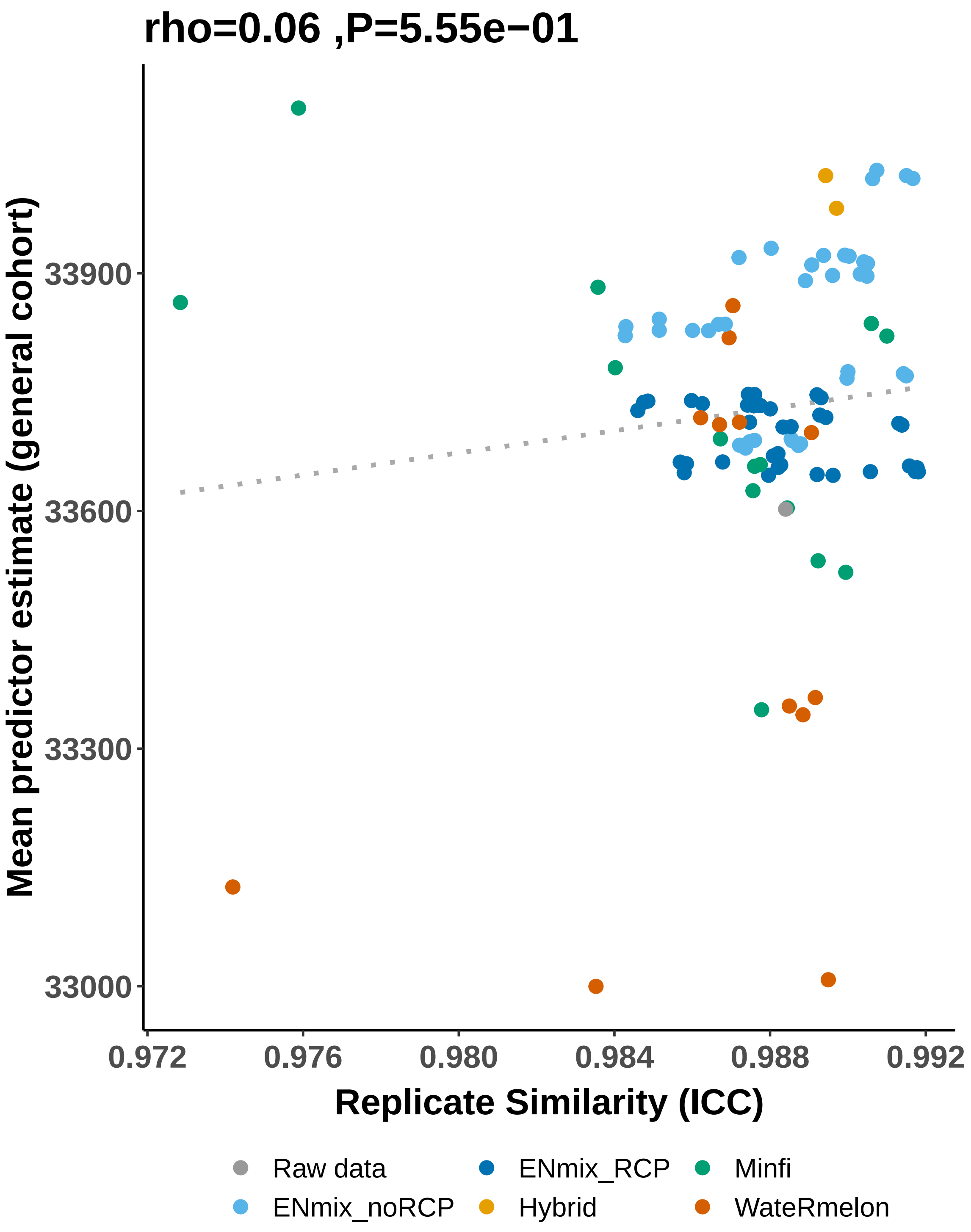

rho=0.06 ,P=5.55e−01
Mean predictor estimate (general cohort)
33000
33300
33600
33900
0.972
0.976
0.980
0.984
0.988
0.992
Replicate Similarity (ICC)
Raw data
ENmix_noRCP
ENmix_RCP
Hybrid
Minfi
WateRmelon

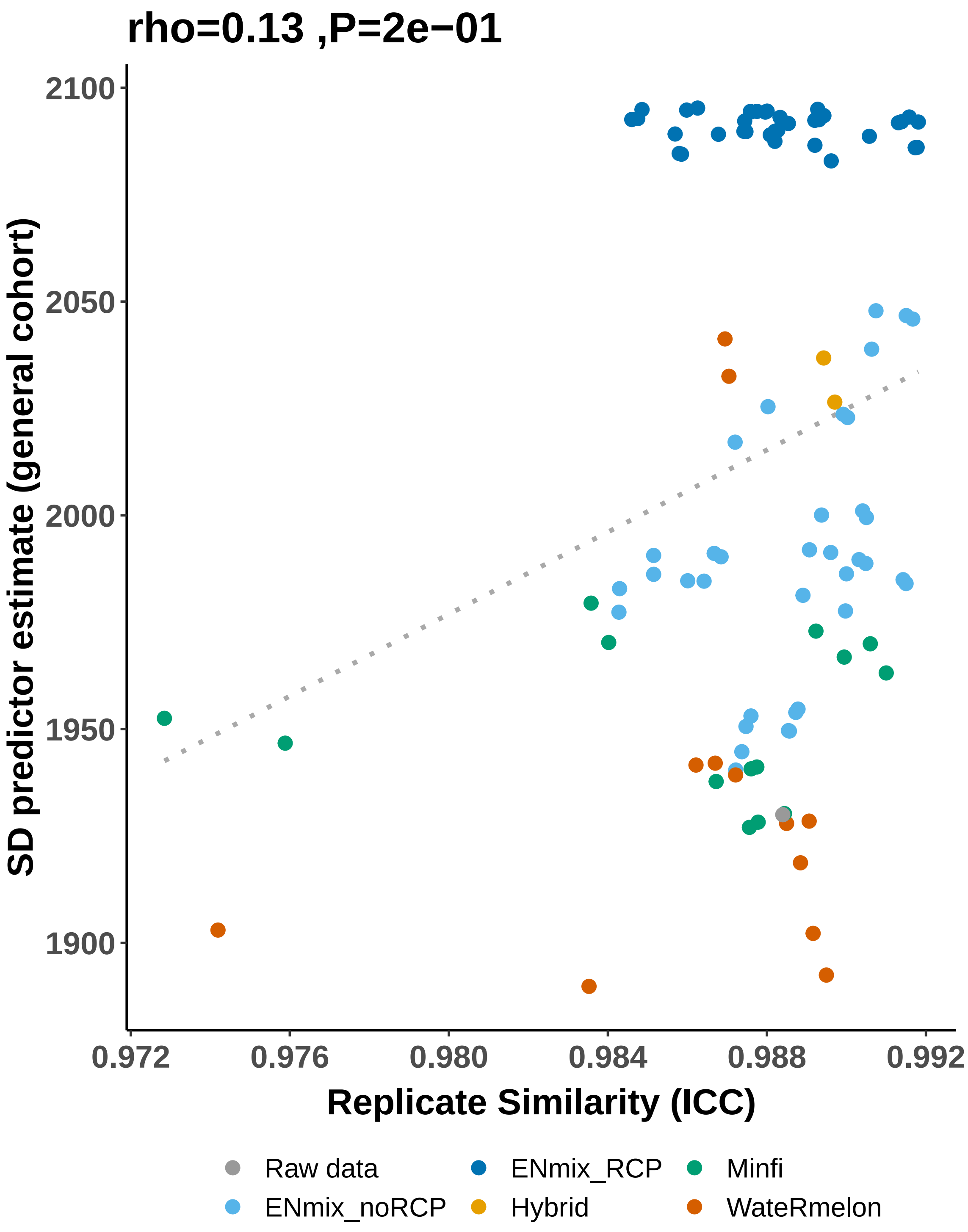

rho=0.13 ,P=2e−01
SD predictor estimate (general cohort)
1900
1950
2000
2050
2100
0.972
0.976
0.980
0.984
0.988
0.992
Replicate Similarity (ICC)
Raw data
ENmix_noRCP
ENmix_RCP
Hybrid
Minfi
WateRmelon

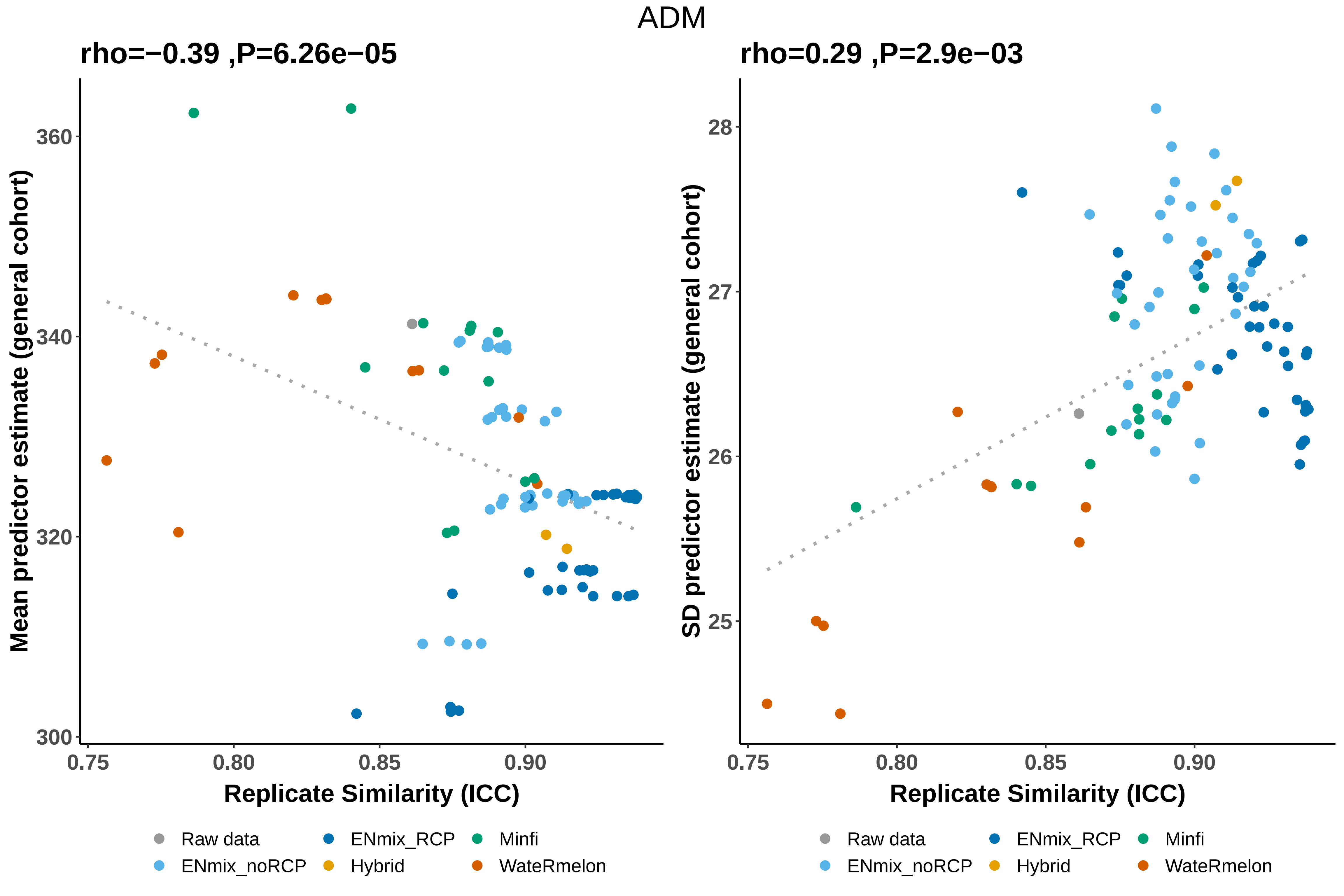

ADM
rho=−0.39 ,P=6.26e−05
rho=0.29 ,P=2.9e−03
Mean predictor estimate (general cohort)
SD predictor estimate (general cohort)
Replicate Similarity (ICC)
Replicate Similarity (ICC)
360
340
320
300
28
27
26
25
0.75
0.80
0.85
0.90
Raw data
ENmix_RCP
Minfi
ENmix_noRCP
Hybrid
WateRmelon

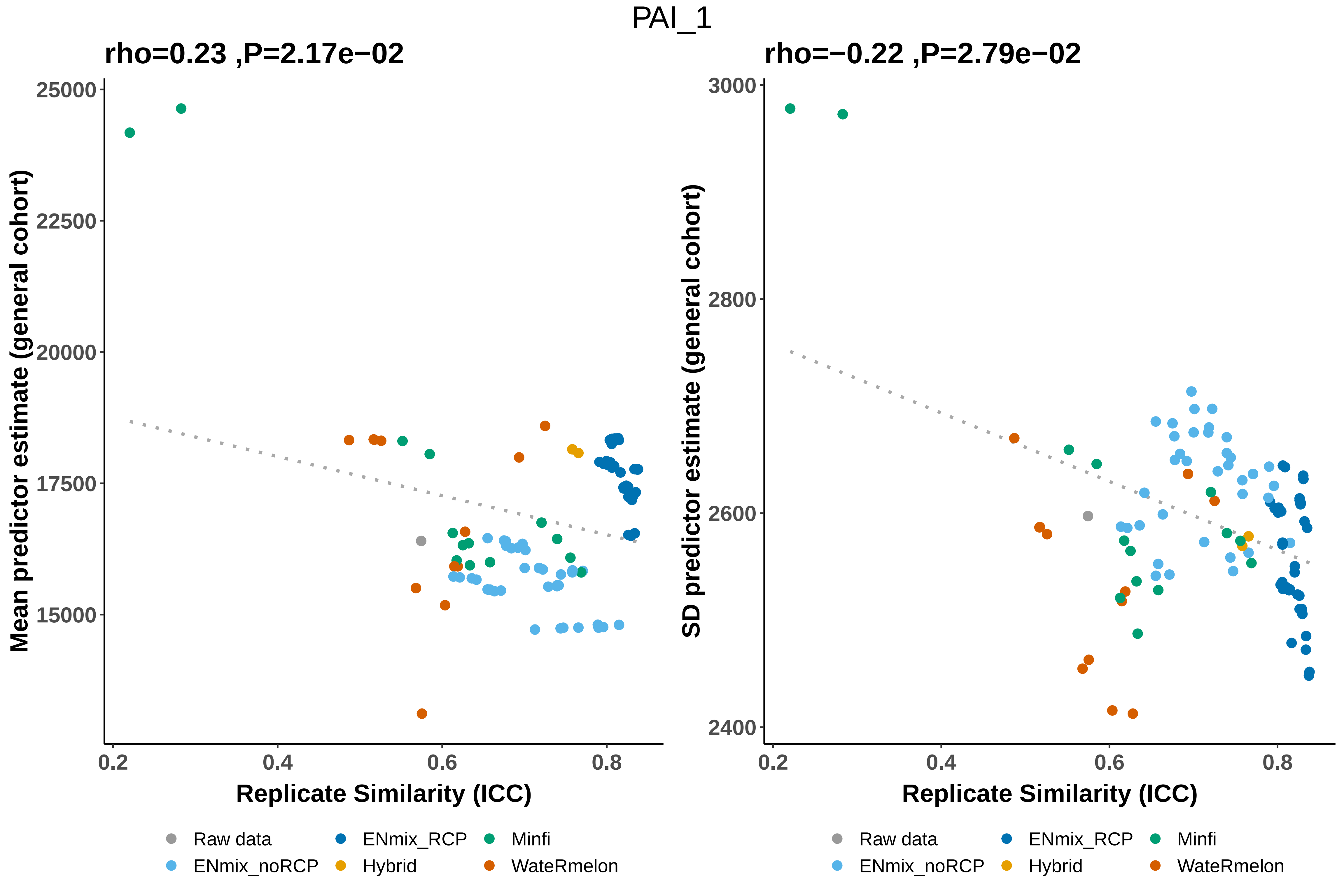

PAI_1
rho=0.23 ,P=2.17e−02
rho=−0.22 ,P=2.79e−02
Mean predictor estimate (general cohort)
SD predictor estimate (general cohort)
25000
22500
20000
17500
15000
3000
2800
2600
2400
0.2
0.4
0.6
0.8
Replicate Similarity (ICC)
Raw data
ENmix_noRCP
ENmix_RCP
Hybrid
Minfi
WateRmelon

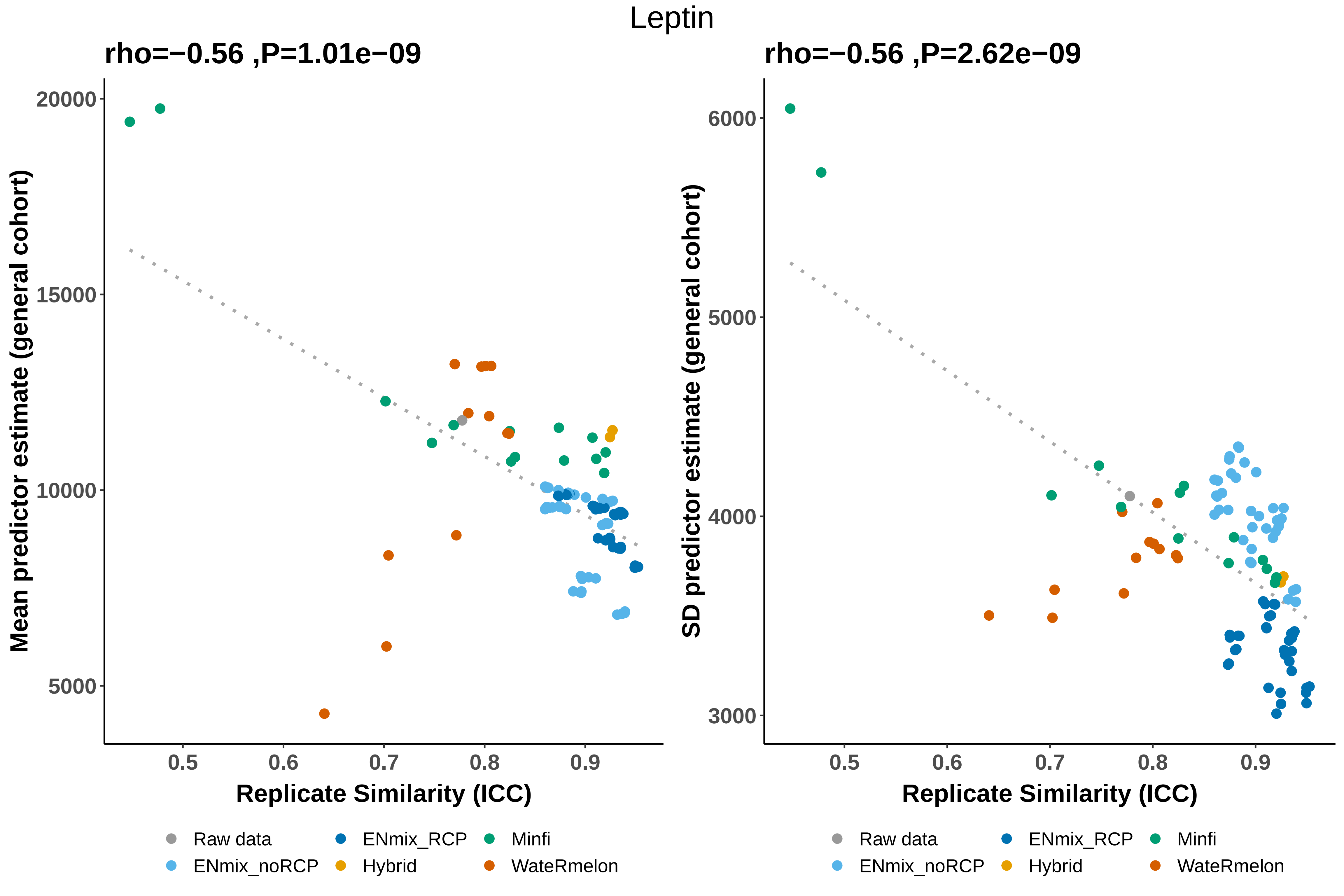

Leptin
rho=−0.56 ,P=1.01e−09
rho=−0.56 ,P=2.62e−09
Mean predictor estimate (general cohort)
SD predictor estimate (general cohort)
20000
15000
10000
5000
6000
5000
4000
3000
0.5
0.6
0.7
0.8
0.9
Replicate Similarity (ICC)
Raw data
ENmix_RCP
Minfi
ENmix_noRCP
Hybrid
WateRmelon

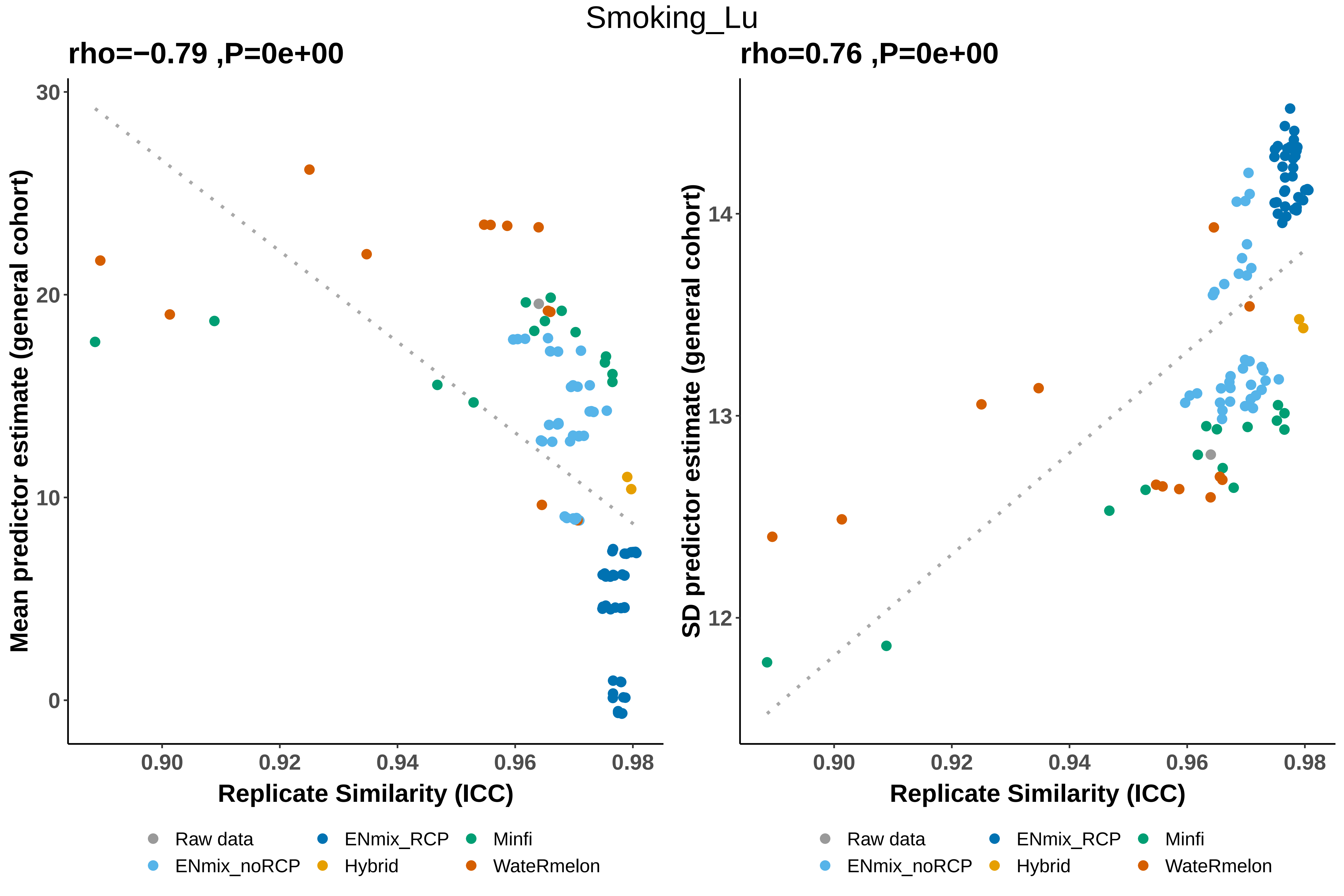

Smoking_Lu
rho=−0.79 ,P=0e+00
rho=0.76 ,P=0e+00
Mean predictor estimate (general cohort)
SD predictor estimate (general cohort)
30
20
10
0
14
13
12
0.90
0.92
0.94
0.96
0.98
Replicate Similarity (ICC)
Raw data
ENmix_RCP
Minfi
ENmix_noRCP
Hybrid
WateRmelon

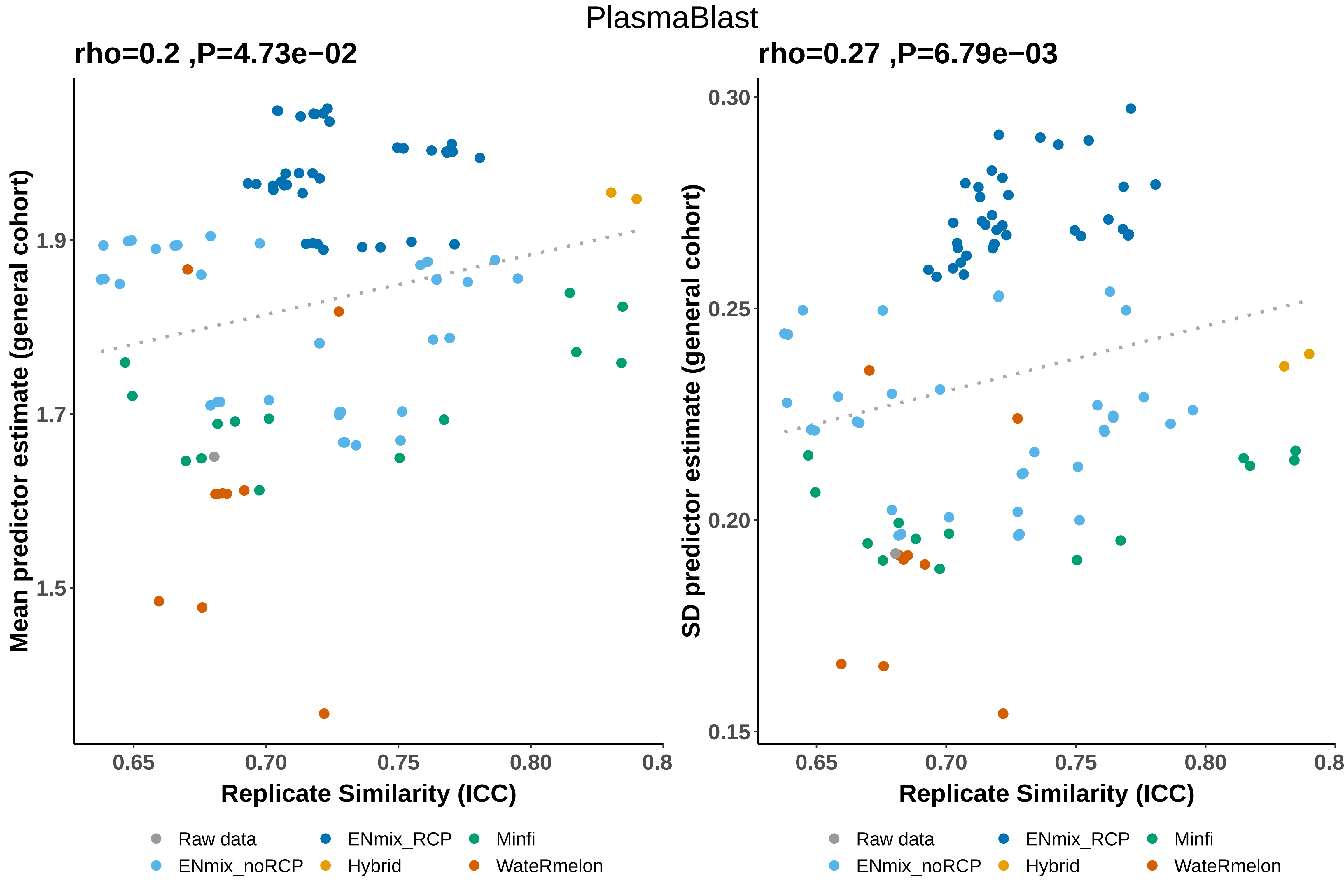
PlasmaBlast
rho=0.2 ,P=4.73e−02
rho=0.27 ,P=6.79e−03
Mean predictor estimate (general cohort)
SD predictor estimate (general cohort)
1.9
1.7
1.5
0.30
0.25
0.20
0.15
0.65
0.70
0.75
0.80
0.8
Replicate Similarity (ICC)
Raw data
ENmix_RCP
Minfi
ENmix_noRCP
Hybrid
WateRmelon

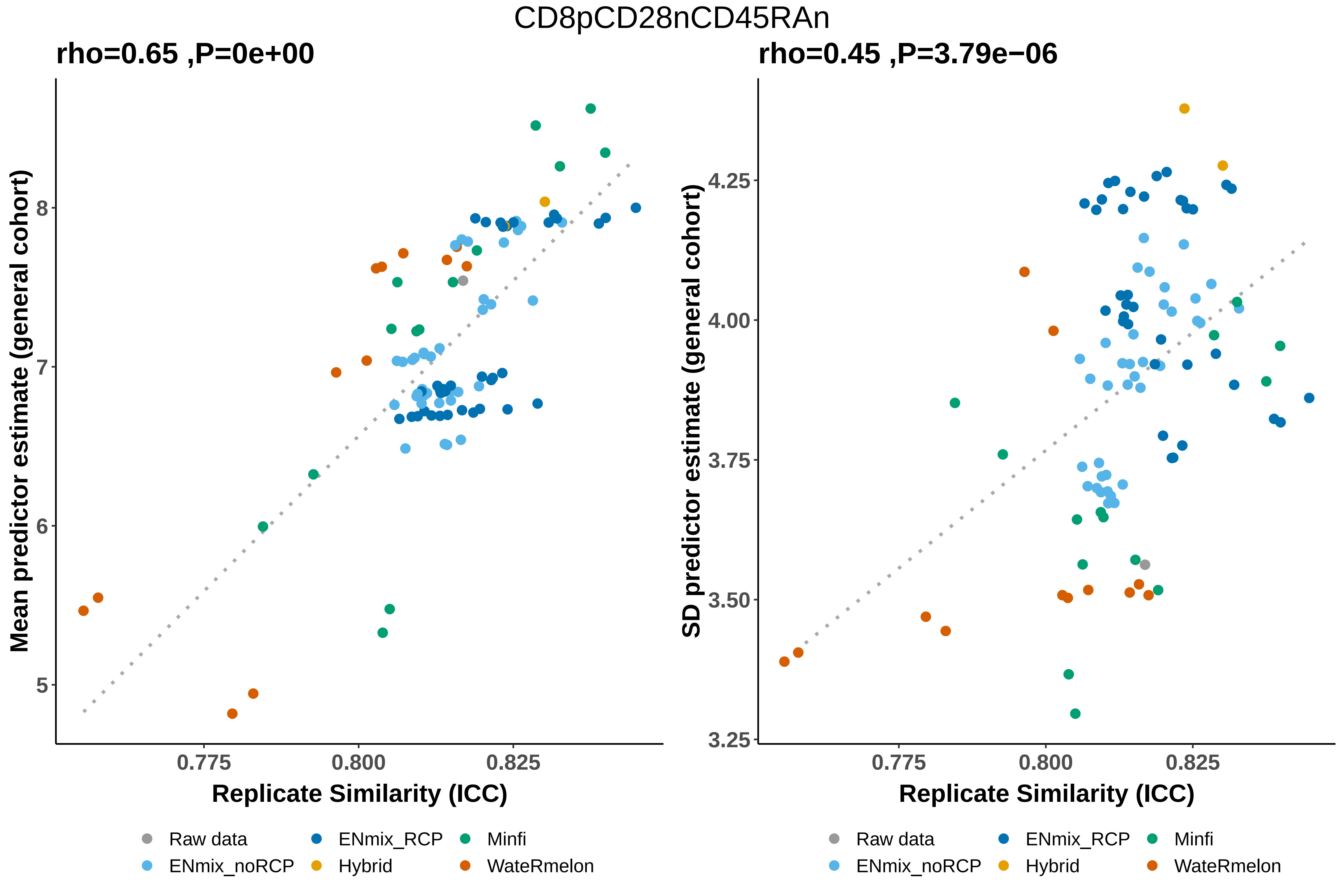
CD8pCD28nCD45RAn
rho=0.65 ,P=0e+00
rho=0.45 ,P=3.79e−06
Mean predictor estimate (general cohort)
SD predictor estimate (general cohort)
Replicate Similarity (ICC)
0.775
0.800
0.825
5
6
7
8
3.25
3.50
3.75
4.00
4.25
Raw data
ENmix_noRCP
ENmix_RCP
Hybrid
Minfi
WateRmelon

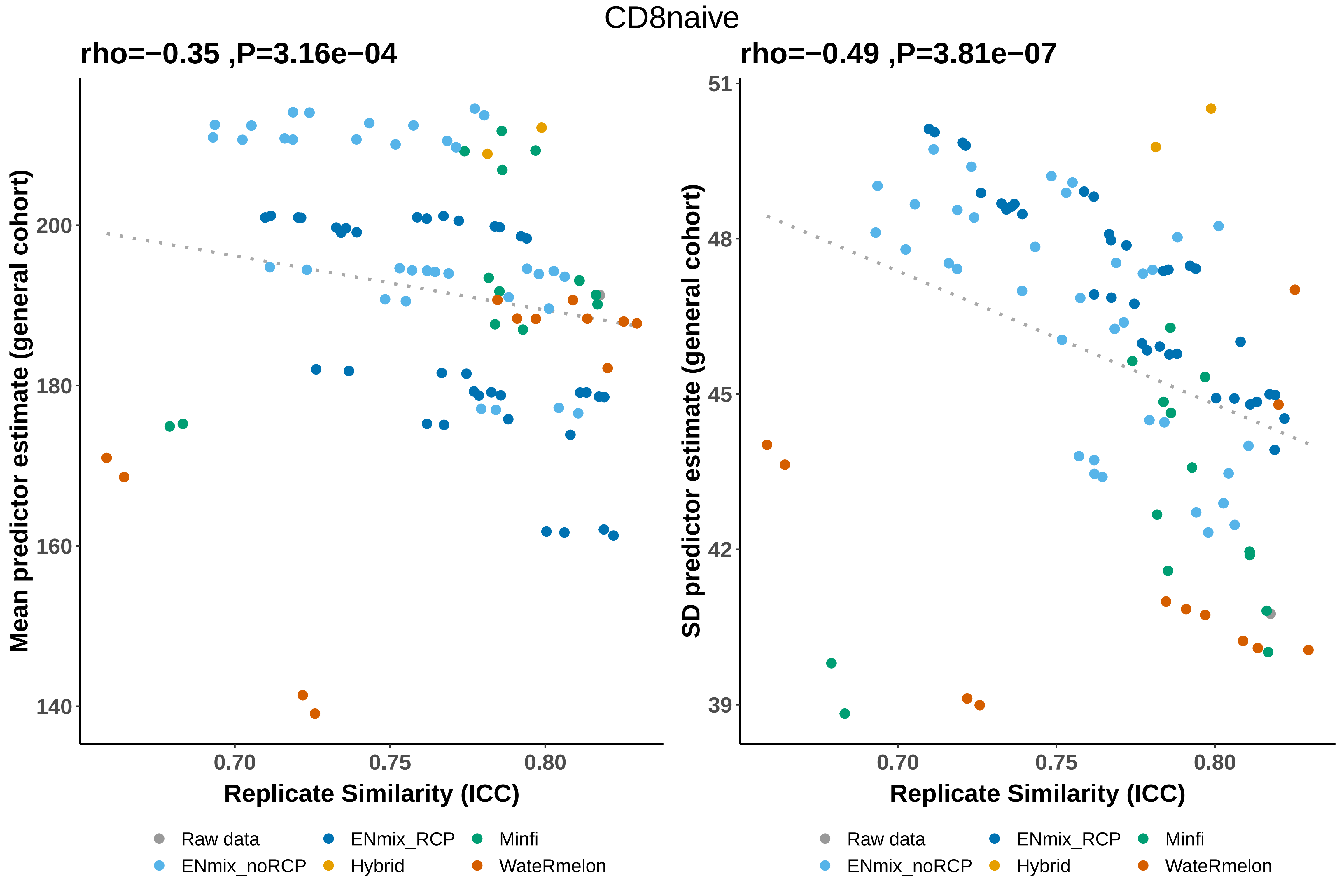

CD8naive
rho=−0.35 ,P=3.16e−04
rho=−0.49 ,P=3.81e−07
Mean predictor estimate (general cohort)
SD predictor estimate (general cohort)
200
180
160
140
51
48
45
42
39
0.70
0.75
0.80
Replicate Similarity (ICC)
Raw data
ENmix_noRCP
ENmix_RCP
Hybrid
Minfi
WateRmelon

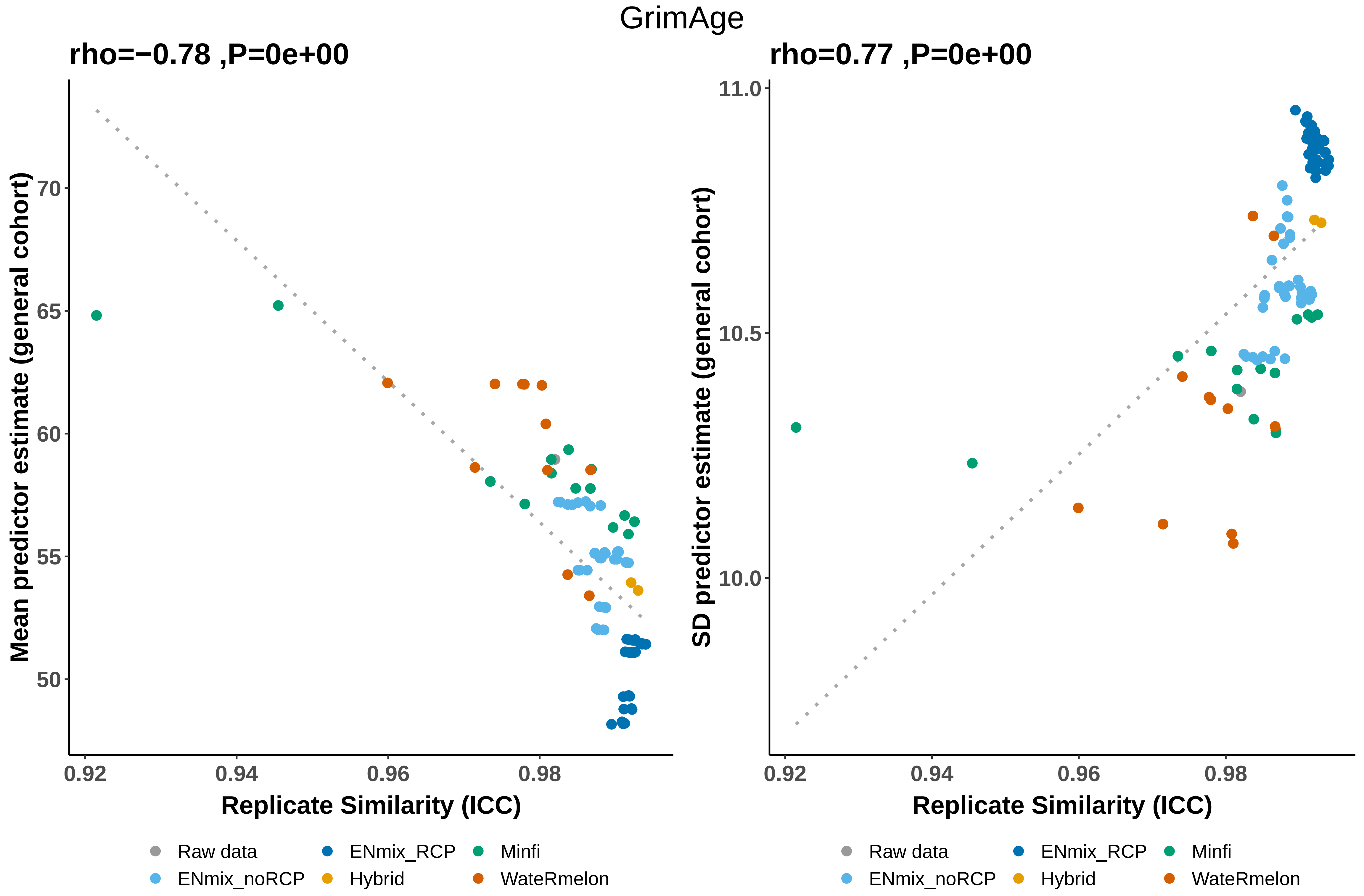

GrimAge
rho=−0.78 ,P=0e+00
rho=0.77 ,P=0e+00
Mean predictor estimate (general cohort)
SD predictor estimate (general cohort)
Replicate Similarity (ICC)
Replicate Similarity (ICC)
70
65
60
55
50
11.0
10.5
10.0
0.92
0.94
0.96
0.98
Raw data
ENmix_RCP
Minfi
ENmix_noRCP
Hybrid
WateRmelon

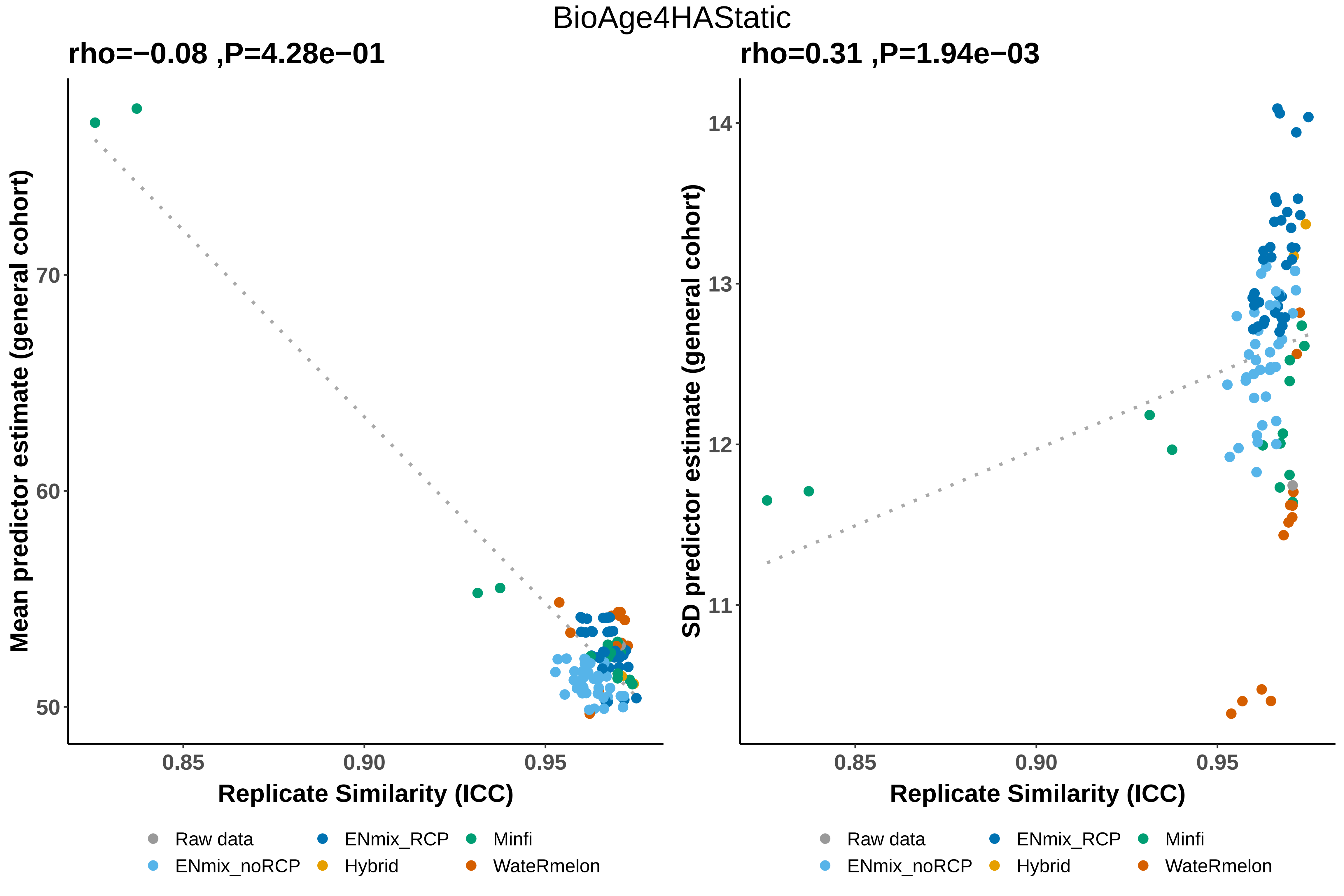

BioAge4HAStatic
rho=−0.08 ,P=4.28e−01
rho=0.31 ,P=1.94e−03
Mean predictor estimate (general cohort)
SD predictor estimate (general cohort)
70
60
50
14
13
12
11
0.85
0.90
0.95
Replicate Similarity (ICC)
Raw data
ENmix_RCP
Minfi
ENmix_noRCP
Hybrid
WateRmelon
