# Supplementary material for: Significant variation in the performance of DNA methylation predictors across data preprocessing and normalization strategies
Source: Genome Biol. 2022 Oct 24;23:225. doi: 10.1186/s13059-022-02793-w (PMC9590227; doi:10.1186/s13059-022-02793-w)
Supplement: Supplementary file 7 — Additional file 7. Figures that visualize the relationship between ICC and replicate sample size for each DNAm predictor. [file 13059_2022_2793_MOESM7_ESM.pdf]

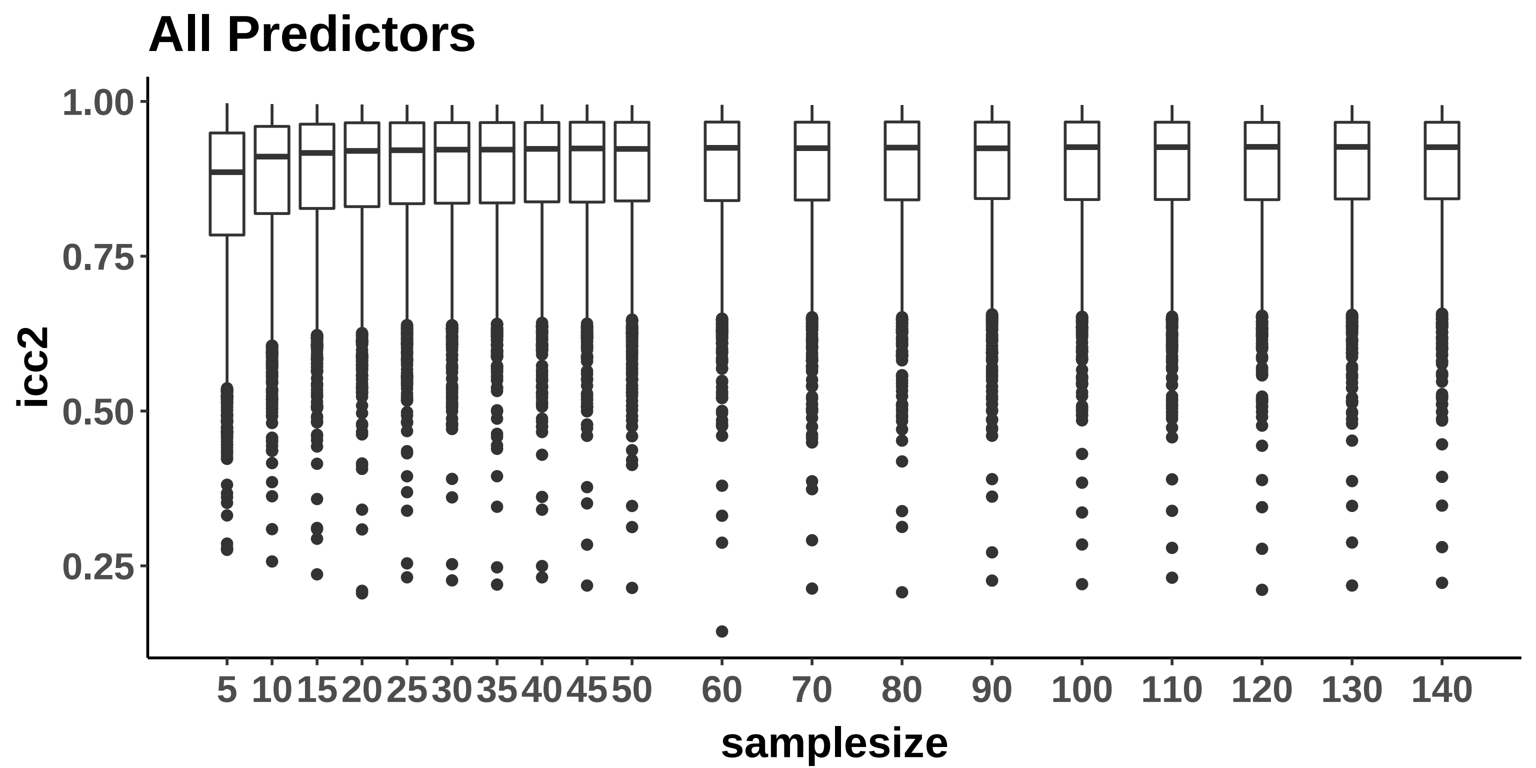
All Predictors
icc2
1.00
0.75
0.50
0.25
5
10
15
20
25
30
35
40
45
50
60
70
80
90
100
110
120
130
140
samplesize

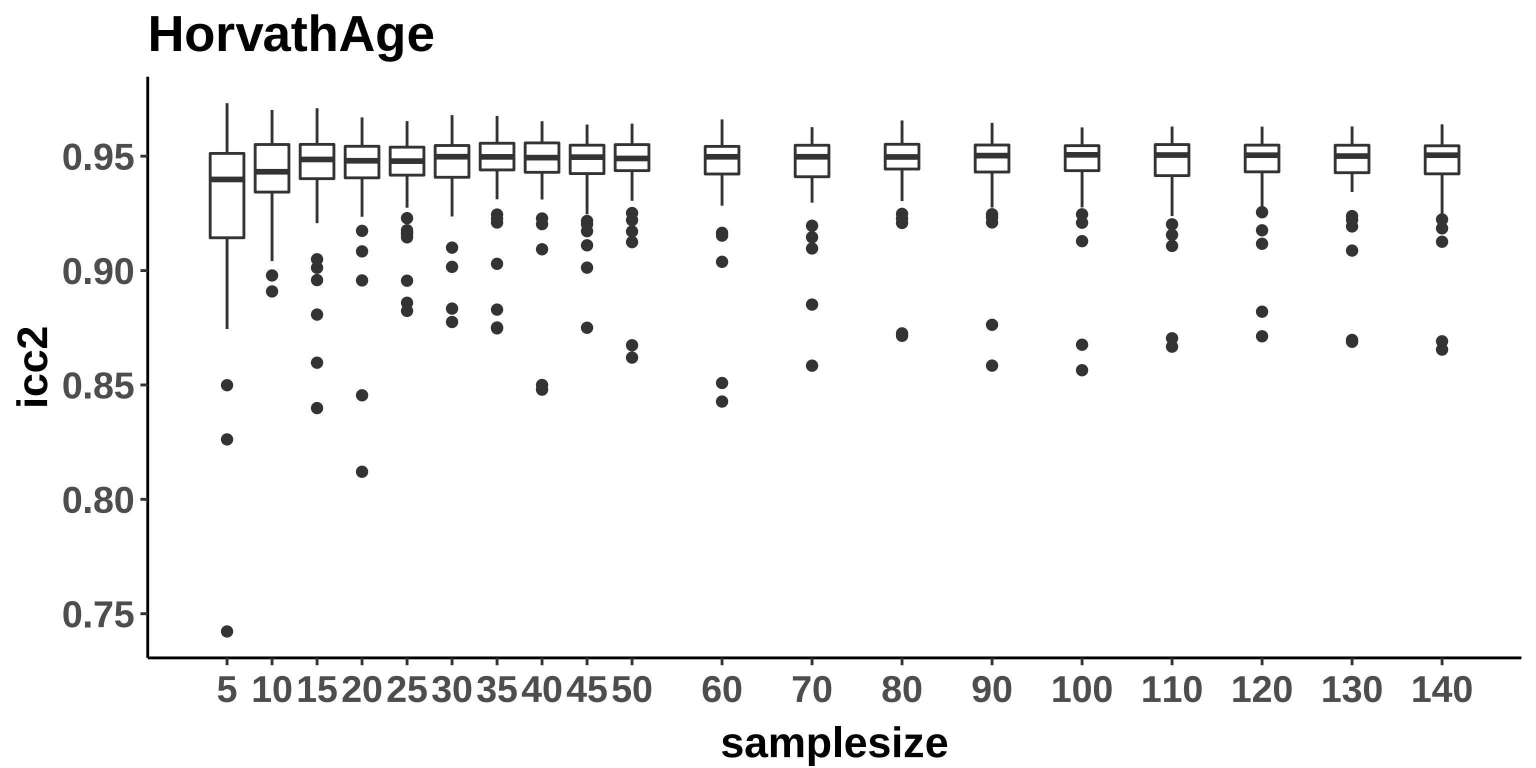
HorvathAge
icc2
0.95
0.90
0.85
0.80
0.75
5 10 15 20 25 30 35 40 45 50 60 70 80 90 100 110 120 130 140
samplesize

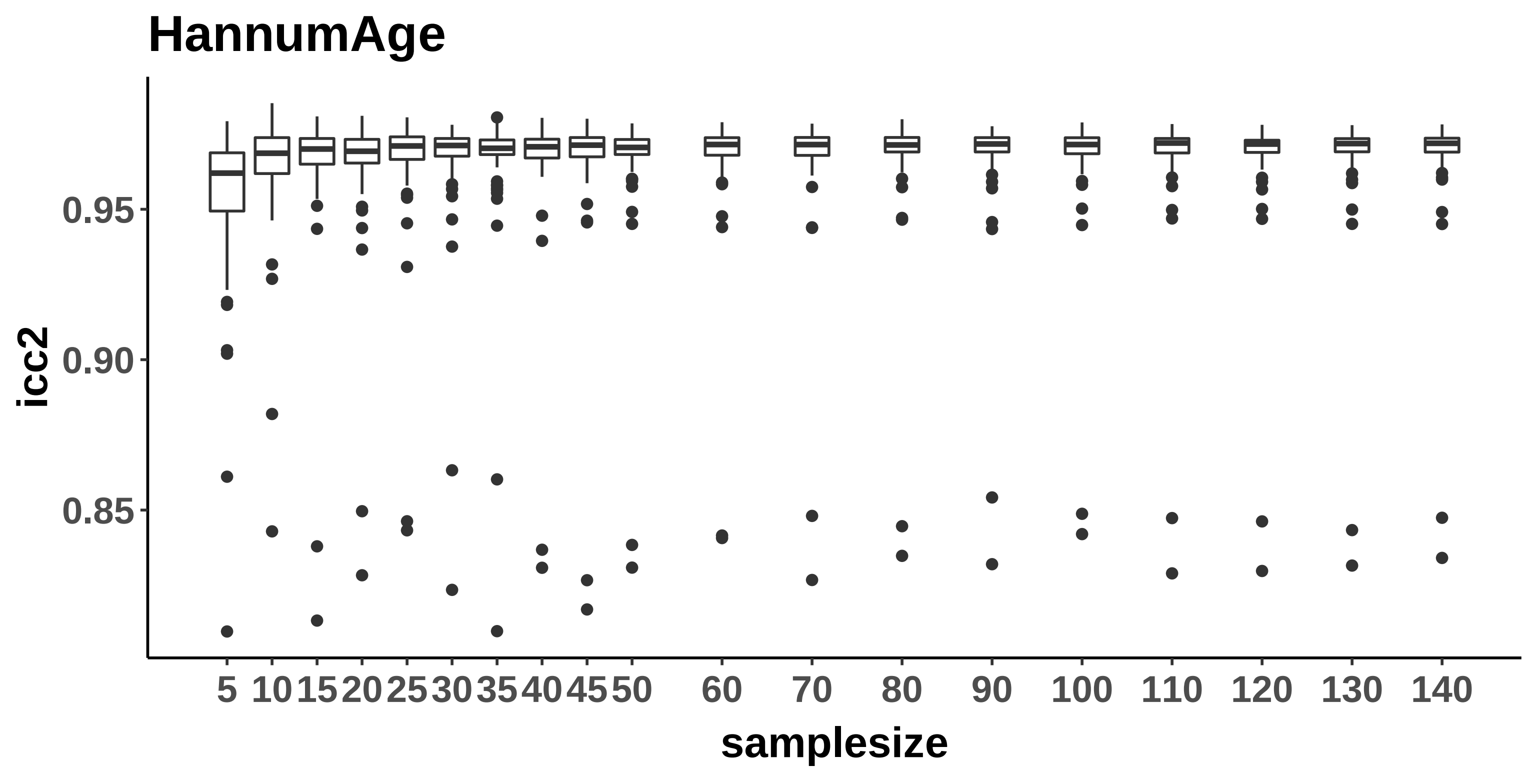

HannumAge
icc2
0.95
0.90
0.85
samplesize
5
10
15
20
25
30
35
40
45
50
60
70
80
90
100
110
120
130
140

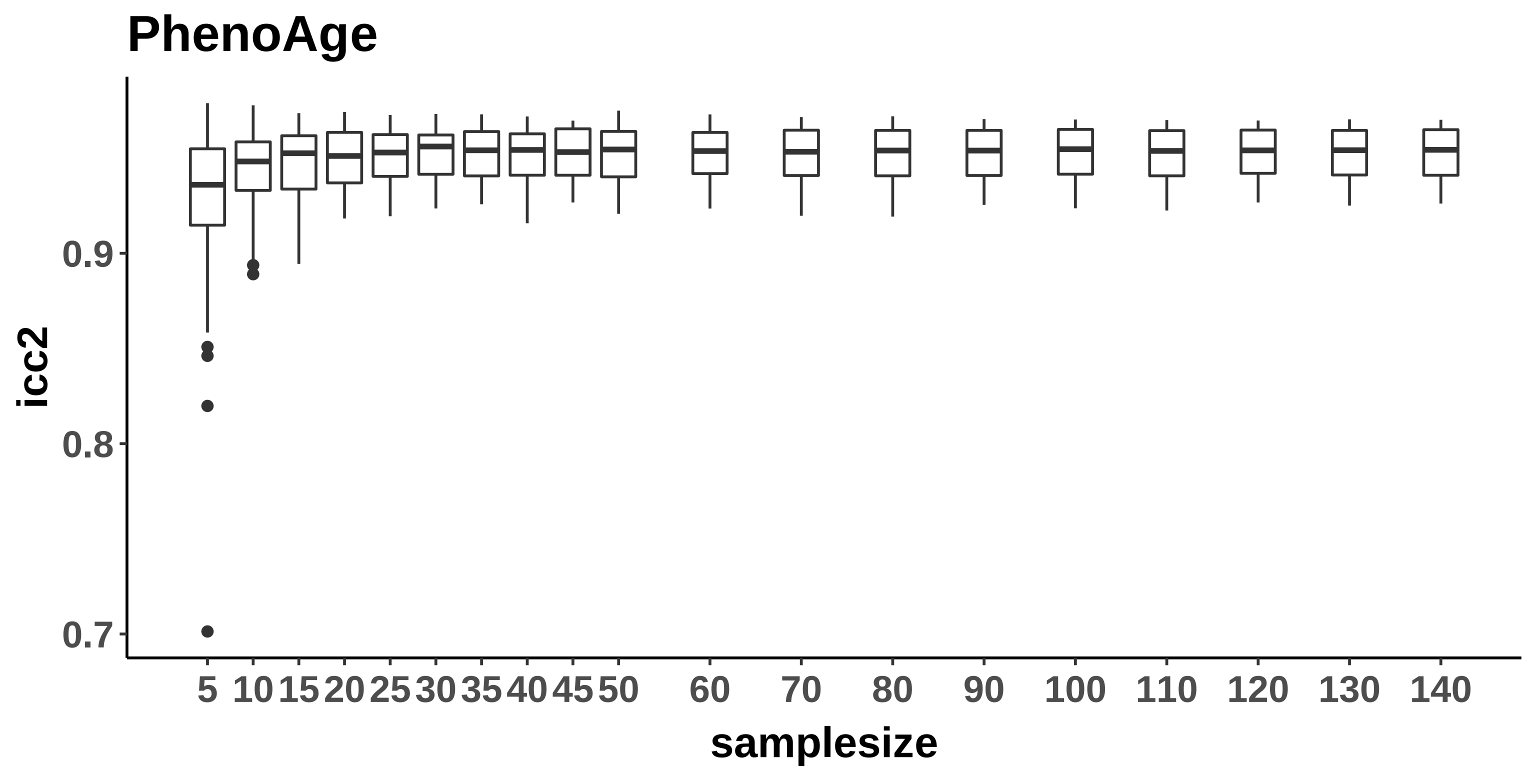
PhenoAge
icc2
0.9
0.8
0.7
5
10
15
20
25
30
35
40
45
50
60
70
80
90
100
110
120
130
140
samplesize

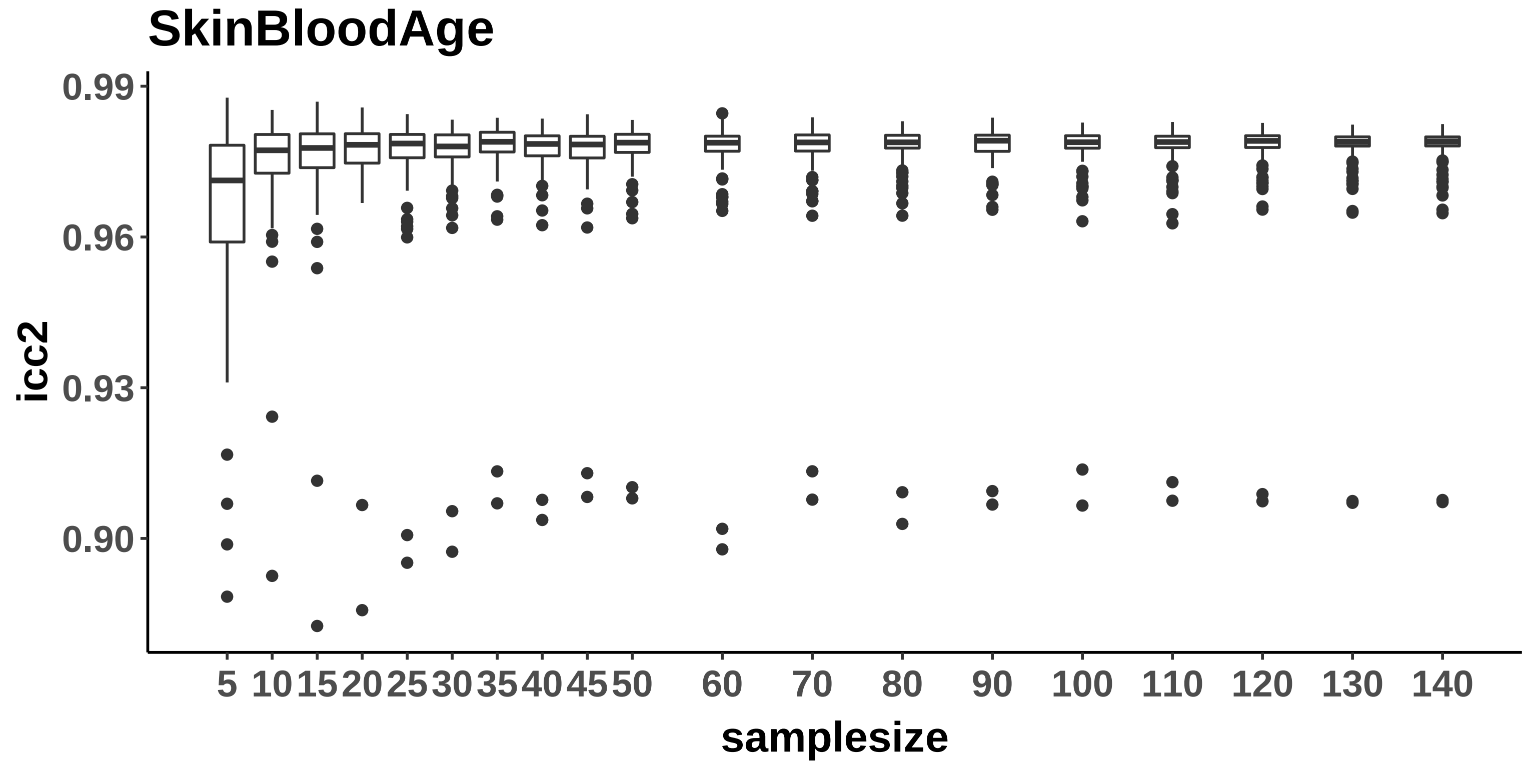
SkinBloodAge
0.99
0.96
0.93
0.90
icc2
5
10
15
20
25
30
35
40
45
50
60
70
80
90
100
110
120
130
140
samplesize

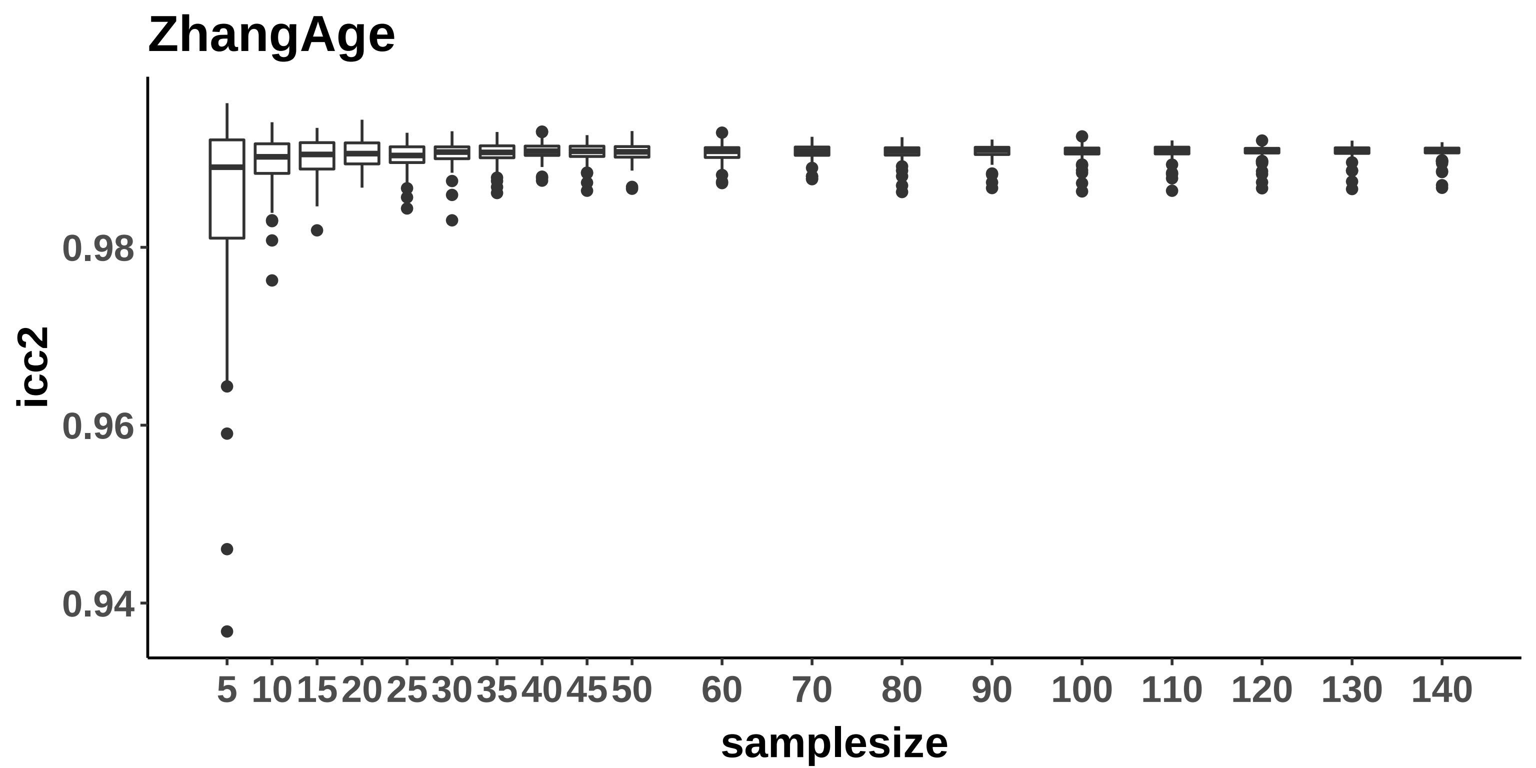

ZhangAge
icc2
0.98
0.96
0.94
5
10
15
20
25
30
35
40
45
50
60
70
80
90
100
110
120
130
140
samplesize

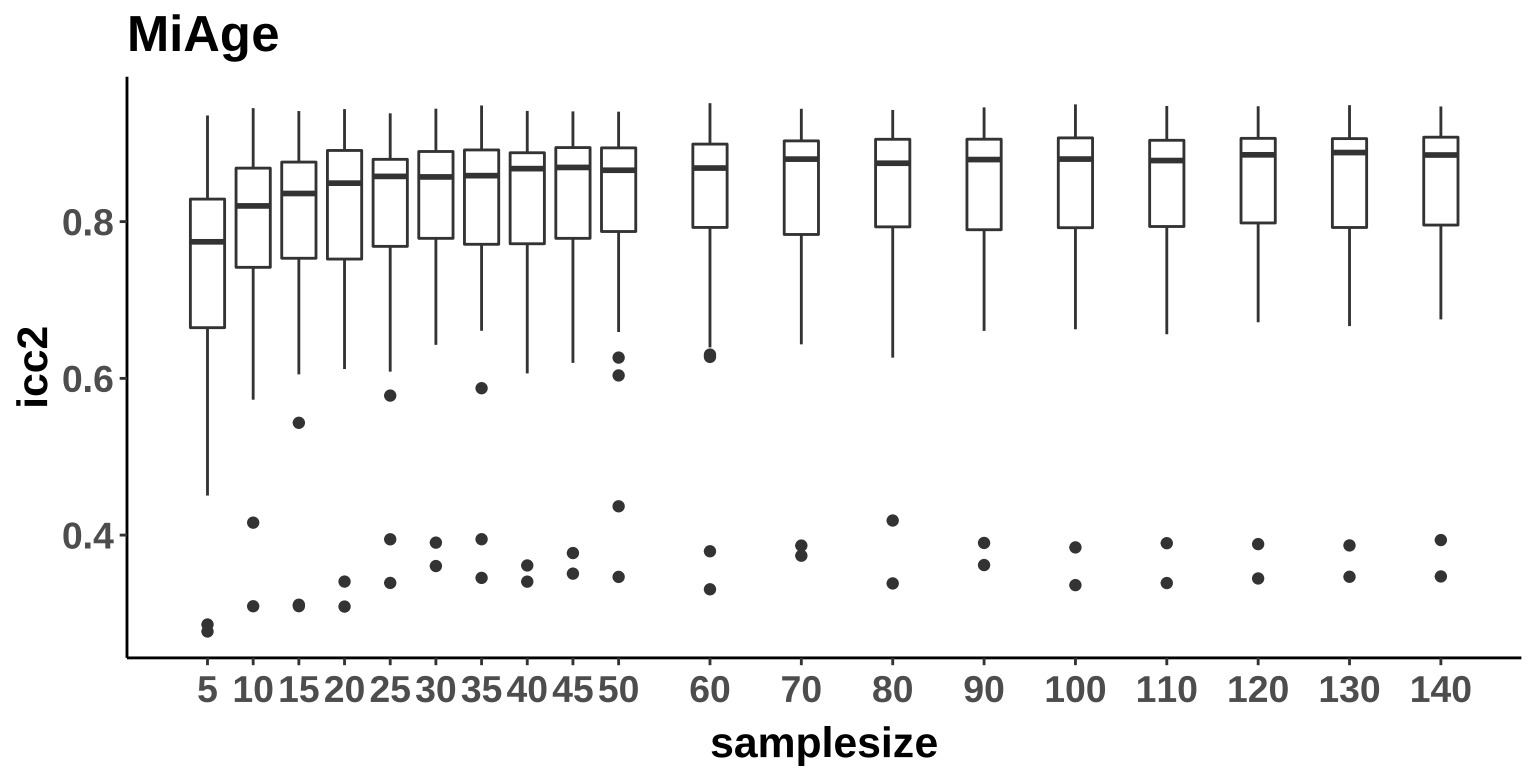
MiAge
icc2
0.8
0.6
0.4
5
10
15
20
25
30
35
40
45
50
60
70
80
90
100
110
120
130
140
samplesize

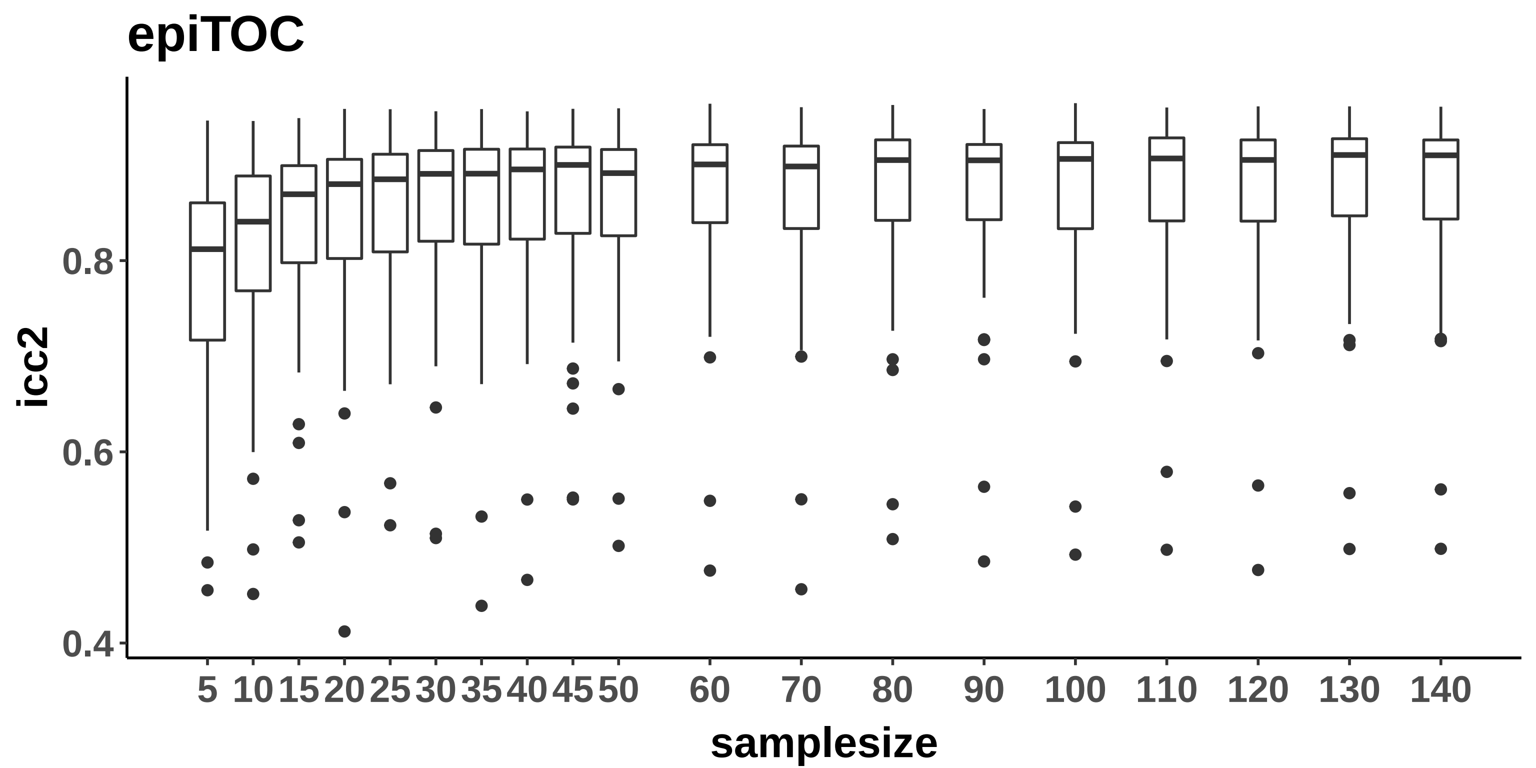
epiTOC
icc2
0.8
0.6
0.4
5
10
15
20
25
30
35
40
45
50
60
70
80
90
100
110
120
130
140
samplesize

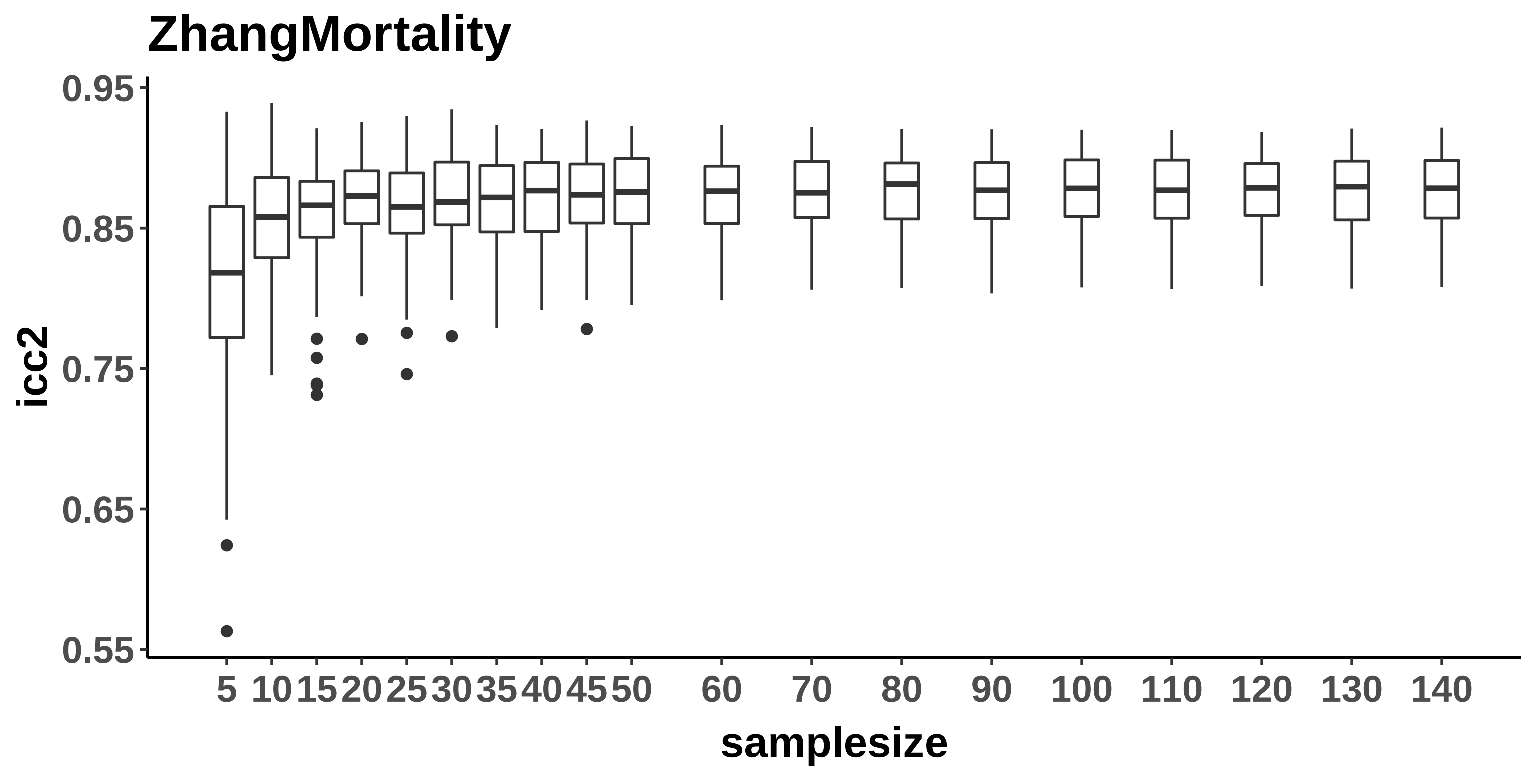

ZhangMortality
0.95
0.85
0.75
0.65
0.55
icc2
5
10
15
20
25
30
35
40
45
50
60
70
80
90
100
110
120
130
140
samplesize

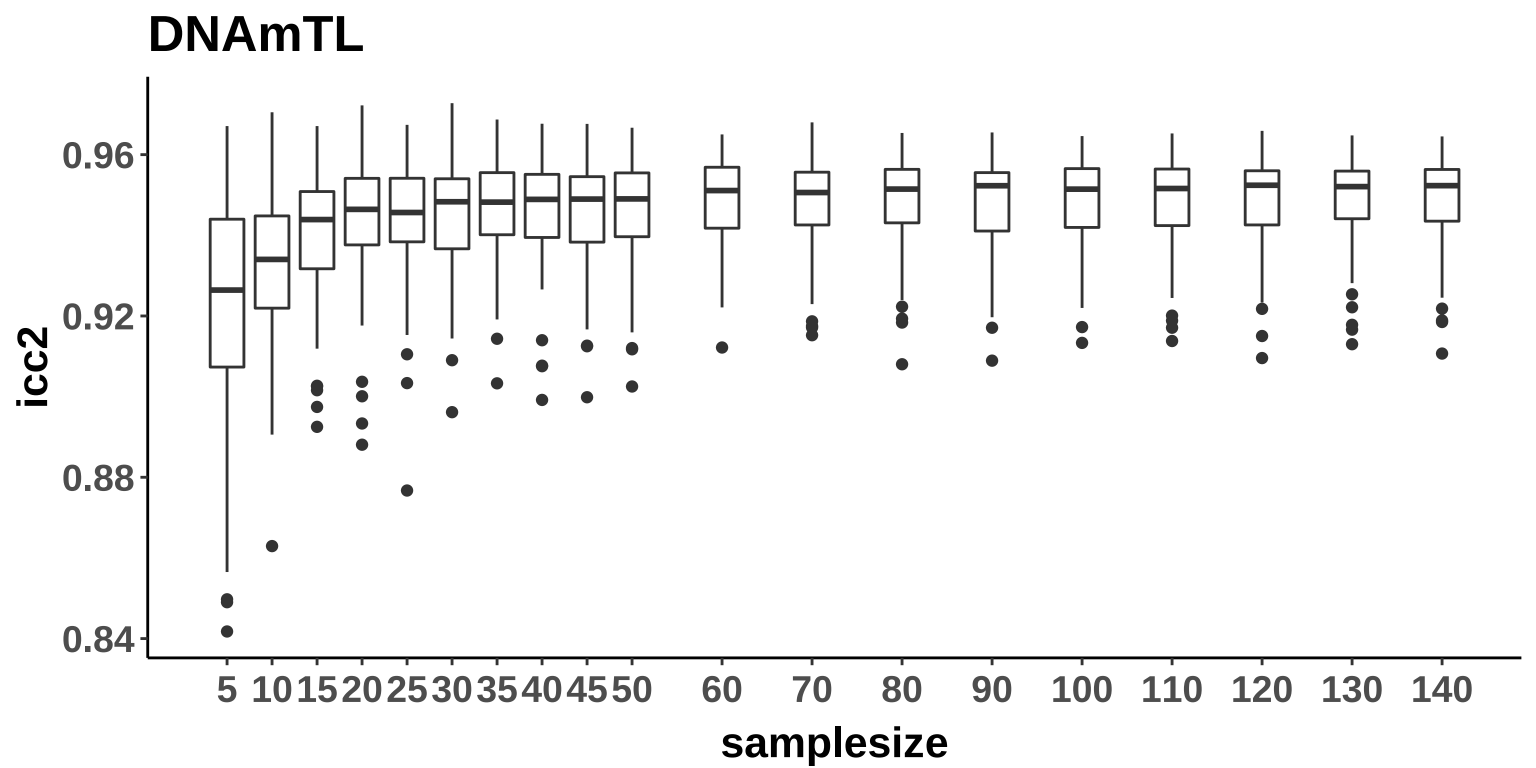
DNAmTL
icc2
0.96
0.92
0.88
0.84
5
10
15
20
25
30
35
40
45
50
60
70
80
90
100
110
120
130
140
samplesize

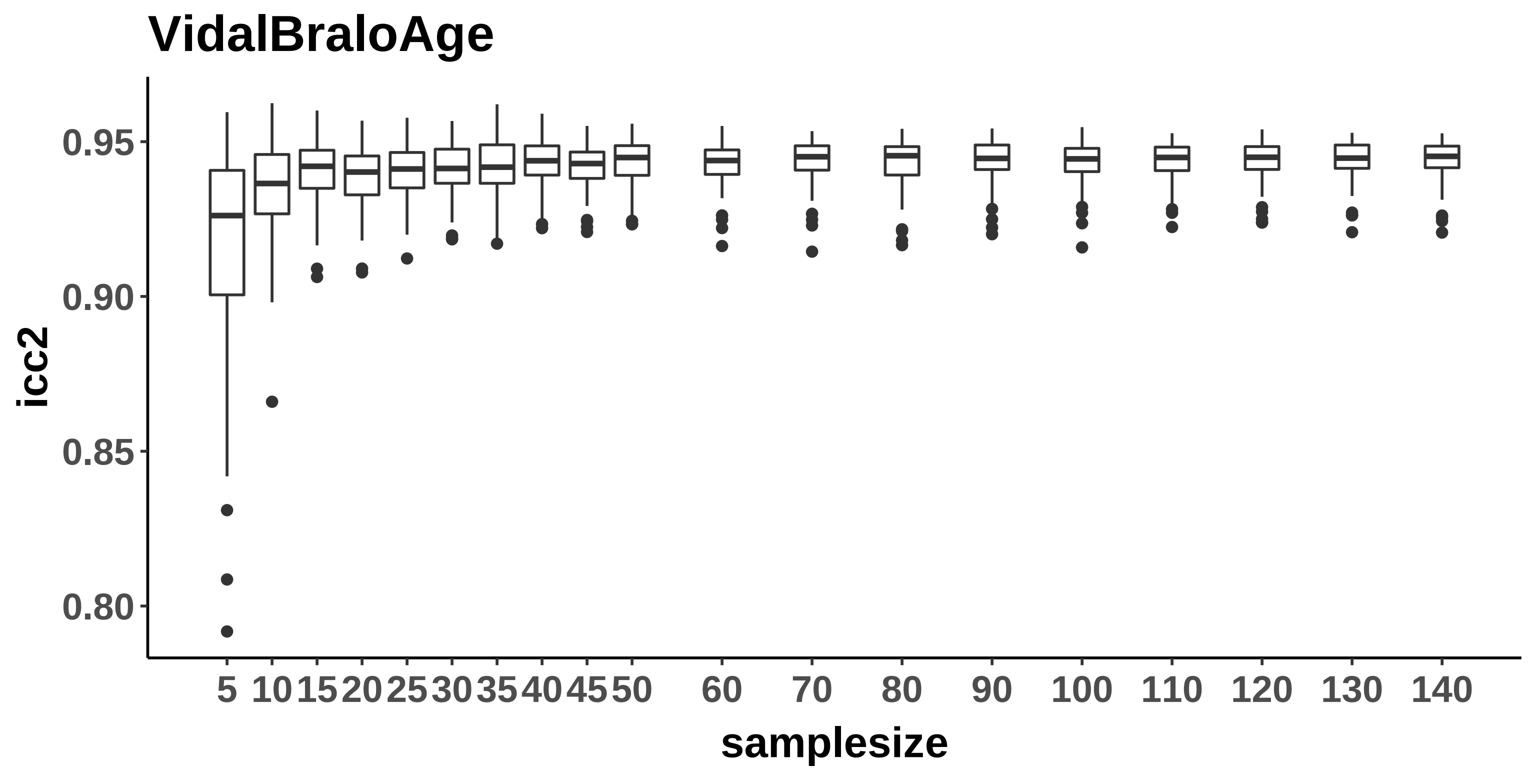
VidalBraloAge
icc2
0.95
0.90
0.85
0.80
samplesize
5
10
15
20
25
30
35
40
45
50
60
70
80
90
100
110
120
130
140

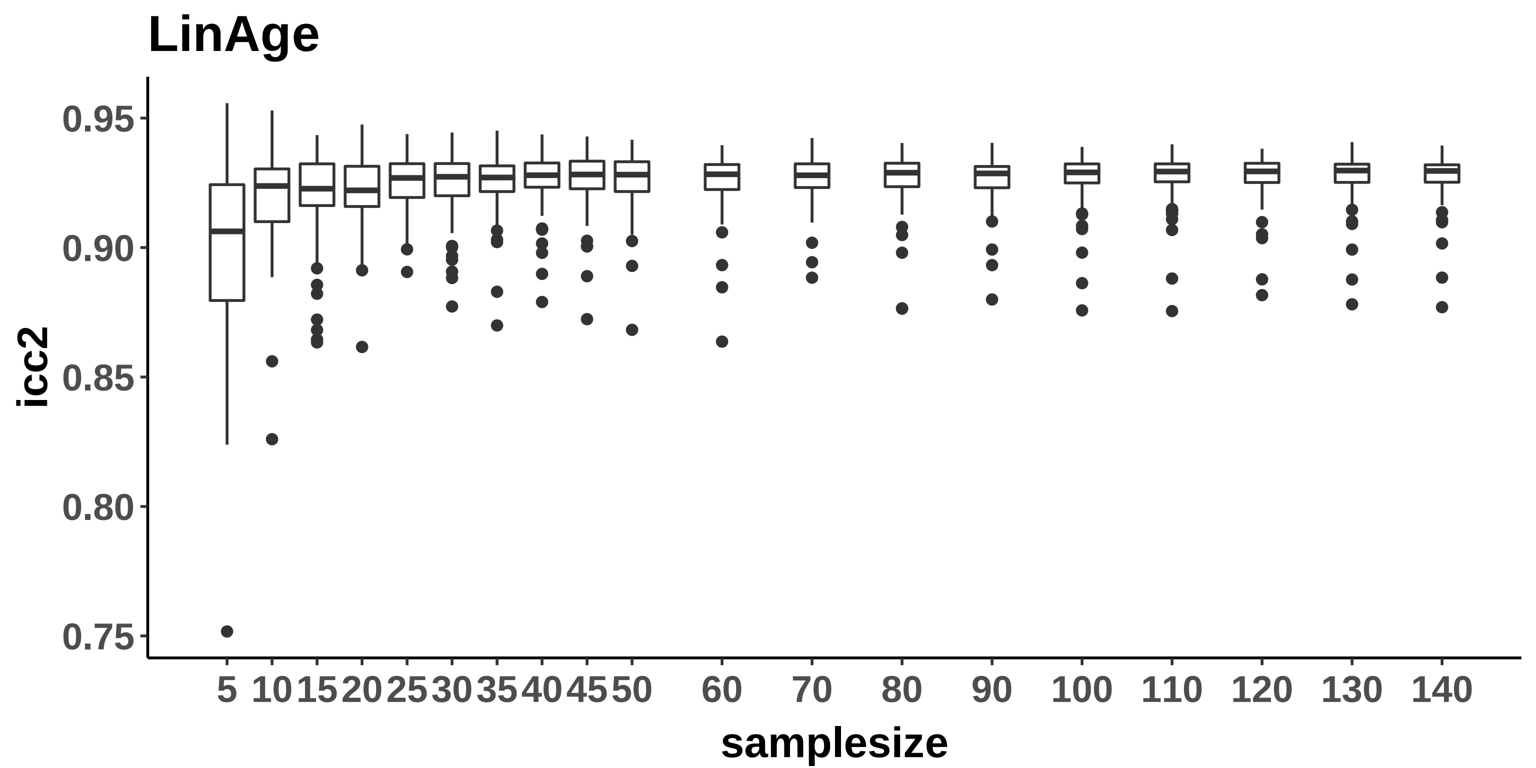

LinAge
icc2
0.95
0.90
0.85
0.80
0.75
5
10
15
20
25
30
35
40
45
50
60
70
80
90
100
110
120
130
140
samplesize

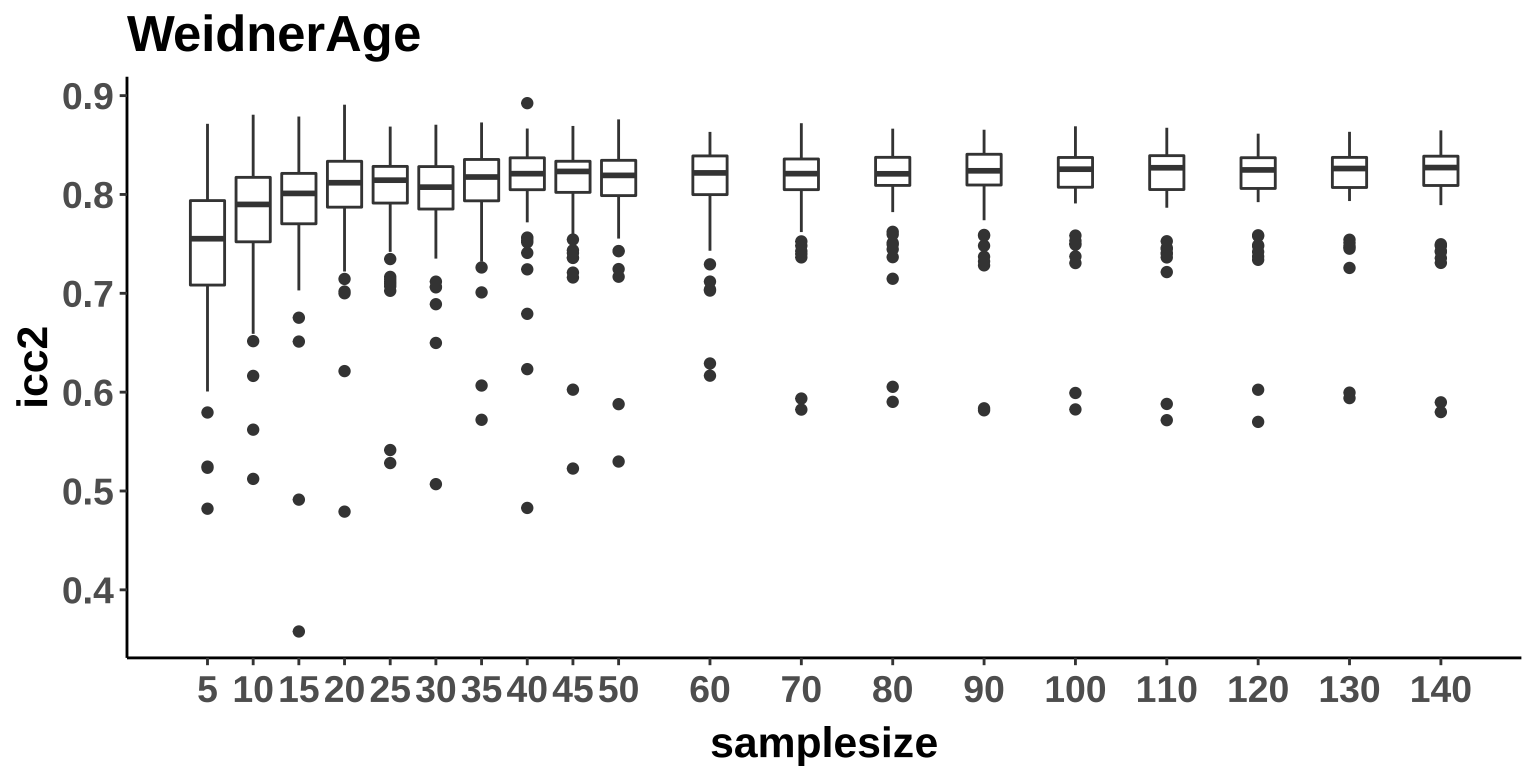

WeidnerAge
icc2
0.9
0.8
0.7
0.6
0.5
0.4
5
10
15
20
25
30
35
40
45
50
60
70
80
90
100
110
120
130
140
samplesize

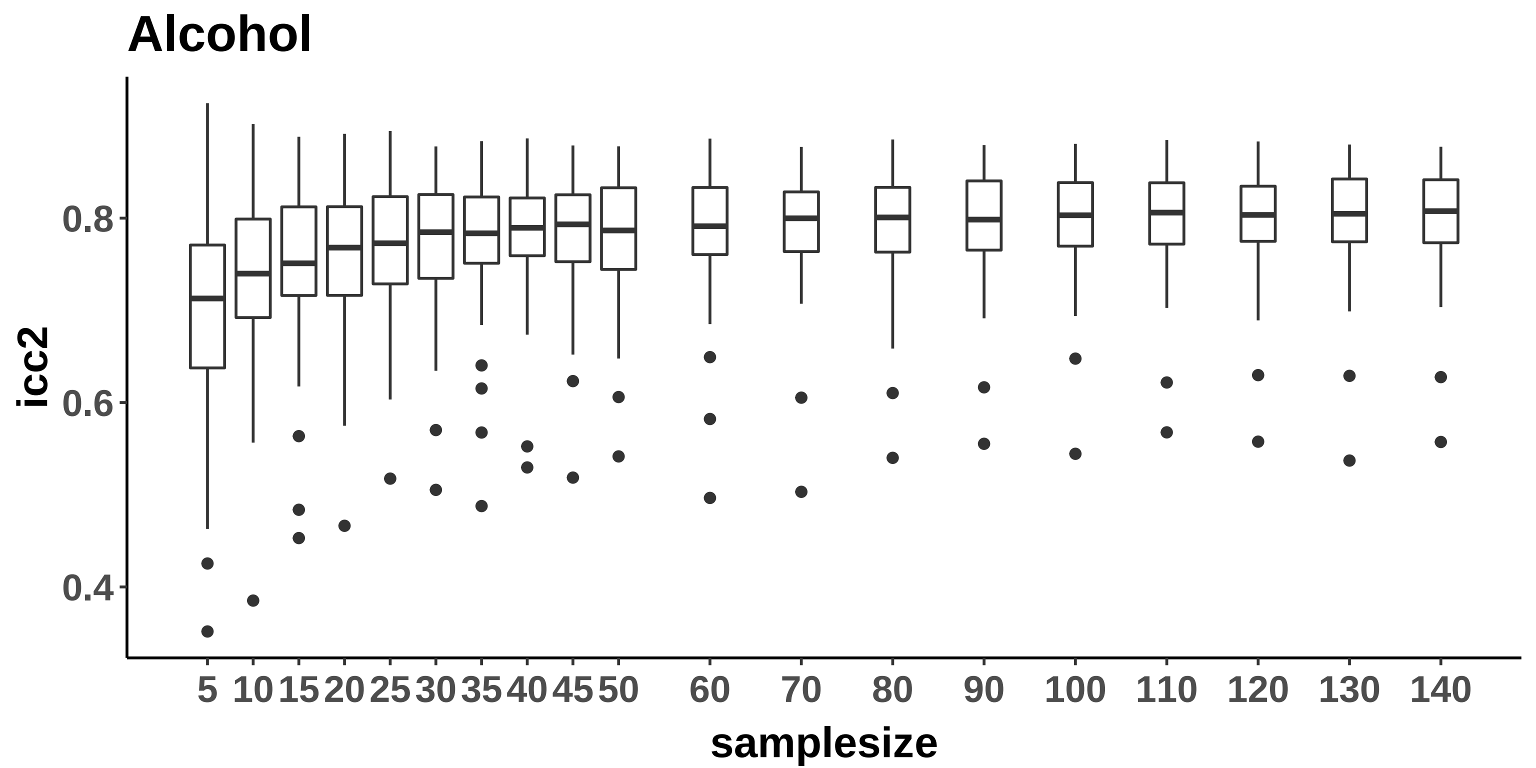
Alcohol
icc2
0.8
0.6
0.4
5 10 15 20 25 30 35 40 45 50 60 70 80 90 100 110 120 130 140
samplesize

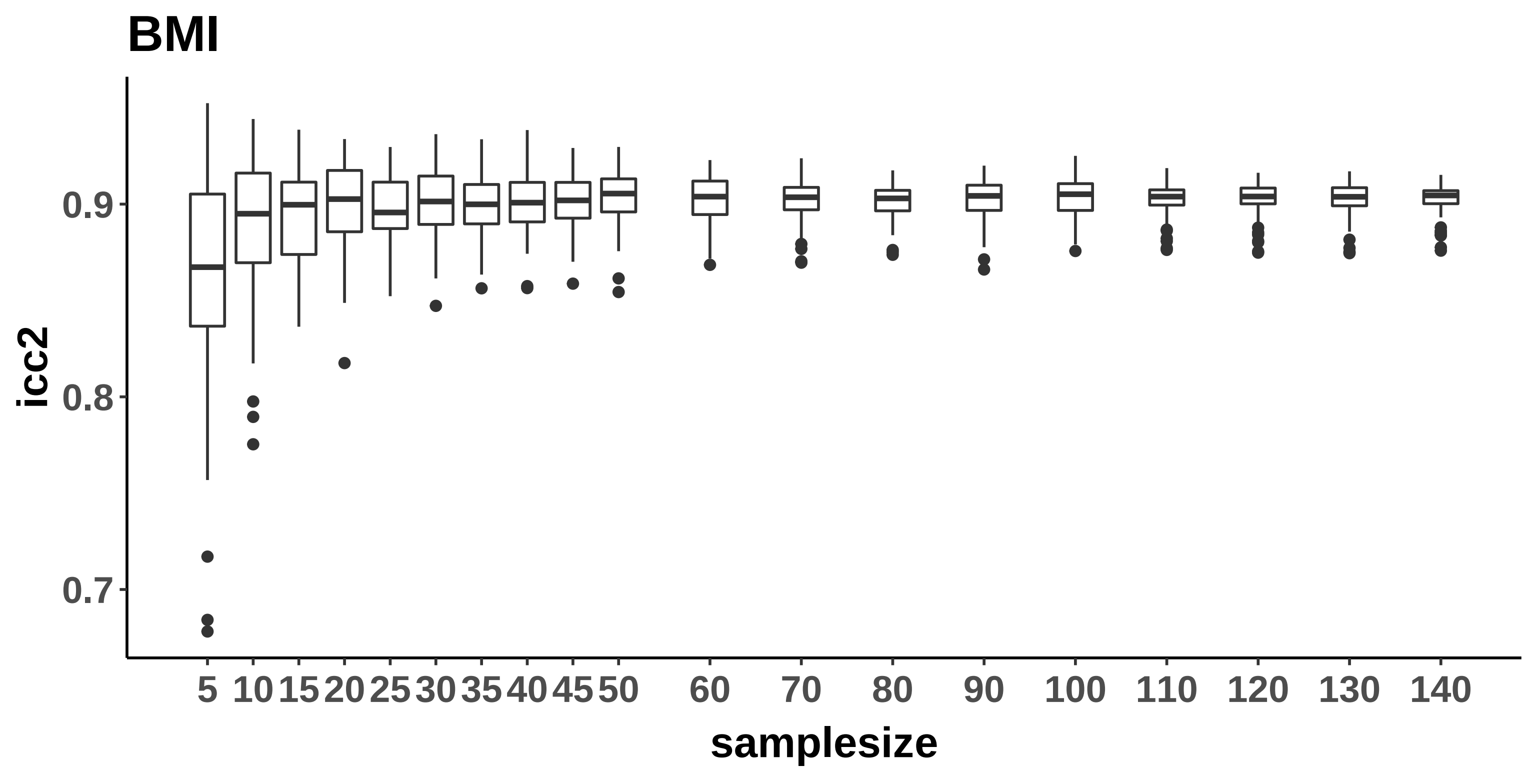

BMI
icc2
0.9
0.8
0.7
5
10
15
20
25
30
35
40
45
50
60
70
80
90
100
110
120
130
140
samplesize

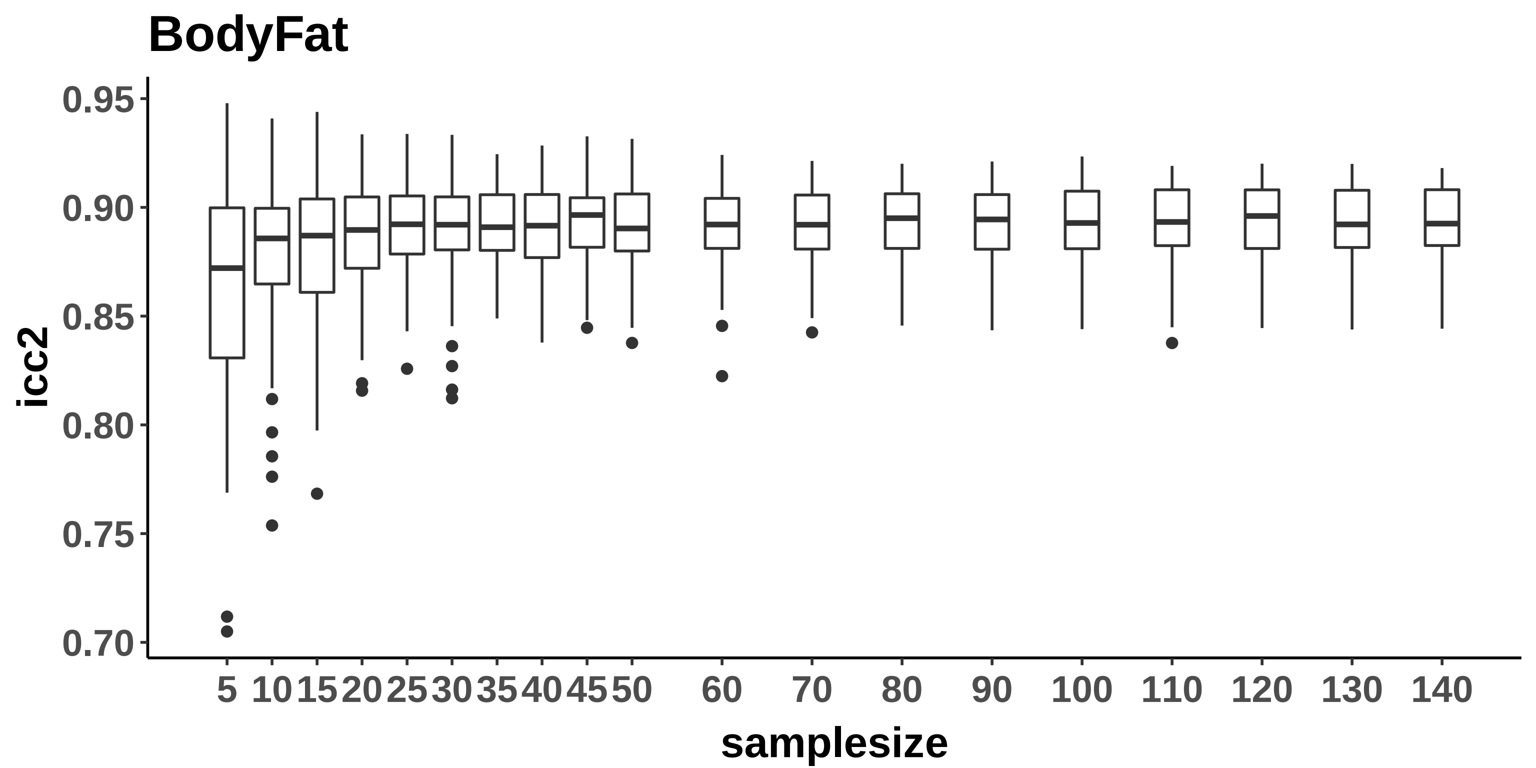
BodyFat
icc2
0.95
0.90
0.85
0.80
0.75
0.70
5 10 15 20 25 30 35 40 45 50 60 70 80 90 100 110 120 130 140
samplesize

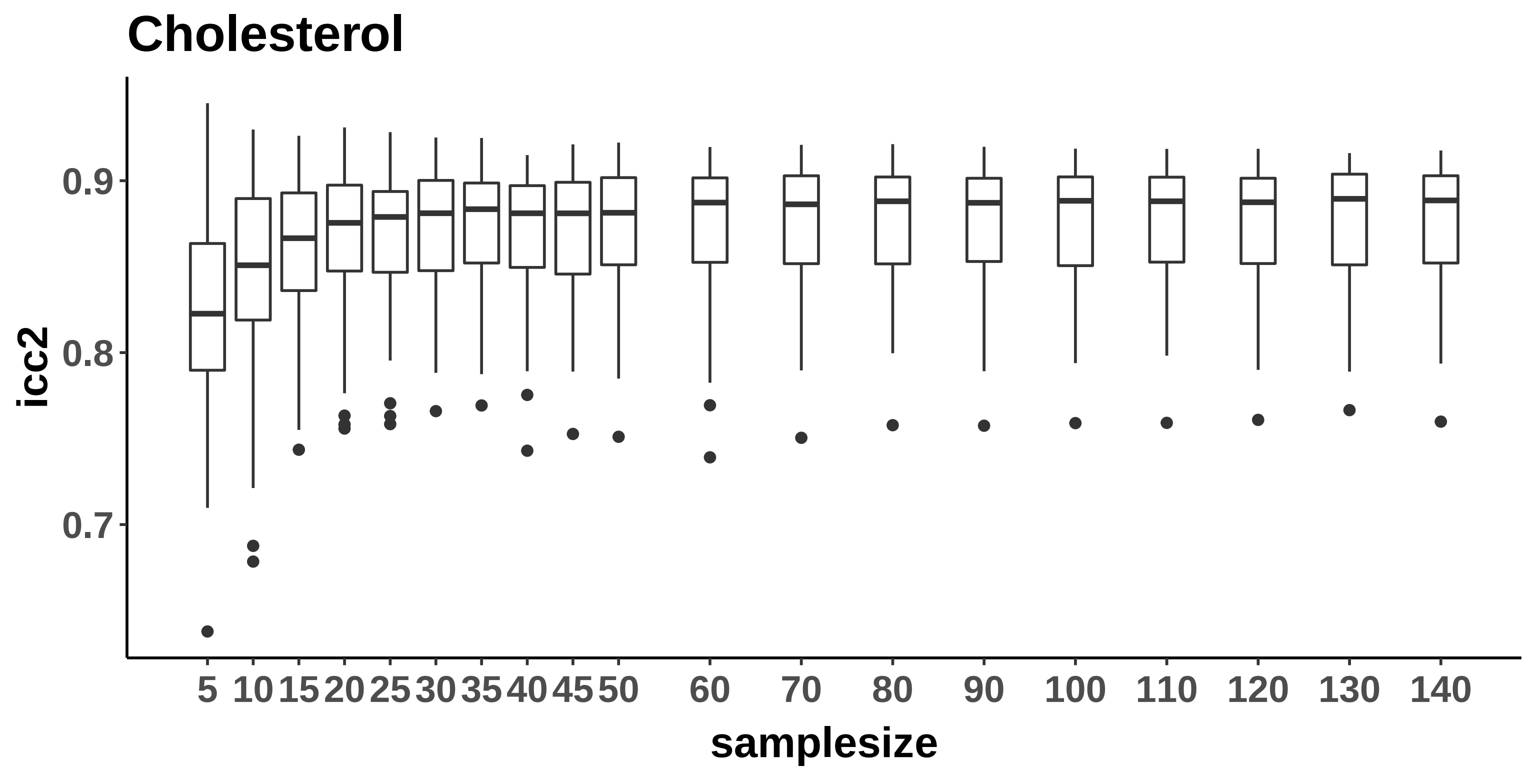

Cholesterol
icc2
0.9
0.8
0.7
5
10
15
20
25
30
35
40
45
50
60
70
80
90
100
110
120
130
140
samplesize

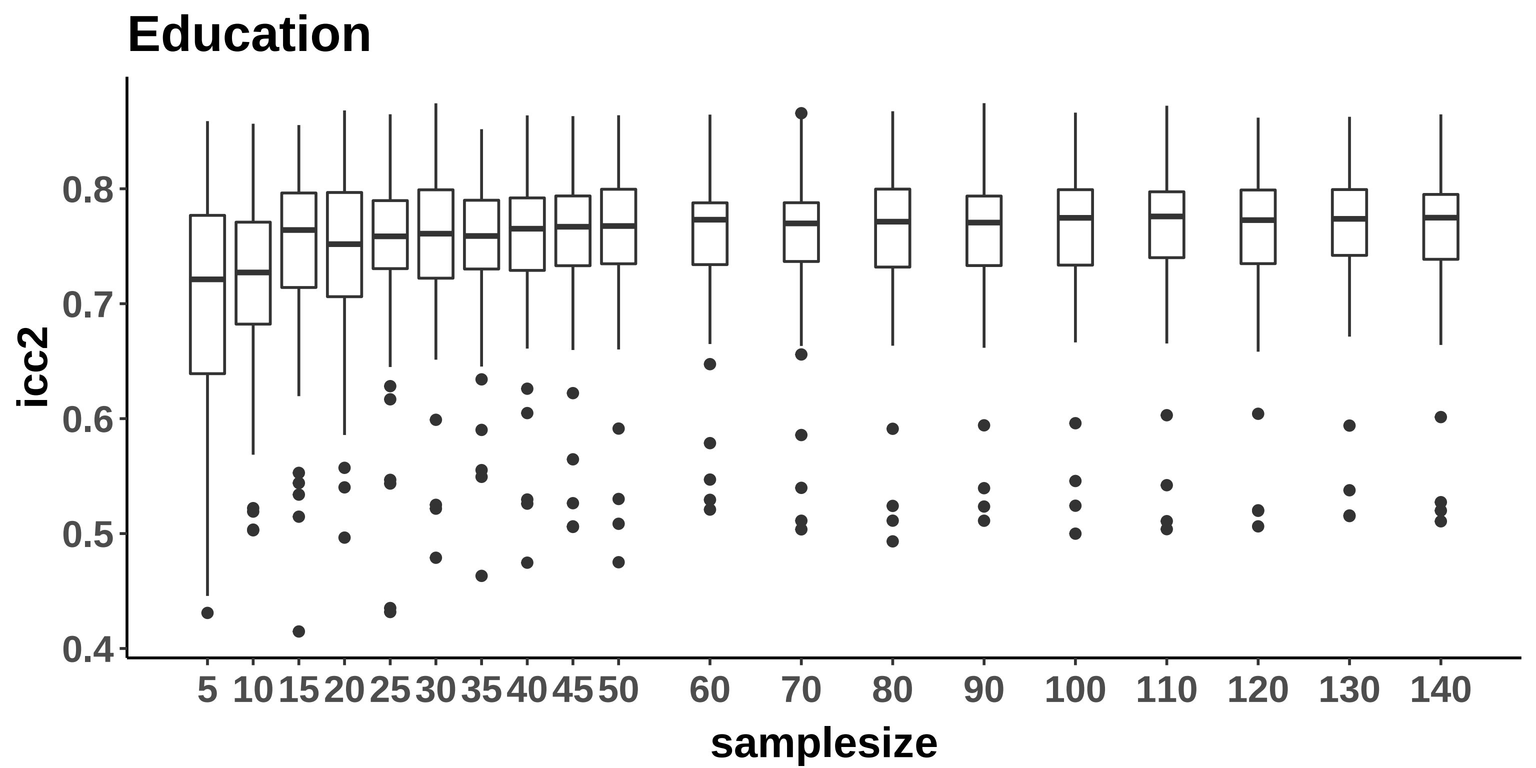
Education
icc2
0.8
0.7
0.6
0.5
0.4
5 10 15 20 25 30 35 40 45 50 60 70 80 90 100 110 120 130 140
samplesize

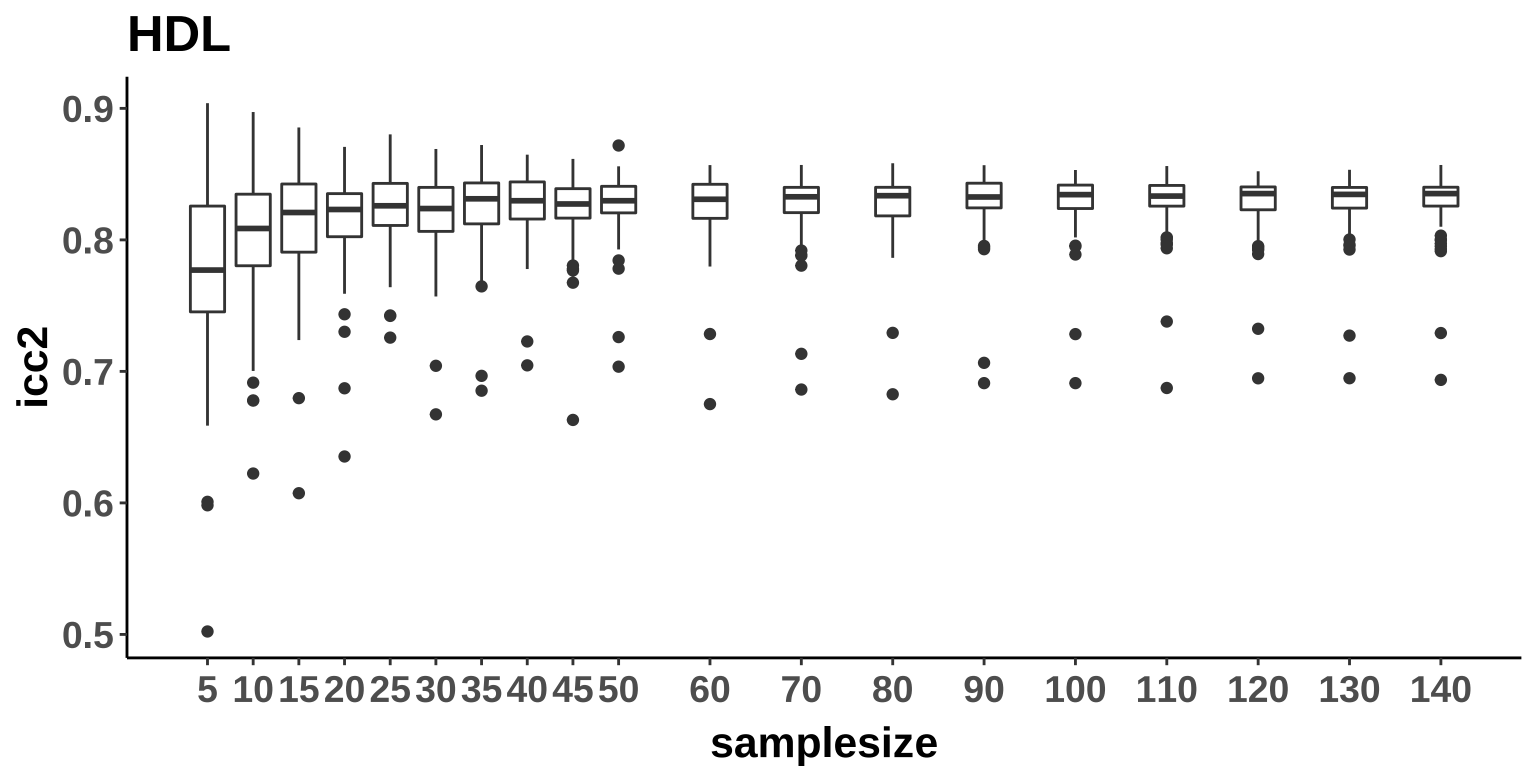
HDL
icc2
0.9
0.8
0.7
0.6
0.5
5 10 15 20 25 30 35 40 45 50 60 70 80 90 100 110 120 130 140
samplesize

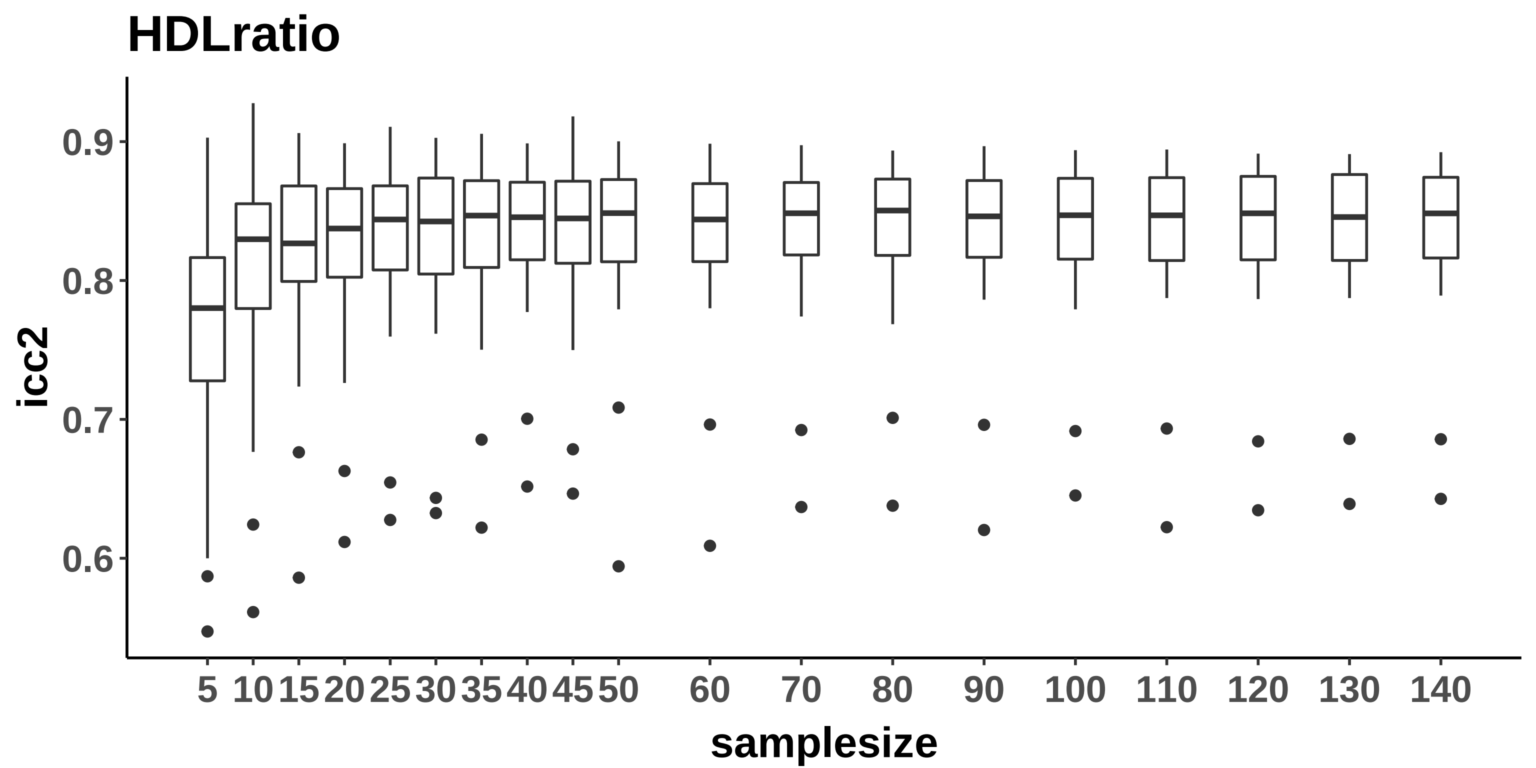
HDLratio
icc2
0.9
0.8
0.7
0.6
5
10
15
20
25
30
35
40
45
50
60
70
80
90
100
110
120
130
140
samplesize

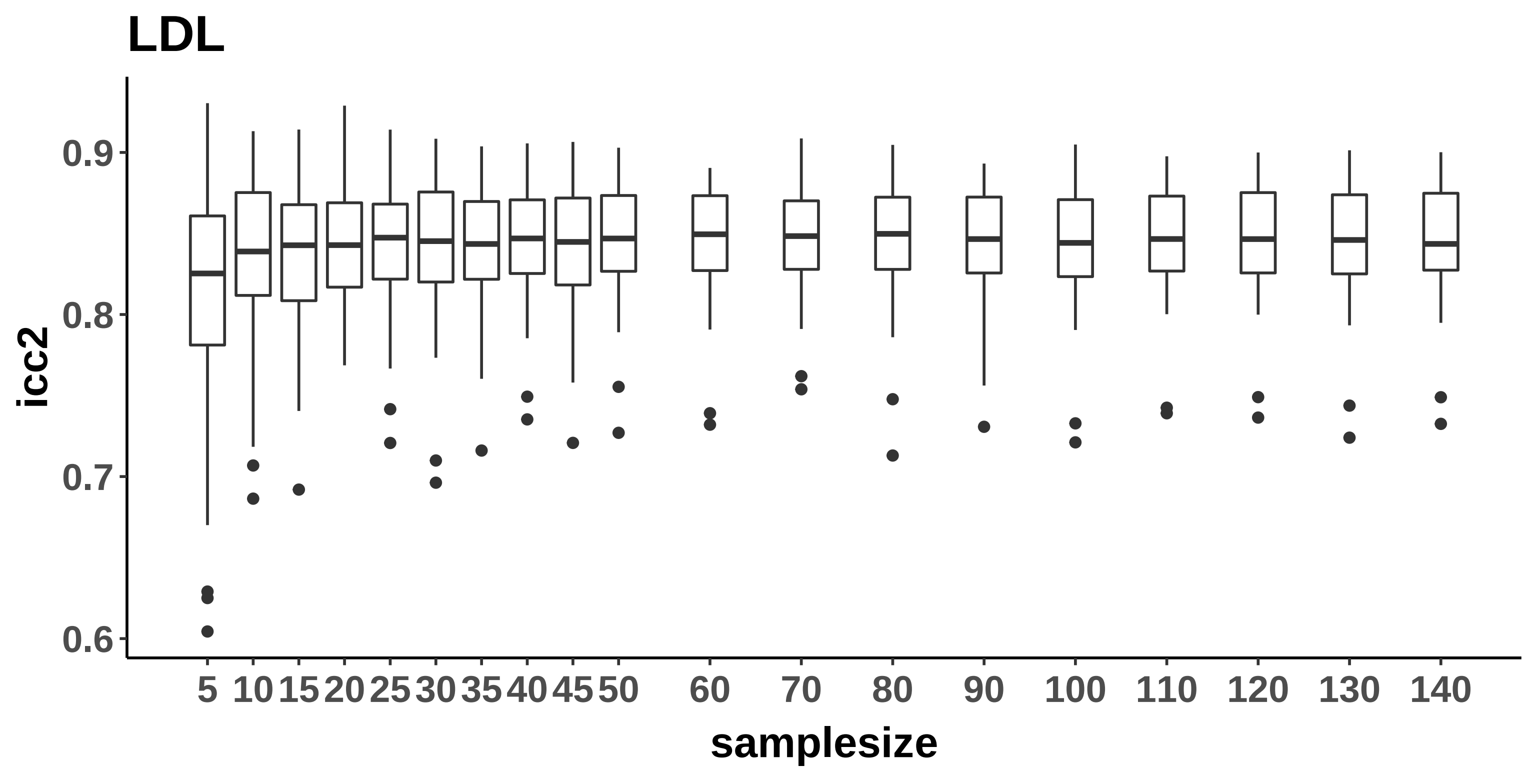

LDL
icc2
0.9
0.8
0.7
0.6
5
10
15
20
25
30
35
40
45
50
60
70
80
90
100
110
120
130
140
samplesize

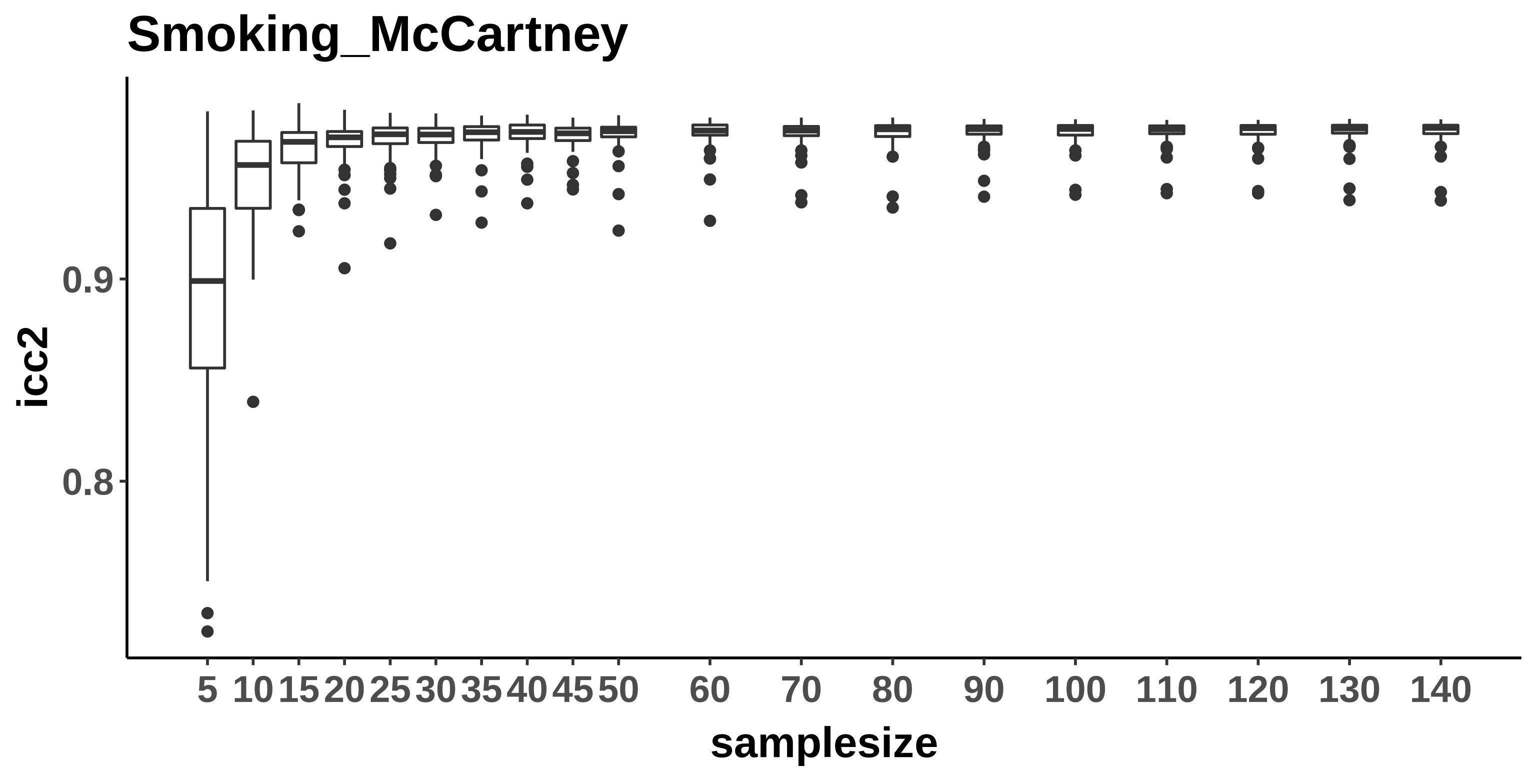

Smoking_McCartney
icc2
0.9
0.8
5
10
15
20
25
30
35
40
45
50
60
70
80
90
100
110
120
130
140
samplesize

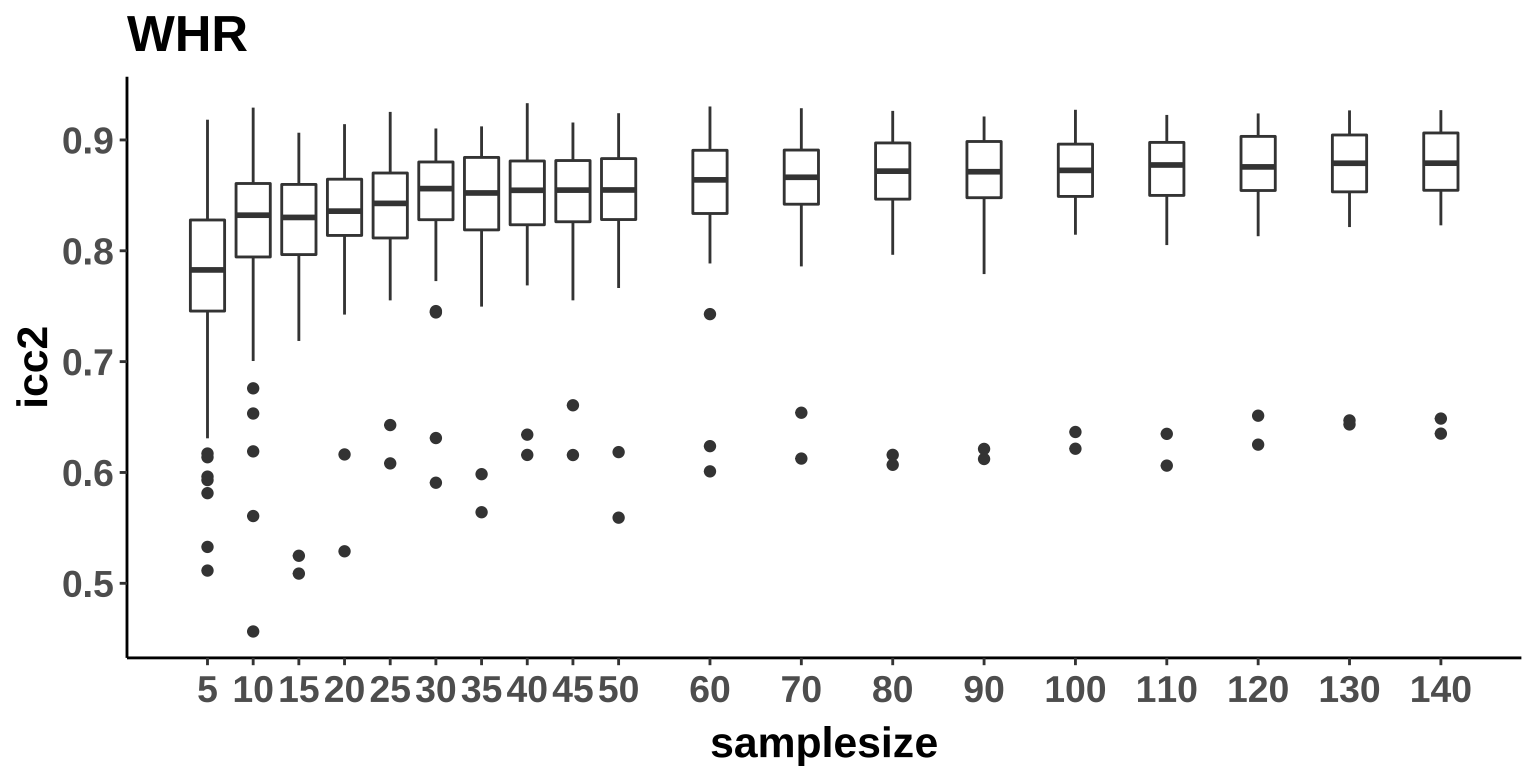

WHR
icc2
0.9
0.8
0.7
0.6
0.5
5
10
15
20
25
30
35
40
45
50
60
70
80
90
100
110
120
130
140
samplesize

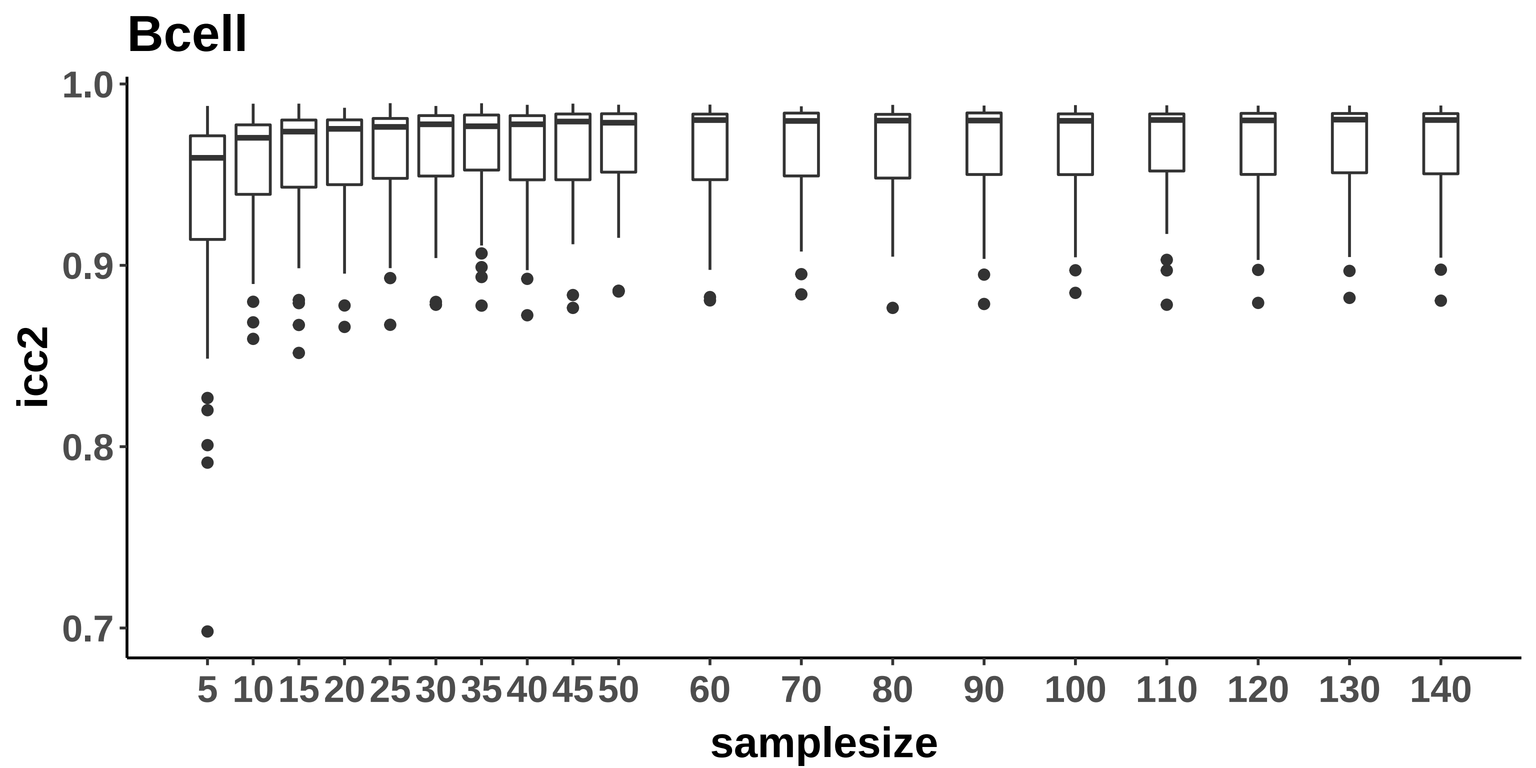
Bcell
icc2
1.0
0.9
0.8
0.7
5
10
15
20
25
30
35
40
45
50
60
70
80
90
100
110
120
130
140
samplesize

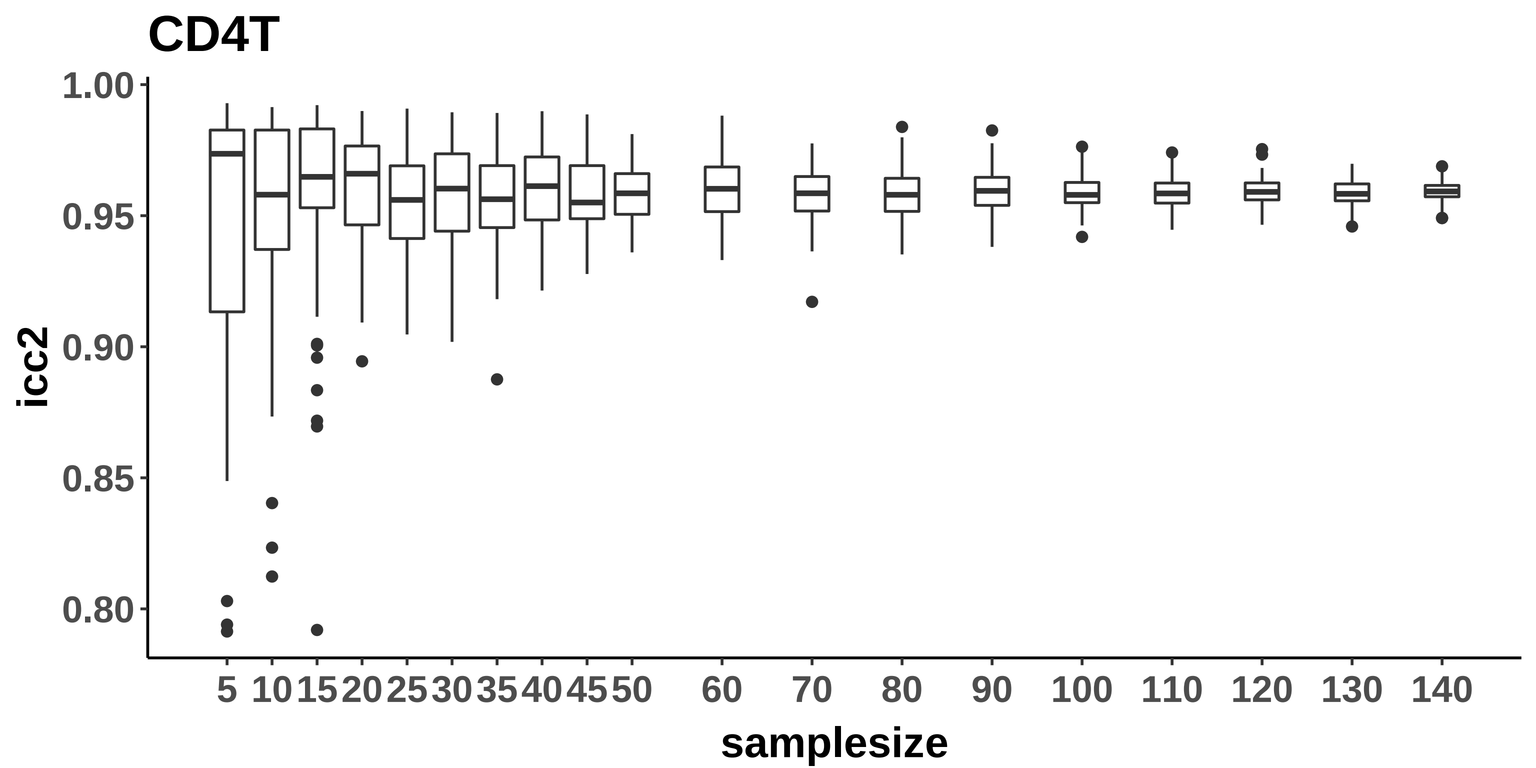

CD4T
icc2
1.00
0.95
0.90
0.85
0.80
5
10
15
20
25
30
35
40
45
50
60
70
80
90
100
110
120
130
140
samplesize

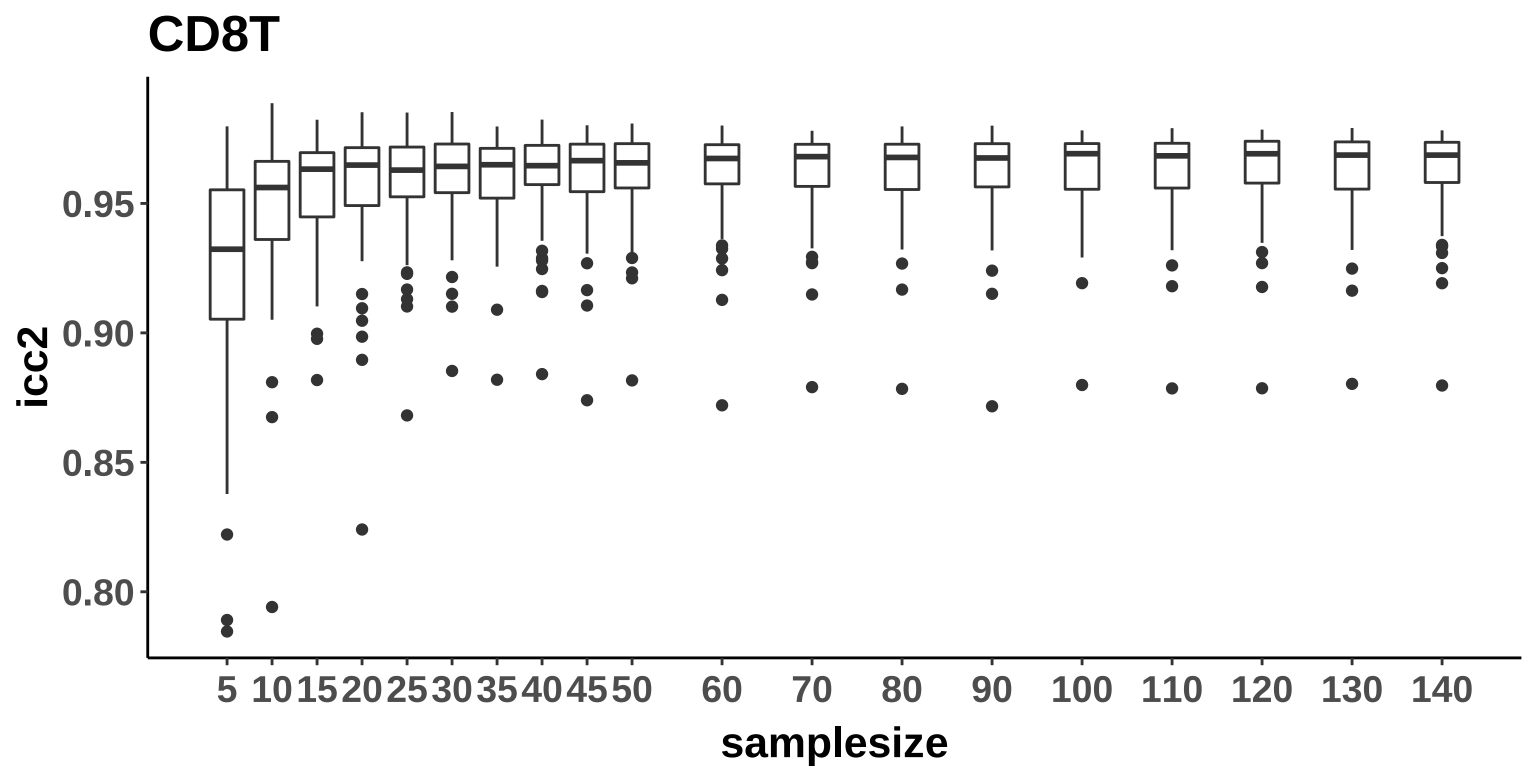

CD8T
icc2
0.95
0.90
0.85
0.80
5 10 15 20 25 30 35 40 45 50 60 70 80 90 100 110 120 130 140
samplesize

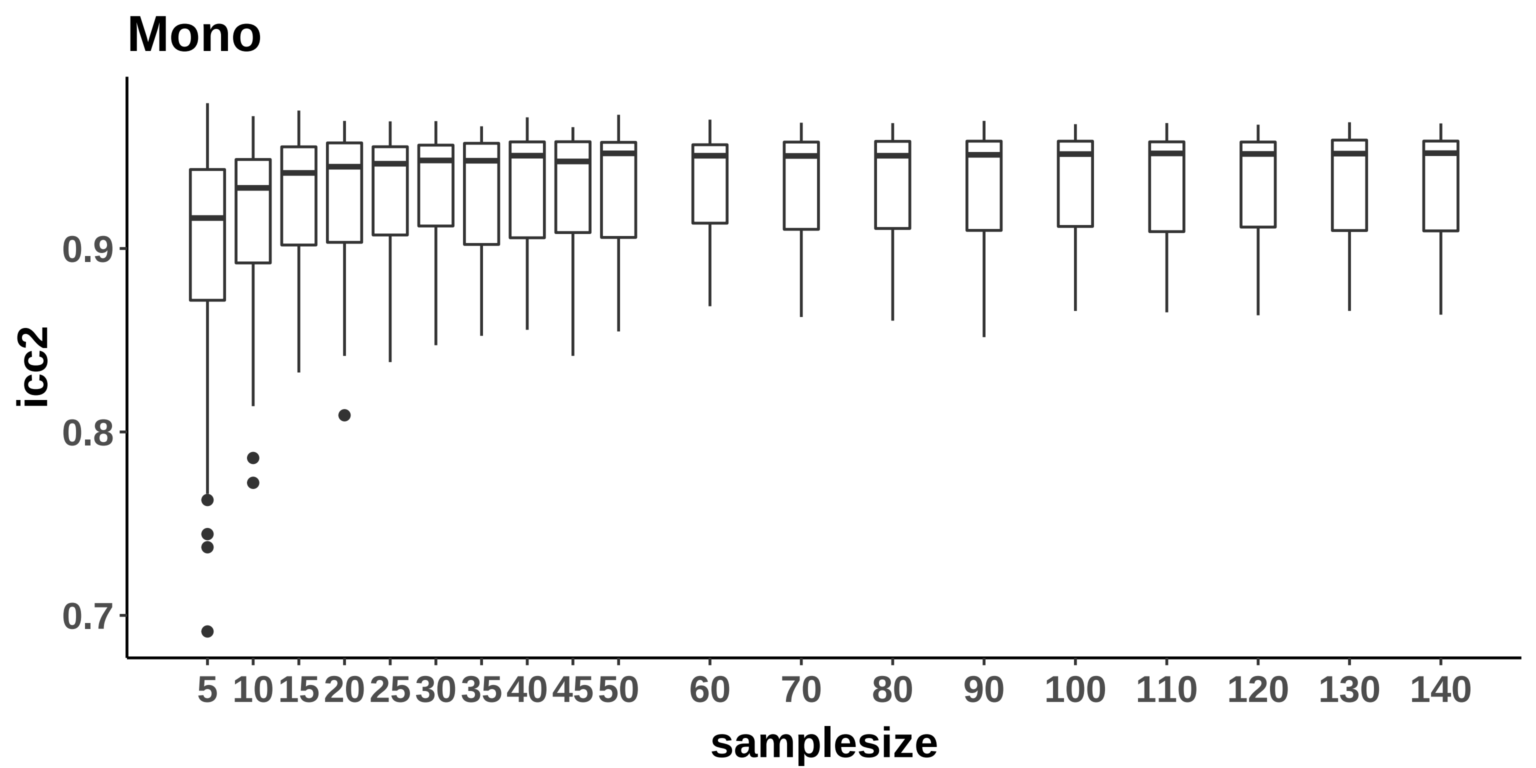
Mono
icc2
0.9
0.8
0.7
5
10
15
20
25
30
35
40
45
50
60
70
80
90
100
110
120
130
140
samplesize

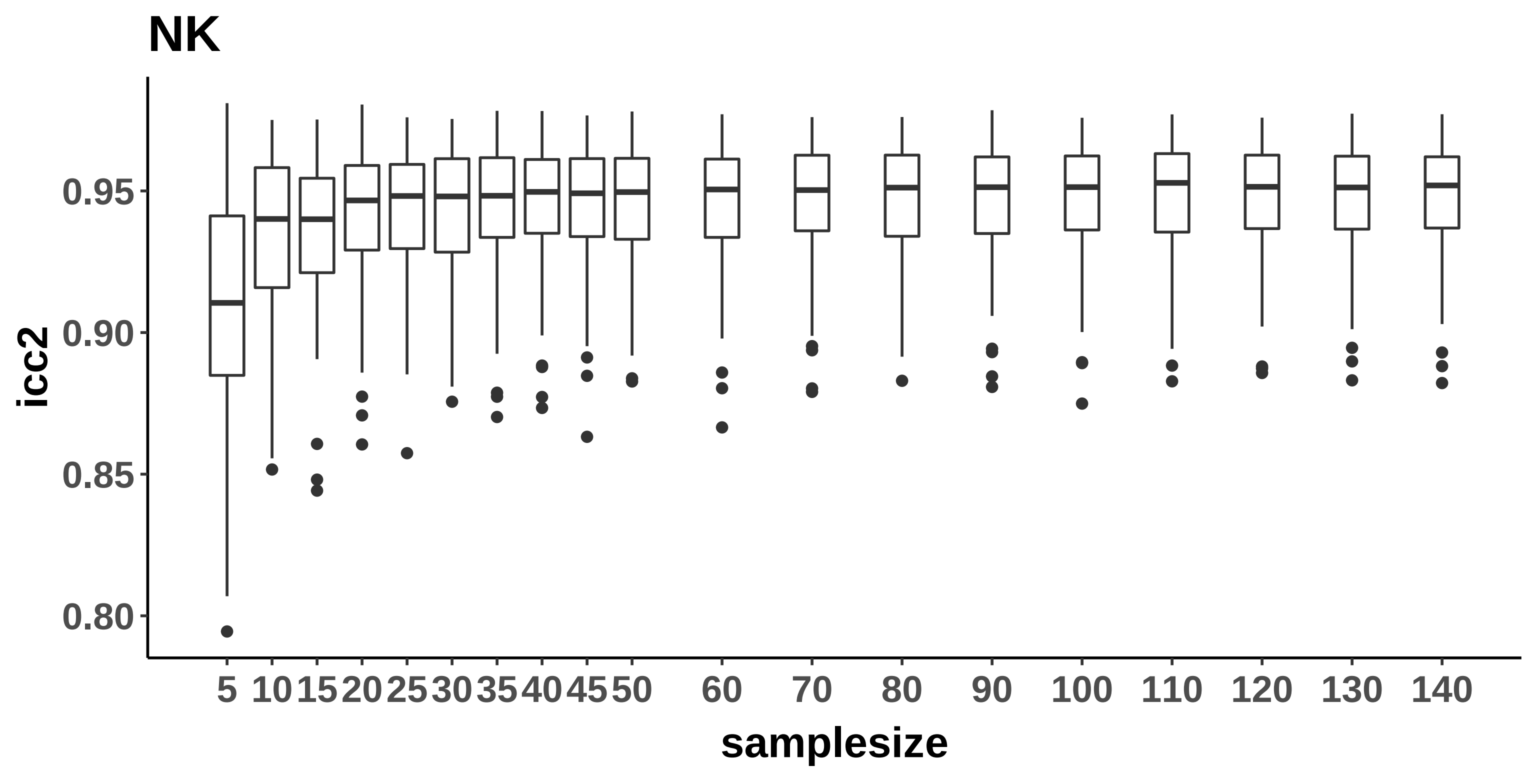
NK
icc2
0.95
0.90
0.85
0.80
5 10 15 20 25 30 35 40 45 50 60 70 80 90 100 110 120 130 140
samplesize

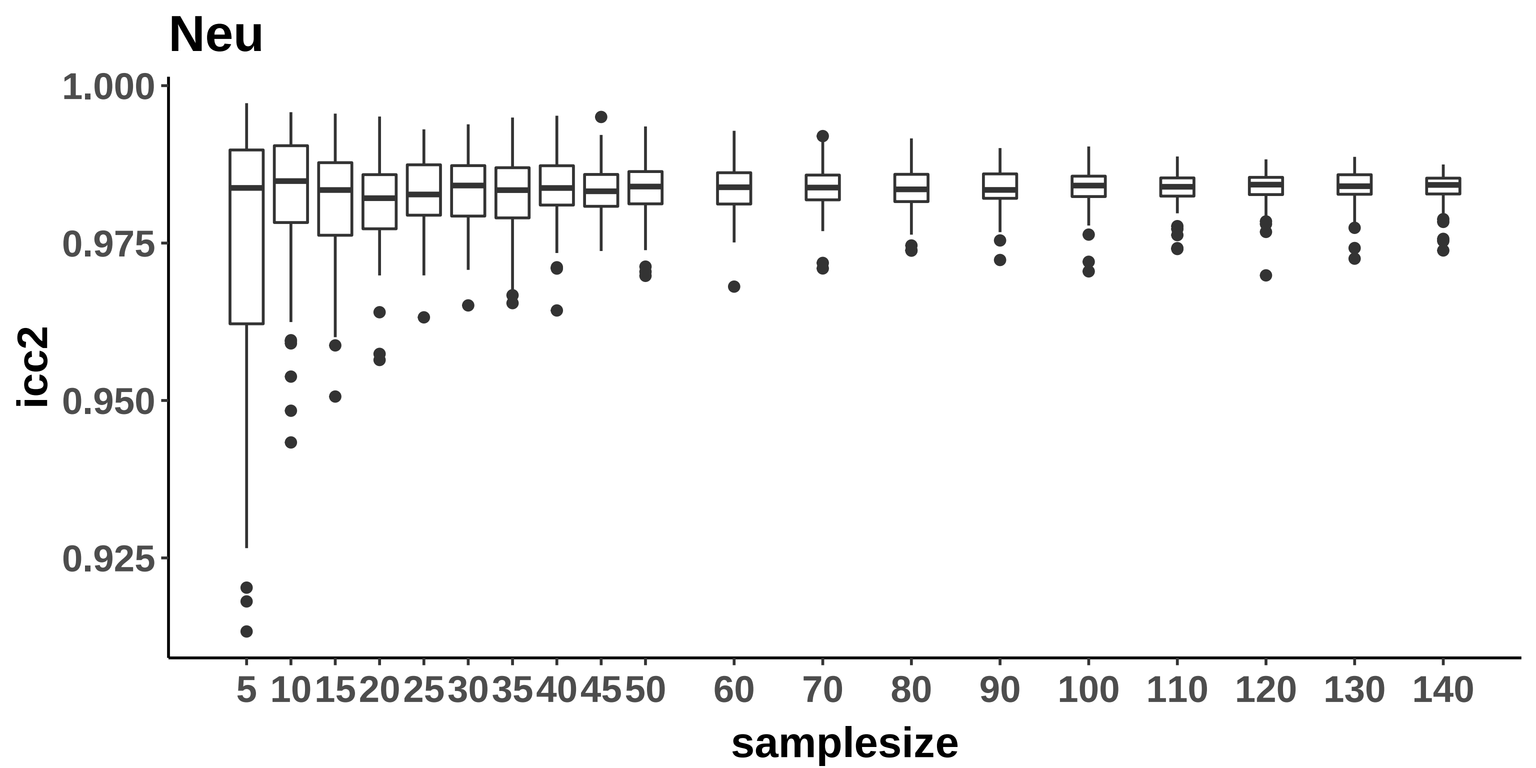
Neu
icc2
1.000
0.975
0.950
0.925
5
10
15
20
25
30
35
40
45
50
60
70
80
90
100
110
120
130
140
samplesize

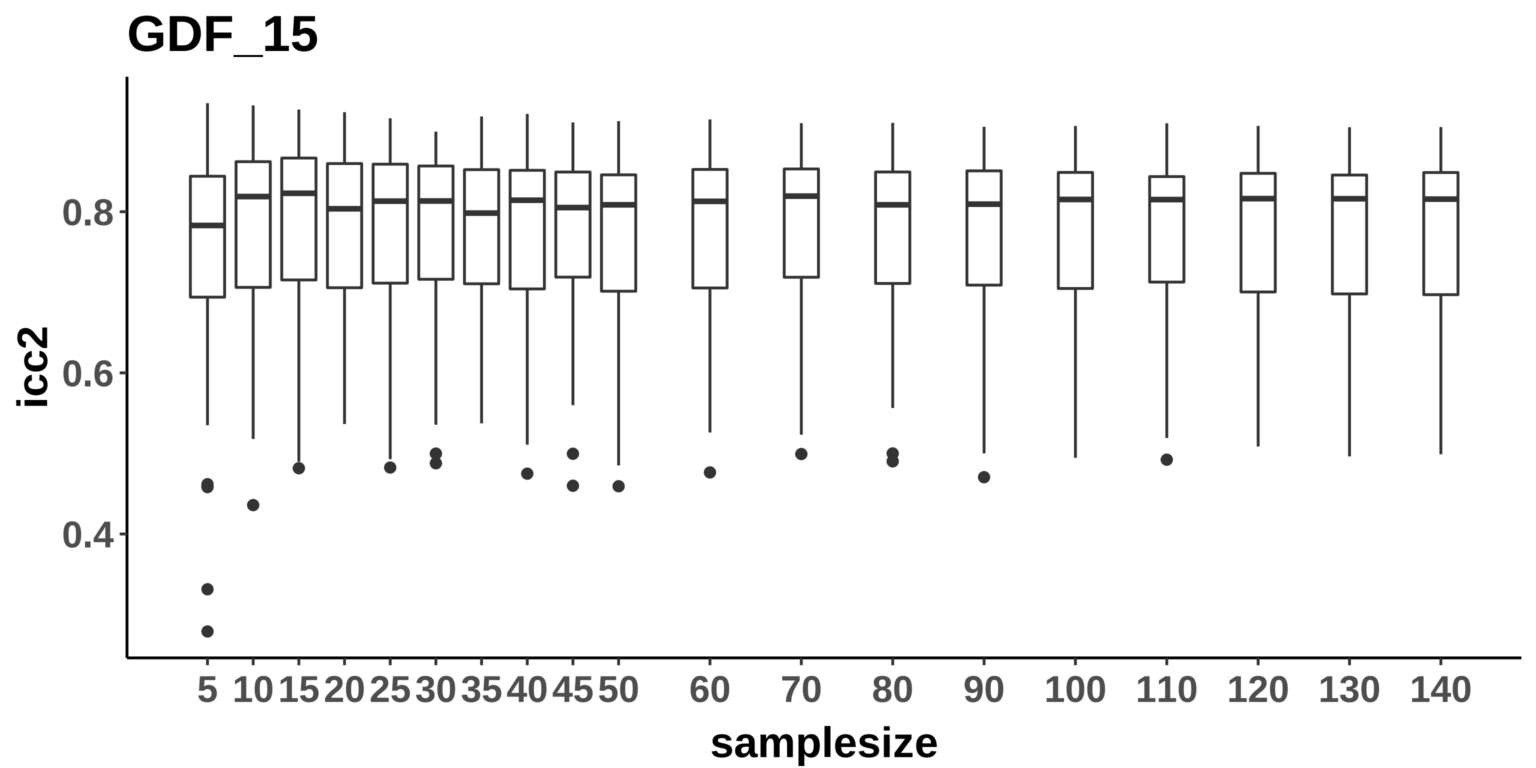

GDF_15
icc2
0.8
0.6
0.4
5
10
15
20
25
30
35
40
45
50
60
70
80
90
100
110
120
130
140
samplesize

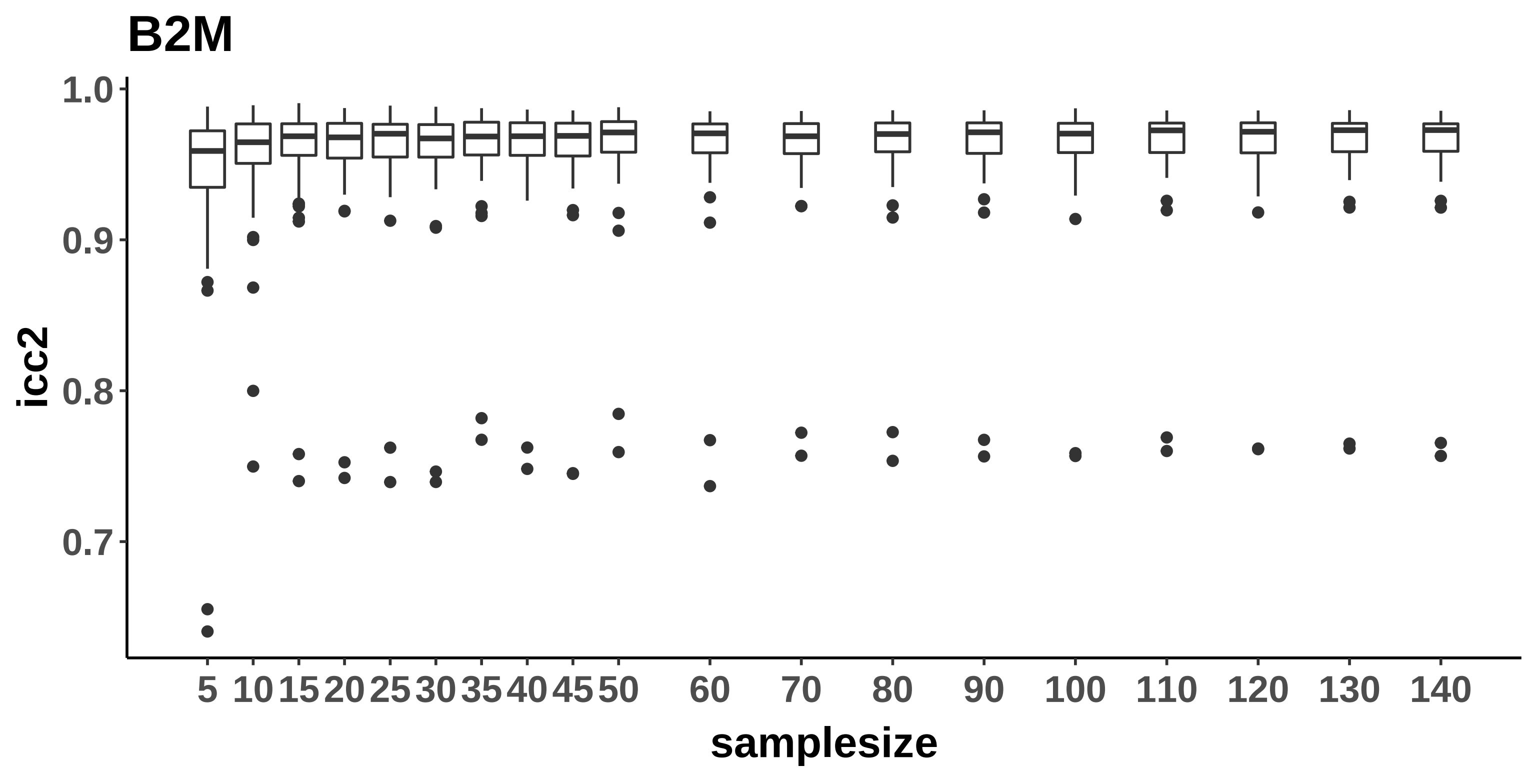
B2M
icc2
1.0
0.9
0.8
0.7
5
10
15
20
25
30
35
40
45
50
60
70
80
90
100
110
120
130
140
samplesize

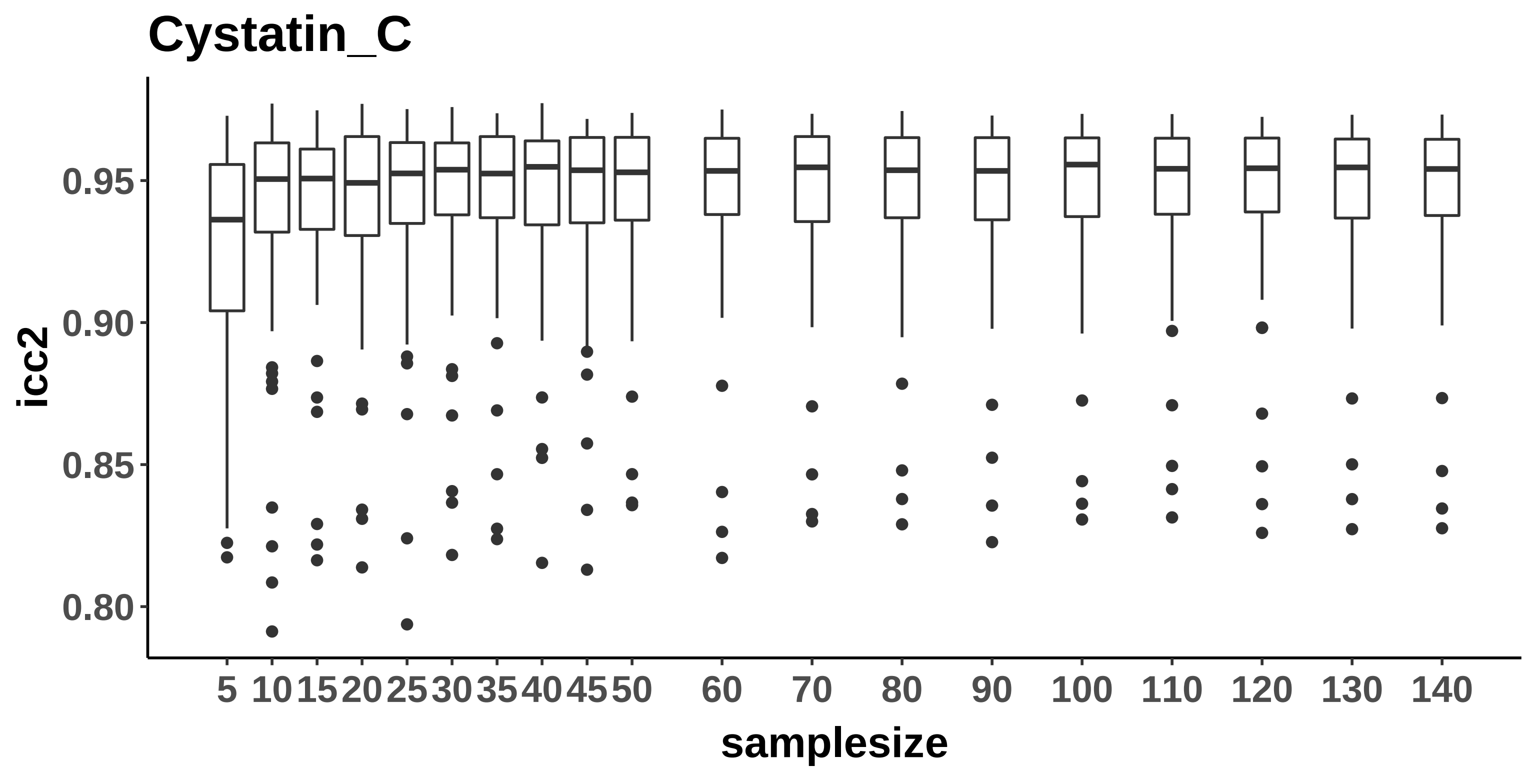

Cystatin_C
icc2
0.95
0.90
0.85
0.80
5 10 15 20 25 30 35 40 45 50 60 70 80 90 100 110 120 130 140
samplesize

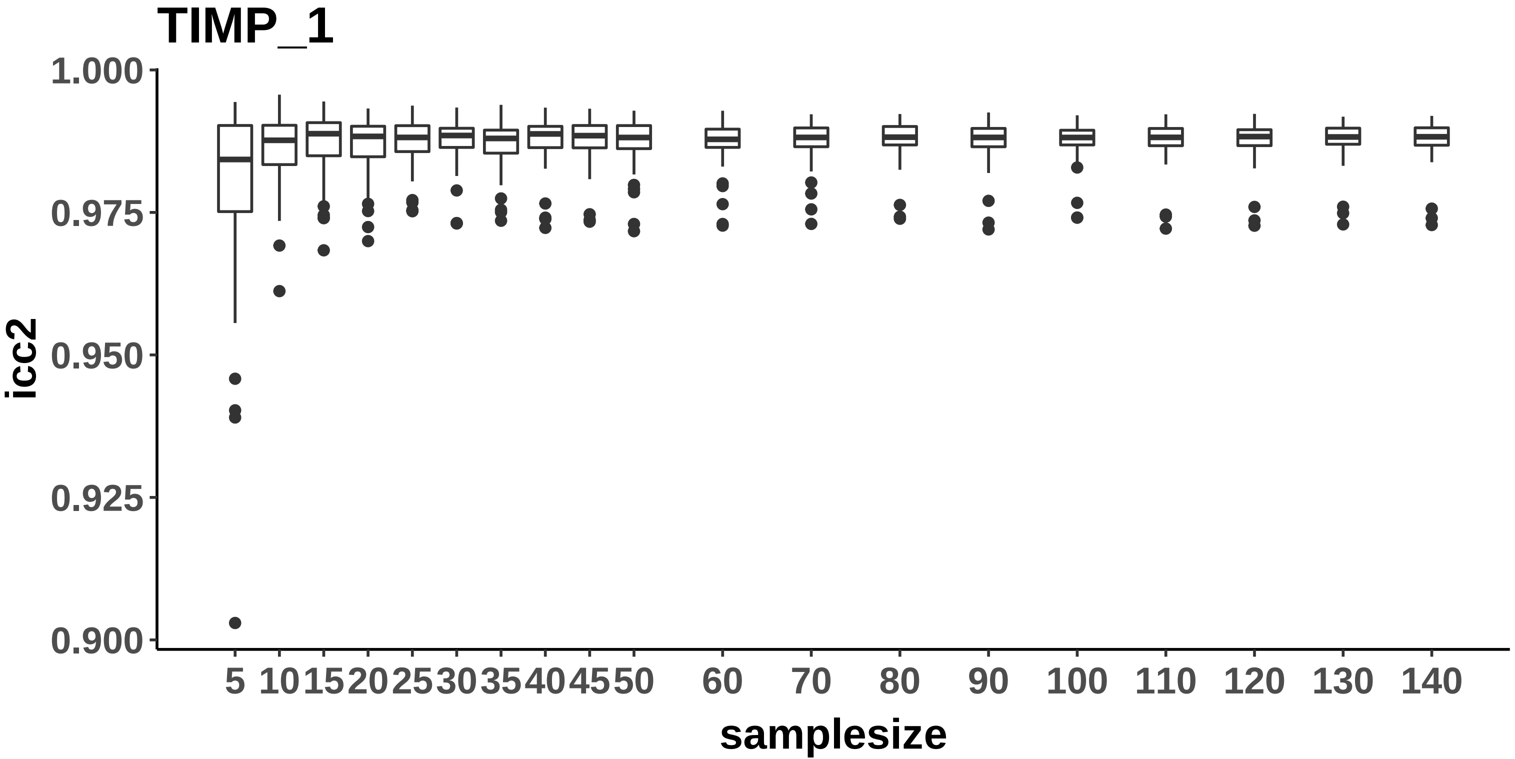

TIMP_1
icc2
1.000
0.975
0.950
0.925
0.900
5 10 15 20 25 30 35 40 45 50 60 70 80 90 100 110 120 130 140
samplesize

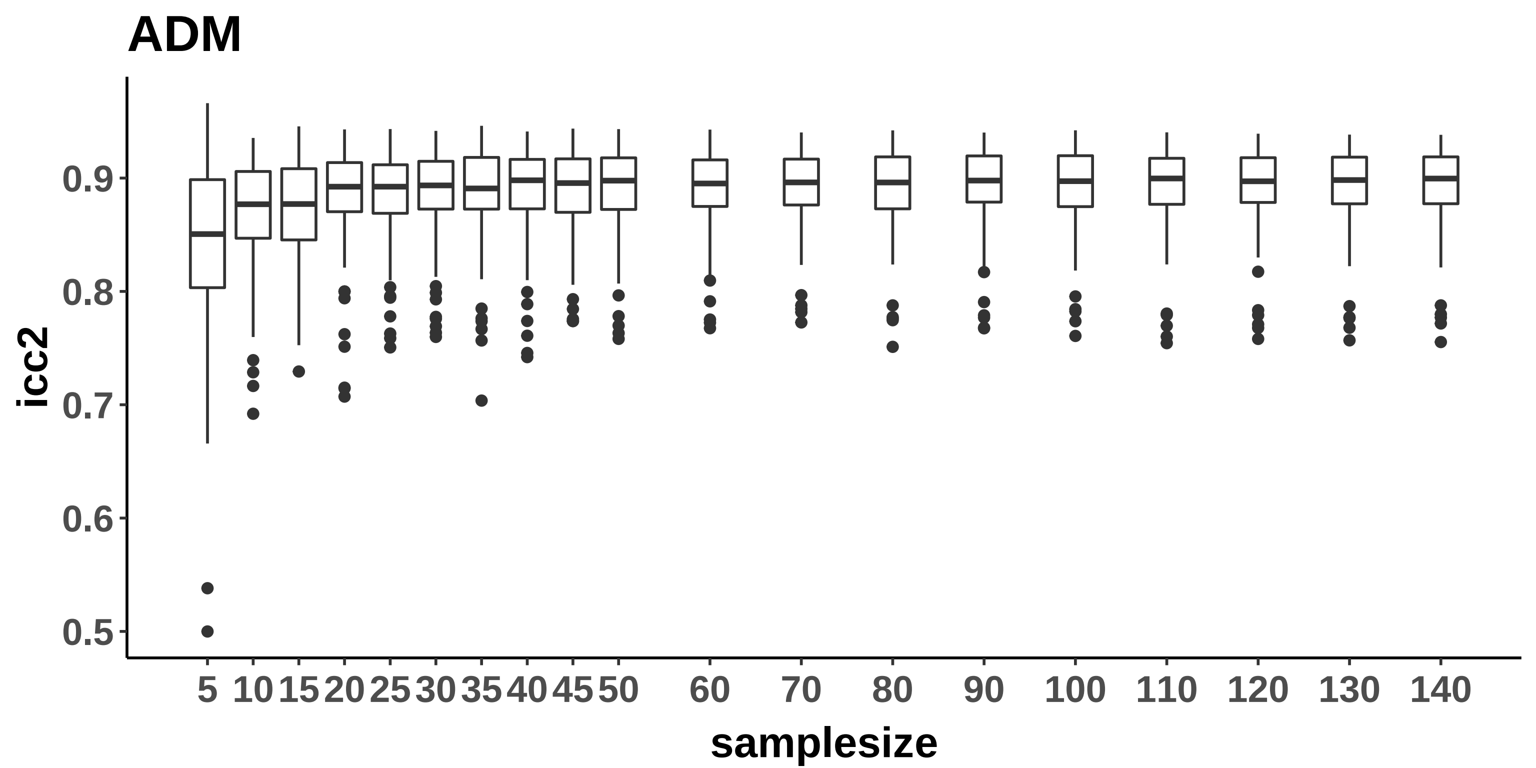
ADM
icc2
0.5
0.6
0.7
0.8
0.9
5
10
15
20
25
30
35
40
45
50
60
70
80
90
100
110
120
130
140
samplesize

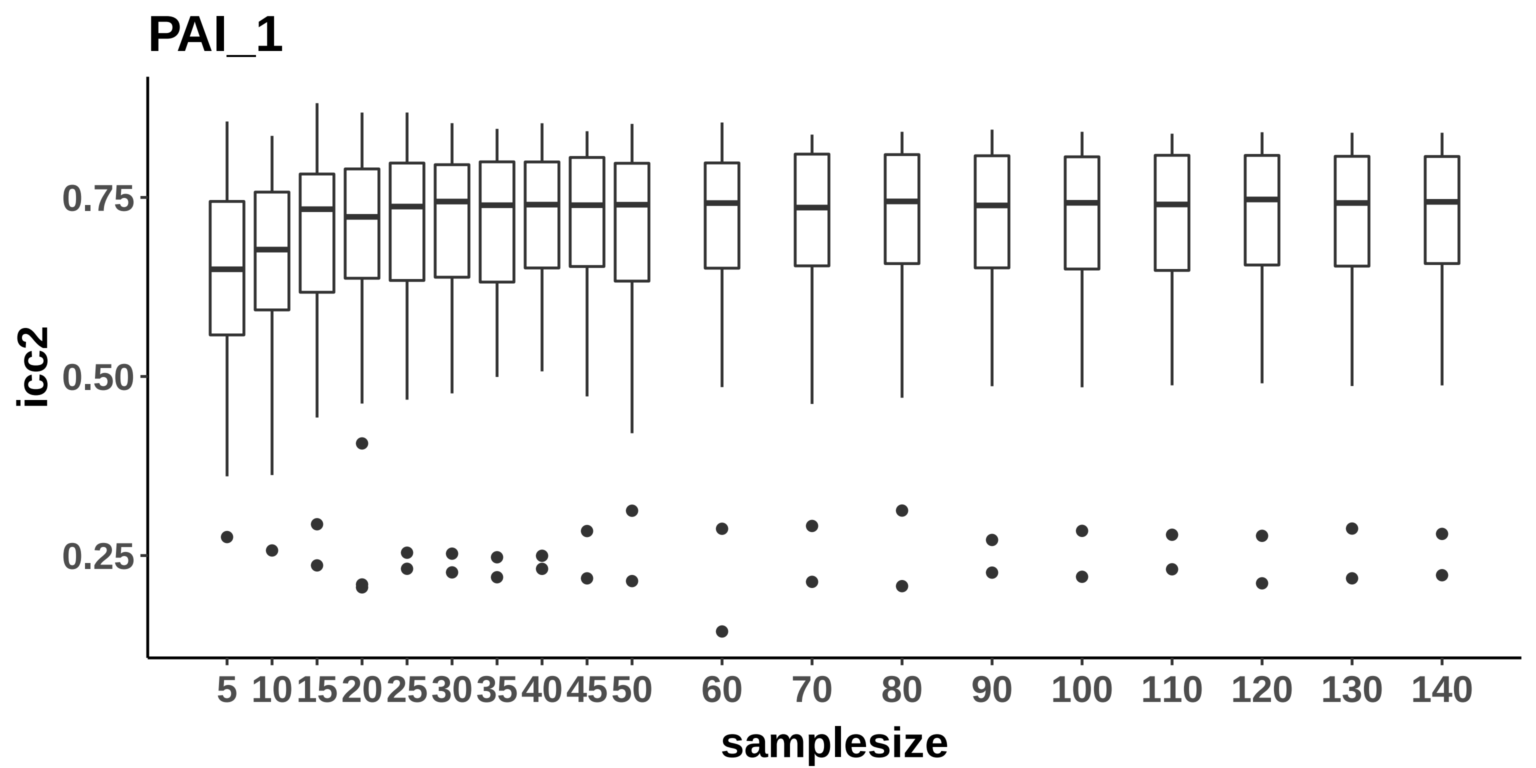
PAI_1
icc2
0.75
0.50
0.25
5
10
15
20
25
30
35
40
45
50
60
70
80
90
100
110
120
130
140
samplesize

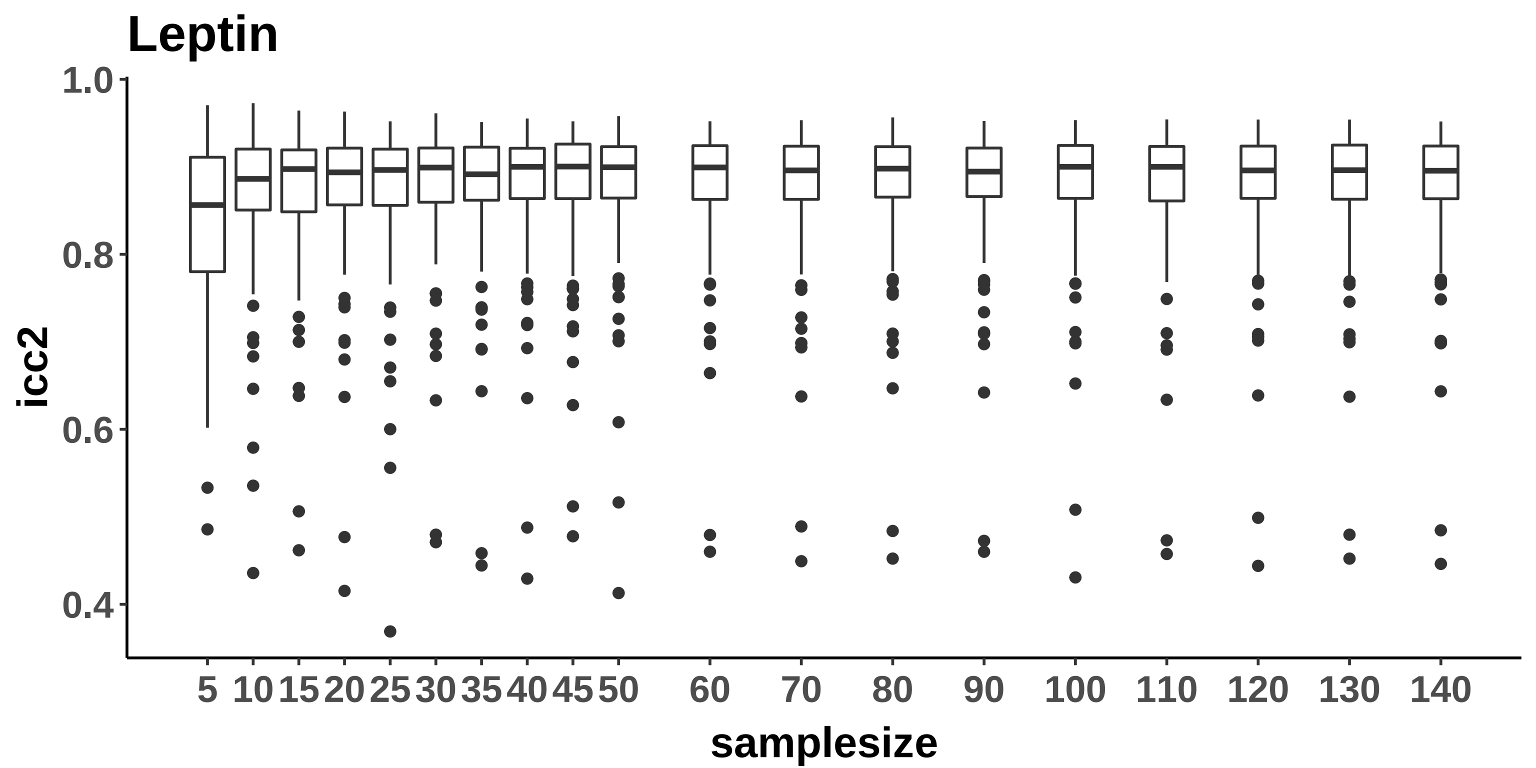

Leptin
1.0
0.8
0.6
0.4
icc2
5
10
15
20
25
30
35
40
45
50
60
70
80
90
100
110
120
130
140
samplesize

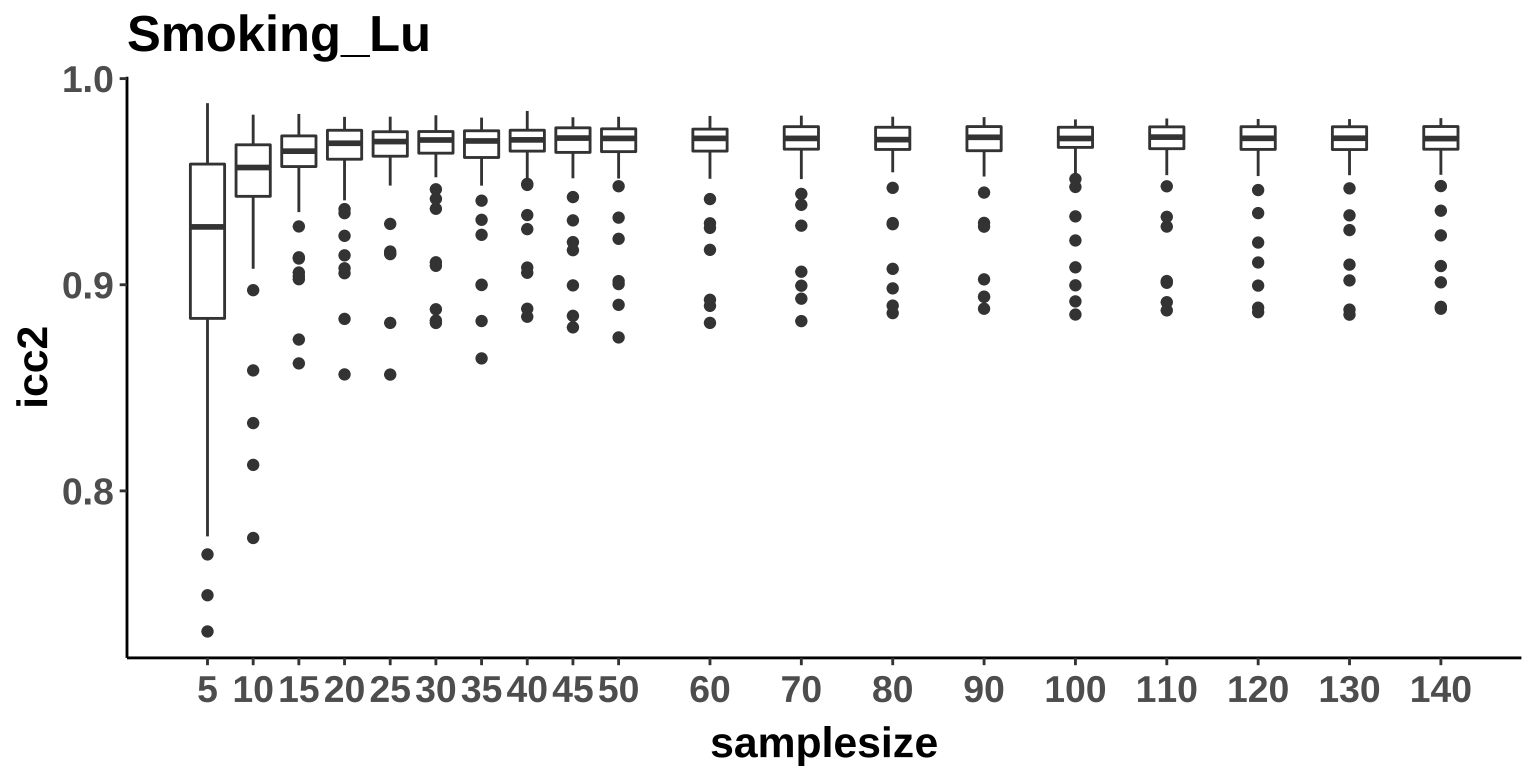

Smoking_Lu
1.0
0.9
0.8
icc2
5
10
15
20
25
30
35
40
45
50
60
70
80
90
100
110
120
130
140
samplesize

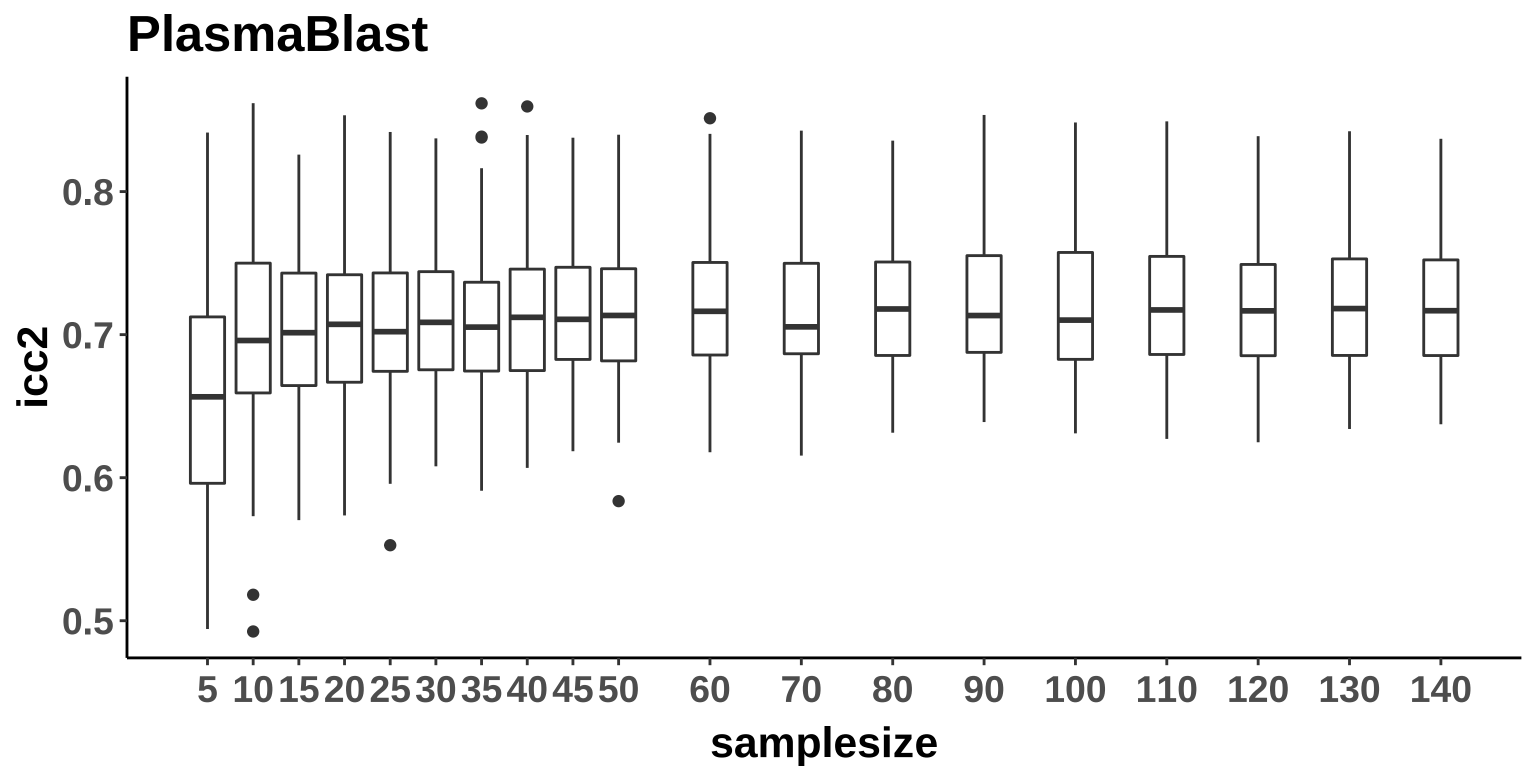

PlasmaBlast
icc2
0.8
0.7
0.6
0.5
5
10
15
20
25
30
35
40
45
50
60
70
80
90
100
110
120
130
140
samplesize

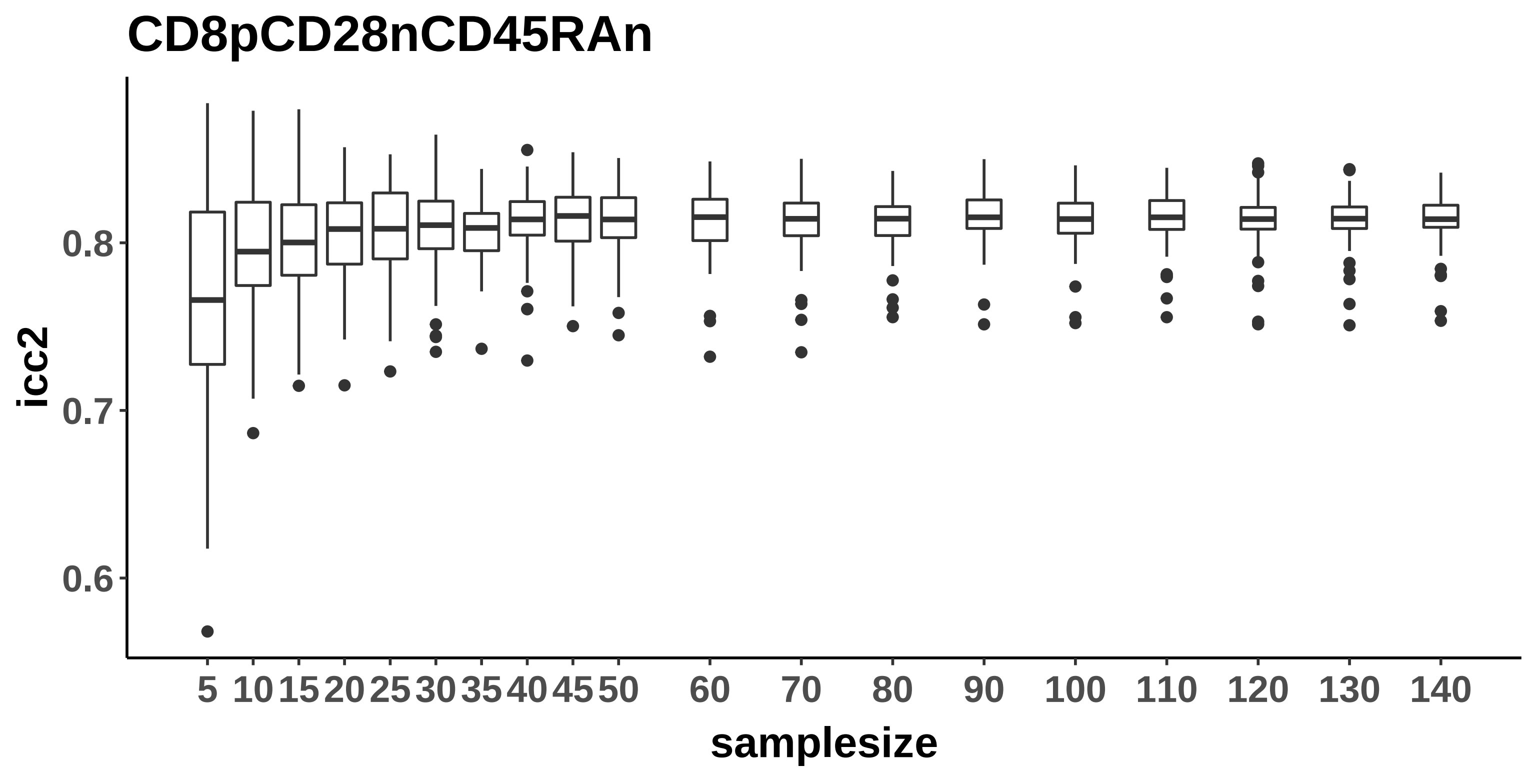
CD8pCD28nCD45RAn
icc2
0.8
0.7
0.6
samplesize
5
10
15
20
25
30
35
40
45
50
60
70
80
90
100
110
120
130
140

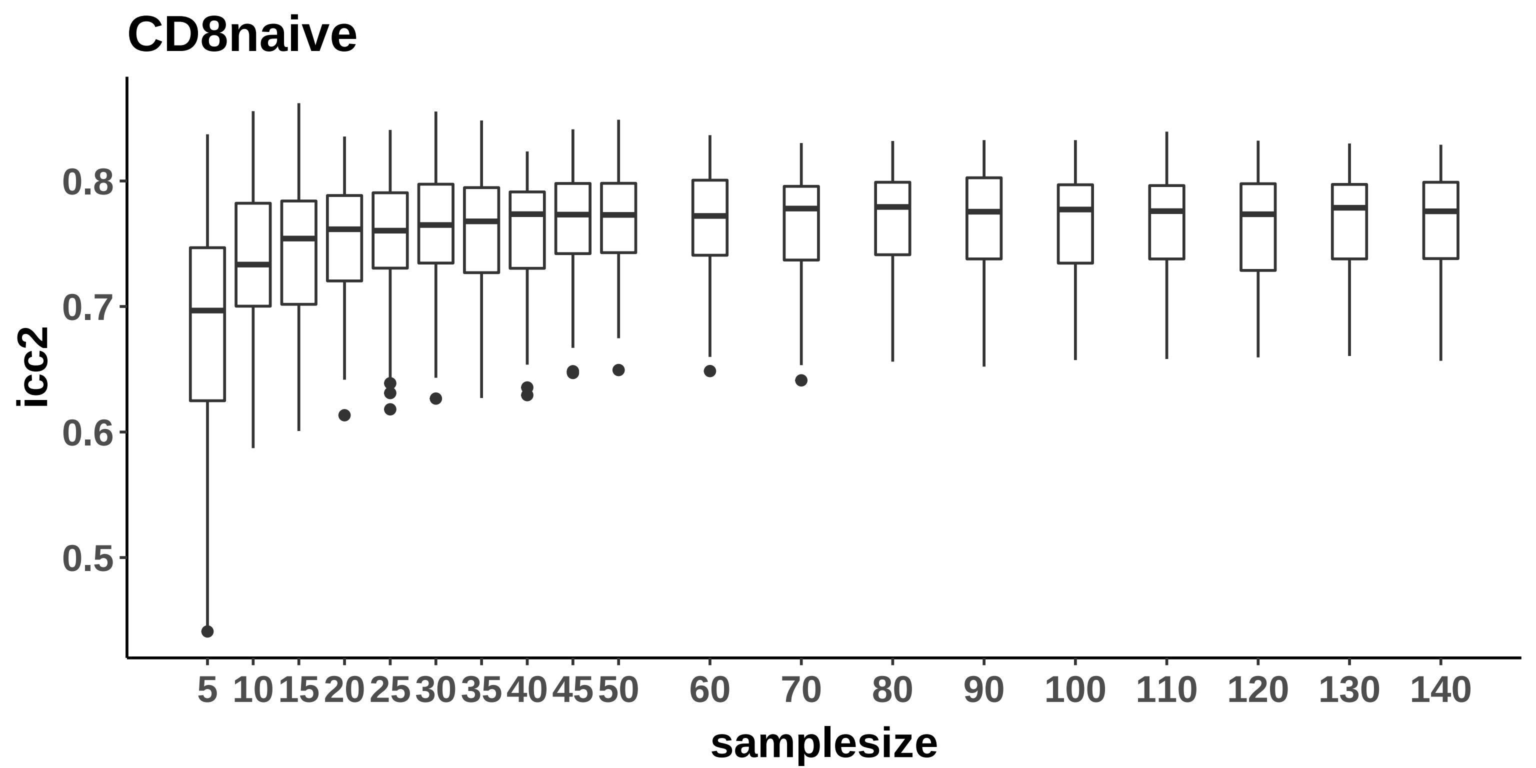
CD8naive
icc2
0.8
0.7
0.6
0.5
5
10
15
20
25
30
35
40
45
50
60
70
80
90
100
110
120
130
140
samplesize

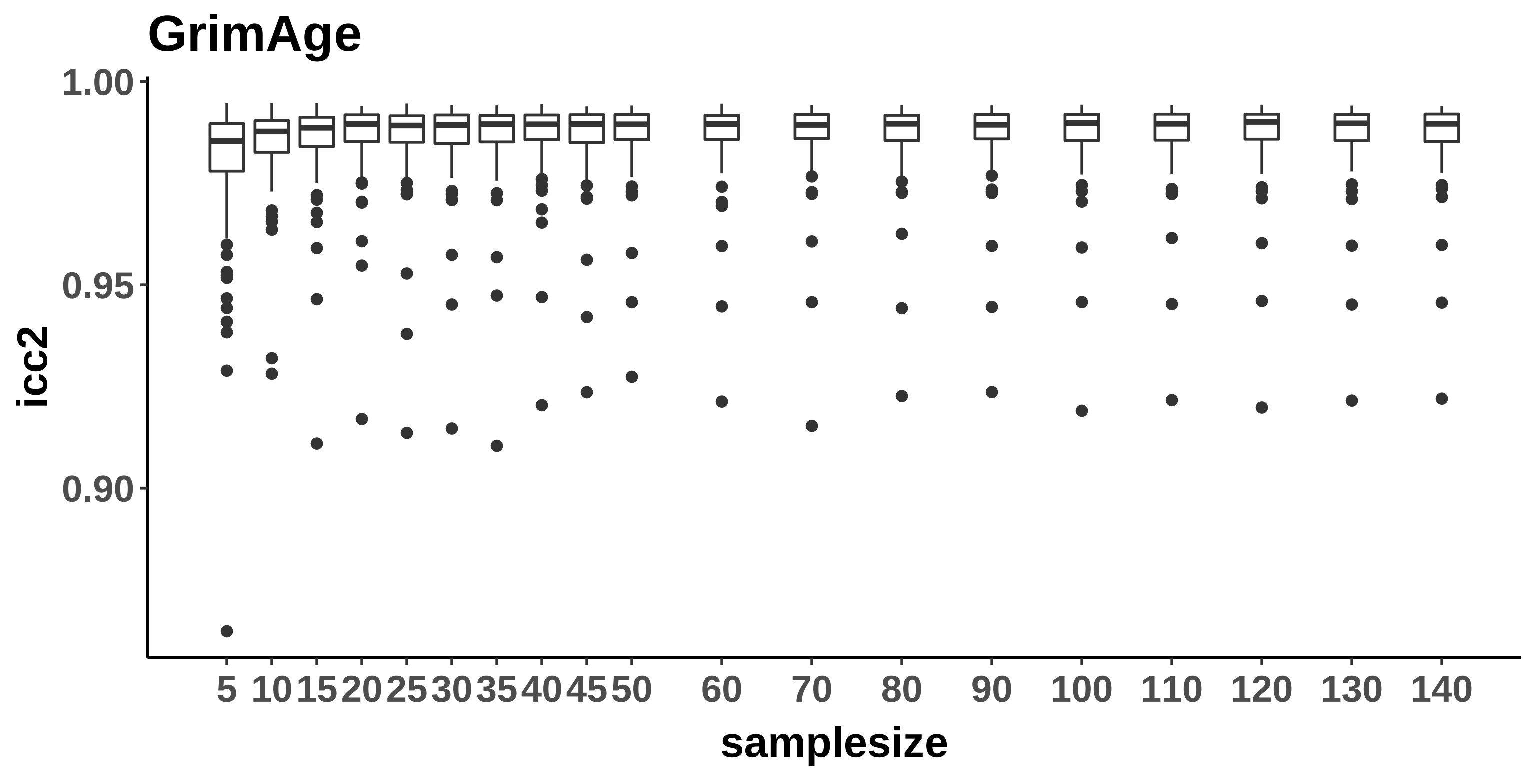

GrimAge
1.00
0.95
0.90
icc2
5
10
15
20
25
30
35
40
45
50
60
70
80
90
100
110
120
130
140
samplesize

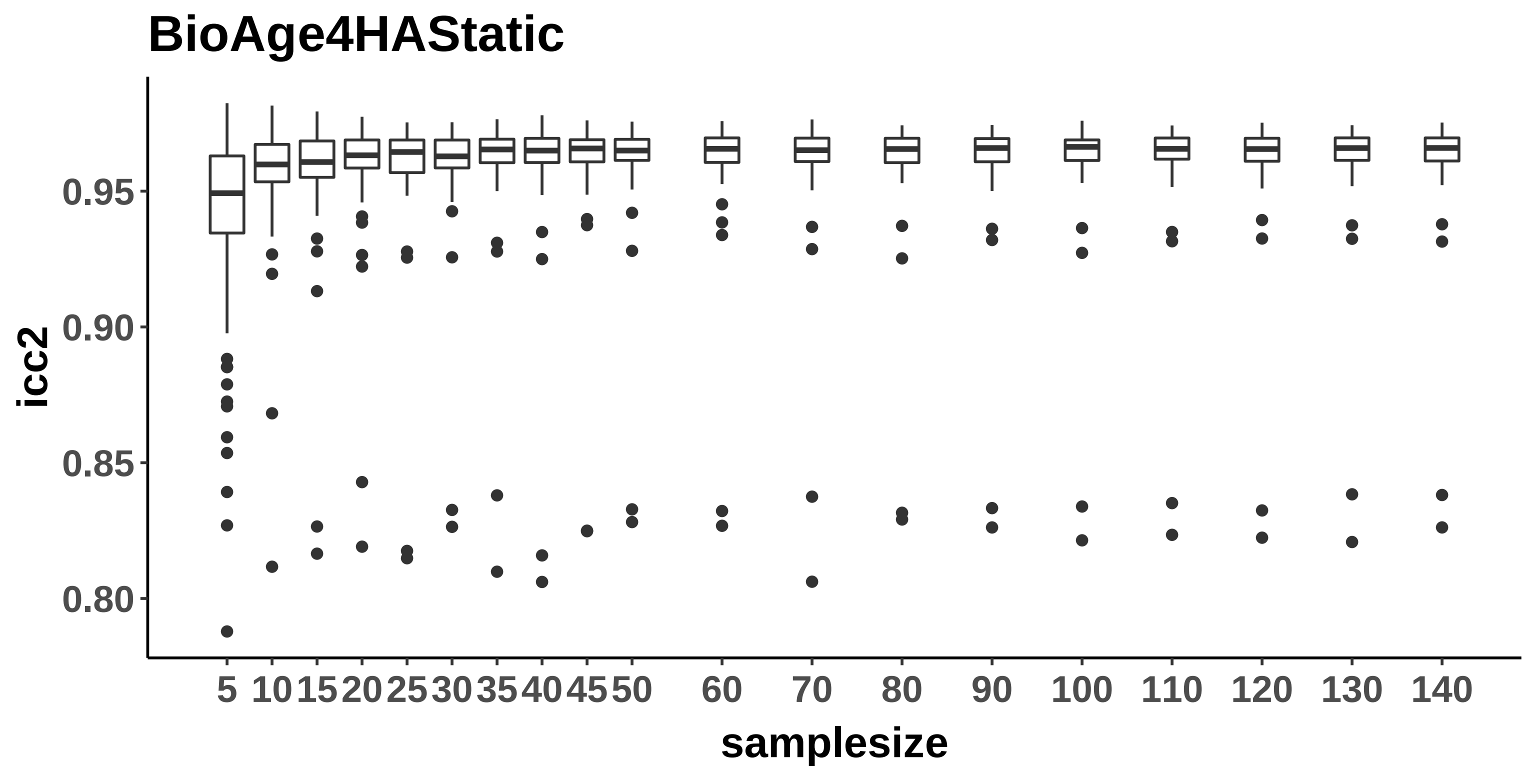
BioAge4HAStatic
icc2
0.95
0.90
0.85
0.80
5 10 15 20 25 30 35 40 45 50 60 70 80 90 100 110 120 130 140
samplesize
