# Supplementary material for: Significant variation in the performance of DNA methylation predictors across data preprocessing and normalization strategies
Source: Genome Biol. 2022 Oct 24;23:225. doi: 10.1186/s13059-022-02793-w (PMC9590227; doi:10.1186/s13059-022-02793-w)
Supplement: Supplementary file 8 — Additional file 8. Table that describes cohort characteristics. [file 13059_2022_2793_MOESM8_ESM.docx]

|  | Replicate Sample | General Sample |
| --- | --- | --- |
| Individuals (N) | 146 | 1,761 |
| Age (years) | 57.4 (SD = 12.3) | 56.1 |
| Female (%) | 62.6 % | 62.2 % |
| Death (%) | - | 15.1% |
| EPIC arrays (N) | 292 | 1,761 |

**Cohort characteristic of JHS.** In total, 1,907 individual and 2,053 850 EPIC array samples were included in our analysis. Our analyses were conducted in two subsets of this cohort, a subset that consist of only the technical replicate samples (n=146 individuals) and a subset that contains the remainder of the cohort. Shown above are standard cohort characteristics, including the percentage of individuals that died after follow-up for the general sample.
