# Supplementary material for: Significant variation in the performance of DNA methylation predictors across data preprocessing and normalization strategies
Source: Genome Biol. 2022 Oct 24;23:225. doi: 10.1186/s13059-022-02793-w (PMC9590227; doi:10.1186/s13059-022-02793-w)
Supplement: Supplementary file 9 — Additional file 9. Supplementary methods. [file 13059_2022_2793_MOESM9_ESM.docx]

**Supplementary Information**

Significant variation in performance of DNA methylation predictors across data preprocessing and normalization strategies.

Anil P.S. Ori^1^, Ake T Lu^2^, Steve Horvath^2,3^, Roel A Ophoff^1,2,4^

1. University of California Los Angeles, Center for Neurobehavioral Genetics, Semel Institute for Neuroscience and Human Behavior, Los Angeles, CA, USA

2. University of California Los Angeles, Department of Human Genetics, David Geffen School of Medicine, Los Angeles, CA, USA

3. University of California Los Angeles, Department of Biostatistics, Fielding School of Public Health, Los Angeles, CA, USA.

4. Erasmus University Medical Center, Department of Psychiatry, Rotterdam, The Netherlands.

**Supplementary Methods**

**1. DNAm probe quality control**

To assess data quality of the probes used by DNAm predictors, we assessed the level of detection of probes across samples. We used the detectionP() function in the minfi R package to calculate a detection P-value by comparing the total DNA signal (Methylated + Unmethylated) at each position to the background signal level, which is estimated using the negative control positions. We then assessed 1) if probes of predictors were detected above background, and 2) if there were any differences between probes of predictors and probes that were not included in the predictors.

Across DNAm predictors for which probe IDs were available (note that some predictors are proprietary and thus do not have probe ID information available), we identified a total of 8,150 probes of which 6,776 were unique and 6,693 on the EPIC array platform. Of these 6,693 DNAm predictor probes, 7 probes (0.10%) were detected at a P> 0.05 in more than 5% of all samples of the Jackson Heart study. These 7 probes are part of the BMI ("cg23505044", "cg21211039", " cg05176970"), BodyFat ("cg23505044", "cg05176970"), Education ("cg04460093"), HDL ("cg05176970"), WHR ("cg16558846"), PhenoAge ("cg08212685"), and the Horvath DNAmAge ("cg14329157") predictors. Probes of predictors (N=6,693) were on average detected more significantly than probes that were not part of the predictors (N=860,143). Across the full cohort, probes of predictors were on average not significantly detected (at detection P > 0.05) in 32 samples (1.7%, SD=23) compared to 34 samples (1.8%, SD = 35) when using probes that were not part of predictors, which was a significant difference (Wilcoxon Rank-Sum test, P=4.13e-09). This difference is likely because probes that are used by predictors are more likely to have biologically meaningful variation and therefore detected above background.

As only a small fraction of predictor probes (0.10%) was not detected above background signal, we decided to retain all probes in our analysis to minimize the loss of information from missing probes in calculated estimates from predictors.
