# Supplementary material for: Significant variation in the performance of DNA methylation predictors across data preprocessing and normalization strategies
Source: Genome Biol. 2022 Oct 24;23:225. doi: 10.1186/s13059-022-02793-w (PMC9590227; doi:10.1186/s13059-022-02793-w)
Supplement: Supplementary file 12 — Additional file 12. Peer review history. [file 13059_2022_2793_MOESM12_ESM.docx]

**Review history**

**First round of review**

**Reviewer 1**

Available tools for DNAm data processing are maturing and the landscape of DNAm predictors is growing. Reconciling the potential differences between data processing strategies used for predictor training with end user expectations and processing strategies with the results presented in this work has the potential to benefit the field. Ori et al evaluate methods for prediction using DNA methylation (DNAm) data comparing various normalization strategies using data from the Jackson Heart Study. In principle, the study provides a useful assessment of the impact of data processing steps on DNAm predictors. However, there are some questions about how the completeness of the processing tools used, background details could be improved to make the work more accessible, and the organization of results requires some careful reconsideration. Additional detail and clarity on actual recommendations to end users are needed.

The authors present some information on how technical variation can occur in the introduction, but seemingly miss an opportunity to note that DNAm data is arguably far less sensitive to many aspects that may introduce technical variation compared with other molecular data types (i.e. RNA-seq).

Use of the word "reliability" throughout could be somewhat problematic as it implies accuracy. Predictor accuracy is not being assessed. Consider using "consistency" or "replicability" instead.

Major processing pipelines Enmix, mifi and watermelon are included, Sesame [Zhou, >25k downloads from Bioconductor] appears not to be included. Why?

Existing studies and generally accepted practice include certain processing steps like probe type correction and batch correction. The rationale for use of all possible combinations of processing strategies is not clear.

The recommendation of using the best performing pipeline for specific predictors does not appear at all practical. Often, end users seek to determine multiple inferred phenotypes using DNAm predictors. Are the authors really recommending multiple data processing runs on the same data to assemble predictors and then only using one data set processed in one way to run downstream analyses?

Generally, some more attention to practical aspects of determining the processing pipeline could improve the manuscript.

- Specifically,
- Can the authors provide batch information for their replicates and comment on or include consideration of batch correction?
- Prediction performance for different cell types [5] are provided separately, in practice cell type proportions are determined simultaneously.
- Eliminating some low-likelihood processing strategies may be sound.
- Adding a figure and/or table with more detail about the major processing pipelines and types of processing would improve.
- The "Best pipeline" nomenclature in Table 1 needs a more detailed corresponding description for pipeline elements.
- What does "array compatability" really mean? Is it the data used for developing the predictor or the platform that data can be accepted from? HorvathAge [6] used 450K data but was limited to sites on the 27k array.
- No probes were filtered only samples, what are the data quality of probes used in DNAm predictors?

Figure 2 does not add much value, most of the data is in Table 1. Fig 2 could be supplemental. Table 1 could include another summary measure of dispersion to convey the information in fig 2.

The value and interpretation of Figure 3 is unclear.

It is very unfortunate that users would have to apply to obtain the data used in this manuscript when so much freely available data exists that does not require an application step.

**Reviewer 2**

I would like to begin by apologising to the authors for the delay in producing this review. This is an interesting manuscript by people who have a lot of experience with DNAm analysis, and systematic evaluation of methods is a very valuable thing, given that there is a plethora of processing options available. The authors have been extremely thorough in running a large number of processing pipelines, and also of predictors. If published this work would be influential, but in its current form I think it is misleading and there are some major unacknowledged deficiencies in the analysis.

The main finding is that different DNAm phenotypic predictors work best with different preprocessing pipelines. This is interesting because if there were a method that was clearly superior, you would expect it to perform best across the board. What we are instead seeing is a large number of reasonably good methods, when tested[1] with small numbers of samples [2] and probes[3], give results that differ in minor ways and with very limited generalisability[4].

[1] The main analysis is based on a subset of the Jackson Heart Study which consists of 146 technical replicate pairs. This is a valuable thing for isolating technical variance and a random effects model seems the sensible way to partition it. The authors use the ICC as their measure of 'reliability'. This is correct in that it measures the concordance of technical replicates, but there's no support for the implication that this predicts how reliably any of these predictors will predict phenotype. I believe the ICC measure is a poor one for the performance of normalisation methods, because it only rewards reduction of inter-pair variance but doesn't seem to consider how much possibly informative variance is sacrificed.

[2] The analysis is based on a single data set, the main analysis is on a small subset of it consisting of 146 individuals analysed twice. These technical replicates were from the same DNA extractions according to the methods session. There isn't further detail on how independent they are, but it looks like they are highly permanent technical replicates, further emphasizing that this analysis turns on tiny differences.

[3] The use of predictors is attractive in that these are measures that users will calculate. It does mean though that the evaluation the authors are undertaking are based on a tiny part of the array data. Furthermore, the analysis treats each of these predictors as a black box, not taking advantage of what these predictors can tell us about how the different pipelines are actually working. Do some predictors have weak, badly mapped/repetitive or SNP containing probes? As a minimum it would be very helpful if table 1 indicated the number of coefficients in each predictor (these are mostly given in the methods). A bit of consideration of the overlaps in probes used in the different measures would also be of interest.

[4] When users are considering what processing method to use, a major concern is often how much technical variance the method can deal with. This analysis uses a single, probably very well-behaved dataset. This probably has very little experimental variance to deal with and will not predict performance in data sets in the wild.

Figure 3 is a bit difficult to interpret, and mainly conveys that predictors vary in how many well-functioning probes they include.

The authors also include an analysis of predictions of one phenotype, all-cause mortality. I can see why they focus on it, but objectively it's one of the most difficult phenotypes to work with (how many of the Jackson cohort are actually dead?), and probably a poor index of the performance of other predictors.

**Authors’ response to reviewers**

Reviewer1’s Comments

Available tools for DNAm data processing are maturing and the landscape of DNAm predictors is growing. Reconciling the potential differences between data processing strategies used for predictor training with end user expectations and processing strategies with the results presented in this work has the potential to benefit the field. Ori et al evaluate methods for prediction using DNA methylation (DNAm) data comparing various normalization strategies using data from the Jackson Heart Study. In principle, the study provides a useful assessment of the impact of data processing steps on DNAm predictors. However, there are some questions about how the completeness of the processing tools used, background details could be improved to make the work more accessible, and the organization of results requires some careful reconsideration. Additional detail and clarity on actual recommendations to end users are needed.

R1-1. The authors present some information on how technical variation can occur in the introduction, but seemingly miss an opportunity to note that DNAm data is arguably far less sensitive to many aspects that may introduce technical variation compared with other molecular data types (i.e. RNA-seq).

Response: While we understand that comparison between technologies can give valuable insights, we decided to keep the focus on DNAm data in this paper.

R1-2. Use of the word "reliability" throughout could be somewhat problematic as it implies accuracy. Predictor accuracy is not being assessed. Consider using "consistency" or "replicability" instead.

Response: We agree with the reviewer. We have replaced the word reliability by consistency throughout the paper.

R1-3: Major processing pipelines Enmix, mifi and watermelon are included, Sesame [Zhou, >25k downloads from Bioconductor] appears not to be included. Why?

Response: While Sesame is an excellent software that facilitates a stepwise procedure to implement a range of different DNAm data analyses, including quality control steps and various inference methods, one of their main outputs is to mask probes due to failed hybridization to target DNA in regions of genomic deletions (often due to germline or somatic deletions). While this can decrease technical variation, particularly in DNAm data related to cancer as the authors described in their manuscript (PMID: 30085201), it is not necessarily relevant to all datasets. Furthermore, in analyses of DNAm-based predictors it is not recommended to mask or remove probes as missing probe information will impact the output estimates, which is why it is important to identify analytical strategies that remove unwanted technical variation without removing probes from the dataset. We have emphasized the importance of probe retention in the method section as follow:

“To perform a systematic evaluation of available data preprocessing and normalization strategies, we incorporated all methods that are available through the commonly used R packages minfi[36], wateRmelon[23], and ENmix[25]. These methods facilitate analytical strategies that help remove unwanted technical variation while allowing for probe retention, which is important as removal or masking of probes (i.e. missing data) will impact predictor estimates.”

R1-4. Existing studies and generally accepted practice include certain processing steps like probe type correction and batch correction. The rationale for use of all possible combinations of processing strategies is not clear.

Response: We chose to be inclusive in our approach by investigating all possible combinations of preprocessing and normalization methods so that we are unbiased in our systematic evaluation of the different analytical strategies. This has yielded important insights. For example, that for some predictors just using the raw data performs better compared to specific processing strategies. These insights would have been missed if we would have selected processing strategies a priori. We have clarified this in the method section of our manuscript:

“Within the same package, we implemented all possible combinations of background correction, dye-bias correction, probe correction, and data normalizations as was feasible within the structure of the package. To be inclusive and unbiased in our approach, we did not make a selection on methods a priori. In total, this yielded 101 strategies to prepare DNAm data (Table S2).”

R1-5. The recommendation of using the best performing pipeline for specific predictors does not appear at all practical. Often, end users seek to determine multiple inferred phenotypes using DNAm predictors. Are the authors really recommending multiple data processing runs on the same data to assemble predictors and then only using one data set processed in one way to run downstream analyses?

Response: we agree with the reviewer on this important point and that it is valuable to have a clear recommended processing strategy as most users will likely use one analytical strategy before performing downstream analyses. At the same time, it is also important to keep in mind that different analytical pipelines impact predictor performance differently and that, especially when comparing predictors, it is advised to evaluate their performance across multiple pipelines. We have adopted this point of the reviewer in our discussion section and now recommend the following:

“When only one processing strategy is desired, we recommend using the following stepwise sequence of ENmix methods; OOB background correction, RELIC dye bias correction, quantile normalization applied separately for methylated and unmethylated intensities of Infinium I and II probes, and RCP to correct for probe design type bias. This analytical pipeline achieved the best median rank across predictors. We further recommend careful quality control whether predictor estimates in downstream analyses are not dependent on (un)successful removal of technical variation, ideally with the use of replicates. In case no replicates are available, users can use the ICC values of each analytical pipeline reported in this study and assess if these ICC values correlate at all with their outcome of interest across the different processing strategies. While this strategy is sub-optimal compared to including study-specific replicates, it can help assess if technical variation impacts the analyses if one finds a significant correlation between ICC value calculated in our study and the estimated outcome measure in their study across analytical pipelines (like we show in Figure 4). Finally, as there is significant heterogeneity in the best performing analytical pipeline between predictors, we recommend evaluating multiple data preprocessing and normalization strategies when comparing the performance of DNAm predictors, ideally using the pipelines that yield their best performance, as reported in Table 1.”

R1-6. Generally, some more attention to practical aspects of determining the processing pipeline could improve the manuscript. Specifically, can the authors provide batch information for their replicates and comment on or include consideration of batch correction?

Response: we agree with the reviewer that including batch information and correction is important. We have therefore included a sensitivity analysis to the result section of the manuscript that evaluates how batch effects of array ID, array position, sample plate, and sample well impact are measures of consistency. We found that these batch effects explained less variance explained in predictor estimates when DNAm data was processed using analytical pipelines that yield higher consistency. This indicates that pipelines with higher consistency indeed are more successful in removing technical variation. We furthermore also show that the measures of consistency after regressing out batch effects from the predictor estimates strongly correlate with our original consistency measures without this correction (rho=0.81, P<2.2e-16). We however did find that including batch correction in the calculations of consistency produced lower ICC values compared to not including batch correction. We therefore do warrant to take caution when considering regressing out these batch effects as biologically meaningful information may also get lost, which we mention in the discussion of the manuscript. We have furthermore added batch information to Table S3 as the reviewer requested.

Text added to the results:
“Best performing analytical pipelines are less impacted by batch effects
Next, we assessed how corrections for batch effects impacted our measures of consistency across predictors across analytical pipelines. We included four covariates with potential batch effects in our analysis, i.e. array ID, array position, sample plate, and sample well. Such data was available for 1,888 samples in the full JacksonHeart dataset. Across pipelines and predictors (N=4141), batch effects collectively explained a median of 4.45% (Q1-Q3 = 1.48%-7.20%) of the variance in predictor estimates. Batch effects explained less variance in output estimates when the best performing pipelines were applied (median=3.37%, Q1-Q3 = 1.35%-7.23%) compared to the worst performing pipelines (median=5.80%, Q1-Q3 = 3.08%-9.43%). This indicates that analytical pipelines that yield higher agreement between technical replicates are more successful in removing technical variation introduced by batch effects. This is further emphasized by a negative correlation between the variance explained by batch effects and the calculated ICC across predictors and pipelines (N=4141, rho=-0.05, P=3.7e-04). This relationship is stronger in the worst performing pipelines (N=41, rho= -0.37, P=0.02) and not present within the best performing pipelines (N=41, rho=0.09, P=0.58). After regressing out batch effects from the predictor estimates, we found a strong correlation with the consistency measured without correcting for batch effects (N=4141, rho=0.81, P<2.2e-16, see Figure S3). Correction for batch effects on average produced lower consistency (median ICC=0.81, Q1-Q3=0.76-0.84) compared to unadjusted predictor estimates (median ICC=0.96, Q1-Q3=0.91-0.98).”

Text added to the discussion:
“We also found that the best performing analytical pipelines are less impacted by batch effects, confirming that technical variation is more successfully removed, compared to pipelines that yield lower consistency. We furthermore recommend careful consideration when adjusting for batch effects as it can result in biologically meaningful information being lost. In our analyses, we found that measures of consistency were on average lower when batch effects were corrected for by regressing out the effect array chip, array position, and laboratory plate compared to the unadjusted predictor estimates. Batch correction methods that can consider a trade-off between batch noise and signal preservation could help users if such crude statistical adjustments are needed (Oytam et al. 2016). Further development of data preprocessing and normalization methods that improve our ability to remove technical variation from DNAm estimates is an important avenue to pursue as well.”

R1-7. Prediction performance for different cell types [5] are provided separately, in practice cell type proportions are determined simultaneously.

Response: we have emphasized in the method section that cell type proportions are estimated jointly:

“These six cell types were estimated jointly using the estimateCellProp(refdata="FlowSorted.Blood.EPIC", nprobes=50) function of the ENmix R package.”

R1-8. Eliminating some low-likelihood processing strategies may be sound.

Response: we chose to be inclusive in our approach and did not want to select analytical strategies based on a priori information as no systematic evaluation of data preprocessing and normalization strategies at this scale has been conducted for DNAm data. See also our response to comment R1-4.

R1-9. Adding a figure and/or table with more detail about the major processing pipelines and types of processing would improve.

Response: full details on our analytical pipelines and methods implemented are described in Table S2. We have furthermore clarified the details of the best performing pipeline for each predictor in Table 1 of the manuscript (see also comment R1-10).

R1-10. The "Best pipeline" nomenclature in Table 1 needs a more detailed corresponding description for pipeline elements.

Response: we agree with the reviewer that our previous nomenclature was suboptimal and have clarified this in Table 1 of the manuscript. Instead of “Enmix: oob_mean_q3_rcp” for example, Table 1 now states “ENmix: bg=oob, dye=mean, norm=q3, probe=rcp”. This refers to the stepwise sequence of background correction, dye bias correction, normalization, and probe type bias correction. In the caption of Table 1, we have added more clarification for the readers and refer to Table S2 for full details on each method as well;

“Table 1. Overview of predictor consistency and best performing data processing pipelines. Shown are general information on each DNAm-based predictor alongside their corresponding ICC statistics. The name of the predictor, the phenotype it is trained on, the array platform it can be applied on, and the number of predictor probes (if available) are listed on the left side of the table. ICC statistics are listed on the right side of the table. The ICC quantifies the degree of absolute agreement between estimator values of a pair of technical replicates. For each predictor, across 101 pipelines, the median, minimum, and maximum ICC are listed. Predictors are ranked by the maximum ICC. The final column reports methodological details of the best performing data processing pipelines (i.e., the pipeline with the highest consistency). Bg=background correction; dye=dye bias correction, norm=normalization method, probe=probe type bias correction. Full details on analytical pipelines and how they were implemented are available in Table S2.”


R1-11. What does "array compatability" really mean? Is it the data used for developing the predictor or the platform that data can be accepted from? HorvathAge [6] used 450K data but was limited to sites on the 27k array.

Response: “array compatability” means the platform the data can be accepted from and thus the platform the DNAm algorithm can be applied on. We agree that this is confusion and have changed the column header to just “Array” and describe its meaning in the caption of table 1;

“Table 1. Overview of predictor consistency and best performing data processing pipelines. Shown are general information on each DNAm-based predictor alongside their corresponding ICC statistics. The name of the predictor, the phenotype it is trained on, and the array platform it can be applied on are listed on the left side of the table. ICC statistics are listed on the right side of the table.”


R1-12. No probes were filtered only samples, what are the data quality of probes used in DNAm predictors?

Response: We agree with the reviewer that the data quality of the probes used by predictors is important to check. To assess the quality of probes, we calculated the detection P-value of all probes across the Jackson Heart Study DNAm samples. Using standard threshold criteria, we found 7 predictor probes (0.10% of 6,693 total predictor probes) to be detected at P > 0.05 in more than 5% of samples. Overall, we found predictor probes to be of higher quality than non-predictor probes. We have added this extra analysis to the supplementary material and refer to it in our method section of the manuscript. In addition, we now also acknowledge in our discussion section that future work should be done on how to deal with low quality predictor probes, for example by using imputation-based approaches.

Text in method section of manuscript:
“No probes were filtered out to minimize missing probes in downstream DNAm prediction analysis. We did check the quality of probes used by predictors to calculate estimates and found these overall to be of good quality (see supplementary methods).”

Text in supplementary methods:
“To assess data quality of the probes used by DNAm predictors, we assessed the level of detection of probes across samples. We used the detectionP() function in the minfi R package to calculate a detection P-value by comparing the total DNA signal (Methylated + Unmethylated) at each position to the background signal level, which is estimated using the negative control positions. We then assessed 1) if probes of predictors were detected above background, and 2) if there were any differences between probes of predictors and probes that were not included in the predictors.
Across DNAm predictors for which probe IDs were available (note that some predictors are proprietary and thus do not have probe ID information available), we identified a total of 8,150 probes of which 6,776 were unique and 6,693 on the EPIC array platform. Of these 6,693 DNAm predictor probes, 7 probes (0.10%) were detected at a P> 0.05 in more than 5% of all samples of the Jackson Heart study. These 7 probes are part of the BMI ("cg23505044", "cg21211039", " cg05176970"), BodyFat ("cg23505044", "cg05176970"), Education ("cg04460093"), HDL ("cg05176970"), WHR ("cg16558846"), PhenoAge ("cg08212685"), and the Horvath DNAmAge ("cg14329157") predictors. Probes of predictors (N=6,693) were on average detected more significantly than probes that were not part of the predictors (N=860,143). Across the full cohort, probes of predictors were on average not significantly detected (at detection P > 0.05) in 32 samples (1.7%, SD=23) compared to 34 samples (1.8%, SD = 35) when using probes that were not part of predictors, which was a significant difference (Wilcoxon Rank-Sum test, P=4.13e-09). This difference is likely because probes that are used by predictors are more likely to have biologically meaningful variation and therefore detected above background.
As only a small fraction of predictor probes (0.10%) was not detected above background signal, we decided to retain all probes in our analysis to minimize the loss of information from missing probes in calculated estimates from predictors.”

Text in discussion:
“Third, we included all DNAm probes of predictors in our analyses and did not assess how accounting for data quality of probes impact measures of consistency. Overall predictor probes were of good quality in the Jackson Heart Study sample. Future studies should however investigate this further, for example by using imputation-based methods to impute probes that need to be excluded because of lower data quality or bad mapping, as not all datasets will have good quality data.”


R1-13. Figure 2 does not add much value, most of the data is in Table 1. Fig 2 could be supplemental. Table 1 could include another summary measure of dispersion to convey the information in fig 2.

Response: we agree and have moved Figure 2 to the supplementary materials as Table 1 indeed already conveys its information.

R1-14. The value and interpretation of Figure 3 is unclear.

Response: Figure 3 (now Figure 2 in the revised manuscript) shows the correlation structure of measures of consistency between predictors across analytical pipelines. It visualizes the degree of heterogeneity of pipeline performance across predictors, which is an important take-home message for readers. We have clarified this in the caption of the figure;

“Figure 2. DNAm predictors have a moderate degree of concordance in performance between pipelines. Shown is a clustered correlation heatmap of consistency across pipelines that visualizes the heterogeneity in pipeline performance between predictors. The color coding depicts Spearman’s rho and clustering is performed using hierarchical clustering. Only correlations with a P-value < 0.01 are colored.”

R1-15. It is very unfortunate that users would have to apply to obtain the data used in this manuscript when so much freely available data exists that does not require an application step.

Response: we agree with the reviewer but are unfortunately limited by data privacy regulations of the Jackson Heart Study. We did inquire with the Jackson Heart Study research office whether DNAm data of the technical replicates can be deposited on a public repository, but this was unfortunately not possible. It is however possible to request both the DNAm data as well as the phenotype information from the Jackson Heart Research Office. In the “Availability of data and material” in our Declarations section we have clarified this:

“All DNAm and phenotype data were obtained from the Jackson Heart Study. The data are not publicly available because they contain information that could compromise the study participant privacy and consent. Data can be obtained by submitting a request to the Jackson Heart Study research office: https://www.jacksonheartstudy.org/Research/Study-Data/Data-Access.”


Reviewer2’s Comments

I would like to begin by apologising to the authors for the delay in producing this review. This is an interesting manuscript by people who have a lot of experience with DNAm analysis, and systematic evaluation of methods is a very valuable thing, given that there is a plethora of processing options available. The authors have been extremely thorough in running a large number of processing pipelines, and also of predictors. If published this work would be influential, but in its current form I think it is misleading and there are some major unacknowledged deficiencies in the analysis.

The main finding is that different DNAm phenotypic predictors work best with different preprocessing pipelines. This is interesting because if there were a method that was clearly superior, you would expect it to perform best across the board. What we are instead seeing is a large number of reasonably good methods, when tested[1] with small numbers of samples [2] and probes[3], give results that differ in minor ways and with very limited generalisability[4].

R2-1. The main analysis is based on a subset of the Jackson Heart Study which consists of 146 technical replicate pairs. This is a valuable thing for isolating technical variance and a random effects model seems the sensible way to partition it. The authors use the ICC as their measure of 'reliability'. This is correct in that it measures the concordance of technical replicates, but there's no support for the implication that this predicts how reliably any of these predictors will predict phenotype. I believe the ICC measure is a poor one for the performance of normalisation methods, because it only rewards reduction of inter-pair variance but doesn't seem to consider how much possibly informative variance is sacrificed.

Response: These are great points. We implemented the reviewer’s suggestions as follows. First, in response to this comment and that of another reviewer, we have replaced the word "reliability" by "consistency" throughout our manuscript since our aim was to evaluate consistency/replicability. Second, we now added the following discussion of limitations to the Discussion section:

“Fourth, the ICC is limited in that it focuses on the reduction of inter-pair variance and does not track the loss of possibly informative variance. Future studies may evaluate the normalization pipelines with respect to predictive accuracy for morbidity and mortality risk as well.”

R2-2. The analysis is based on a single data set, the main analysis is on a small subset of it consisting of 146 individuals analysed twice. These technical replicates were from the same DNA extractions according to the methods session. There isn't further detail on how independent they are, but it looks like they are highly permanent technical replicates, further emphasizing that this analysis turns on tiny differences.

Response: the reviewer is correct in that the replicate pairs used are independent technical replicates but not biological replicates (i.e. each pair originates from the same DNA extraction). It is very likely that a dataset of biological replicates (assuming all other things being equal) will show greater differences. We have further clarified this as a limitation in our discussion section:

“Our study comes with limitations. First, we measured consistency using technical replicates in one study. A different cohort or different types of repeated measures, for example biological replicates, may yield different outcomes. Ideally, one would use study-specific replicate samples and assess if similar best practices are achieved or if alternative strategies are more appropriate to remove technical variation most optimally for that specific study.”

R2-3. The use of predictors is attractive in that these are measures that users will calculate. It does mean though that the evaluation the authors are undertaking are based on a tiny part of the array data. Furthermore, the analysis treats each of these predictors as a black box, not taking advantage of what these predictors can tell us about how the different pipelines are actually working. Do some predictors have weak, badly mapped/repetitive or SNP containing probes? As a minimum it would be very helpful if table 1 indicated the number of coefficients in each predictor (these are mostly given in the methods). A bit of consideration of the overlaps in probes used in the different measures would also be of interest.

Response: These are excellent points by the reviewer. To assess the quality of probes, we calculated the detection P-value of all probes across the Jackson Heart Study DNAm samples. Using standard threshold criteria, we found 5 predictor probes (0.07% of 6,693 total predictor probes) to be detected at P > 0.05 in more than 5% of samples. Overall, we found predictor probes to be of higher quality than non-predictor probes. We have added this extra analysis to the supplementary material and refer to it in our method section of the manuscript. In addition, we now also acknowledge in our discussion section that future work should be done on how to deal with low quality or poorly mapped predictor probes, for example by using imputation-based approaches. We furthermore added the number of probes of each predictor to Table 1, as the reviewer requested.

Text in method section of manuscript:
“No probes were filtered out to minimize missing probes in downstream DNAm prediction analysis. We did check the quality of probes used by predictors to calculate estimates and found these overall to be of good quality (see supplementary methods).”

Text in supplementary methods:
“To assess data quality of the probes used by DNAm predictors, we assessed the level of detection of probes across samples. We used the detectionP() function in the minfi R package to calculate a detection P-value by comparing the total DNA signal (Methylated + Unmethylated) at each position to the background signal level, which is estimated using the negative control positions. We then assessed 1) if probes of predictors were detected above background, and 2) if there were any differences between probes of predictors and probes that were not included in the predictors.
Across DNAm predictors for which probe IDs were available (note that some predictors are proprietary and thus do not have probe ID information available), we identified a total of 8,150 probes of which 6,776 were unique and 6,693 on the EPIC array platform. Of these 6,693 DNAm predictor probes, 7 probes (0.10%) were detected at a P> 0.05 in more than 5% of all samples of the Jackson Heart study. These 7 probes in the BMI ("cg23505044", "cg21211039", " cg05176970"), BodyFat ("cg23505044", "cg05176970"), Education ("cg04460093"), HDL ("cg05176970"), WHR ("cg16558846"), PhenoAge ("cg08212685"), and the Horvath DNAmAge ("cg14329157") predictors. Probes of predictors (N=6,693) were on average detected more significantly than probes that were not part of the predictors (N=860,143). Across the full cohort, probes of predictors were on average not significantly detected (at detection P > 0.05) in 32 samples (1.7%, SD=23) compared to 34 samples (1.8%, SD = 35) when using probes that were not part of predictors, which was a significant difference (Wilcoxon Rank-Sum test, P=4.13e-09). This difference is likely because probes that are used by predictors are more likely to have biologically meaningful variation and therefore detected above background.
As only a small fraction of predictor probes (0.10%) was not detected above background signal, we decided to retain all probes in our analysis to minimize the loss of information from missing probes in calculated estimates from predictors.”

Text in discussion:
“Third, we included all DNAm probes of predictors in our analyses and did not assess how accounting for data quality of probes impact measures of consistency. Overall predictor probes were of good quality in the Jackson Heart Study sample. Future studies should however investigate this further, for example by using imputation-based methods to impute probes that need to be excluded because of lower data quality or bad mapping, as not all datasets will have good quality data.”

R2-4. When users are considering what processing method to use, a major concern is often how much technical variance the method can deal with. This analysis uses a single, probably very well-behaved dataset. This probably has very little experimental variance to deal with and will not predict performance in data sets in the wild.

Response: we agree with the reviewer that our findings are based one study and that it is yet to be determined how our findings generalize across datasets with differing technical variance or with different type of replicates, for example biological replicates. We note this as a limitation in the discussion section of our manuscript and give recommendations on how future studies could include different type of replicates (see also point R2-2).

Text in discussion section:
“Our study comes with limitations. First, we measured consistency using technical replicates in one study. A different cohort or different types of repeated measures, for example biological replicates, may yield different outcomes. Ideally, one would use study-specific replicate samples and assess if similar best practices are achieved or if alternative strategies are more appropriate to remove technical variation most optimally for that specific study. If future studies have the means to include replicate samples, they should aim to include at least ten replicate pairs. We determined that for most predictors a sample size of ten replicate pairs can already provide meaningful insights into their consistency and replicability.”


R2-5. Figure 3 is a bit difficult to interpret, and mainly conveys that predictors vary in how many well-functioning probes they include.

Response: Figure 3 (now Figure 2 in the revised manuscript) shows the correlation structure of measures of consistency between predictors across analytical pipelines. It visualizes the degree of heterogeneity of pipeline performance across predictors, which is an important take-home message for readers. We have clarified this in the caption of the figure;

“Figure 2. DNAm predictors have a moderate degree of concordance in performance between pipelines. Shown is a clustered correlation heatmap of consistency across pipelines that visualizes the heterogeneity in pipeline performance between predictors. The color coding depicts Spearman’s rho and clustering is performed using hierarchical clustering. Only correlations with a P-value < 0.01 are colored.”

R2-6. The authors also include an analysis of predictions of one phenotype, all-cause mortality. I can see why they focus on it, but objectively it's one of the most difficult phenotypes to work with (how many of the Jackson cohort are actually dead?), and probably a poor index of the performance of other predictors.

Response: we understand the concerns of the reviewer and have adjusted the manuscript as follows: 1) we have added that 15% of the Jackson Heart Study participants are deceased to the text to make this clearer:

“To relate DNAm predictor estimates with mortality risk (15% of individuals in JHS are deceased), a Cox proportional hazards regression model was fitted using the coxph() function of the survival package (v3.2).”

Second, we stated in the limitation section of our Discussion that we did not investigate downstream phenotypic analyses of other predictors and that further research on this, using datasets that include the relevant phenotypes, is warranted:

“Finally, we assessed the impact of (un)successful removal of unwanted technical variation on downstream phenotypic analyses of DNAm clocks and mortality risk, which may yield different results for other phenotypes that were not measured in JHS.”

**Second round of review**

**Reviewer 1**

No further comments
